# Supplementary material for: Strobilurin biosynthesis in Basidiomycete fungi
Source: Nat Commun. 2018 Sep 26;9:3940. doi: 10.1038/s41467-018-06202-4 (PMC6158276; doi:10.1038/s41467-018-06202-4)
Supplement: Supplementary file 1 — Supplementary Information [file 41467_2018_6202_MOESM1_ESM.pdf]

Strobilurin Biosynthesis in Basidiomycete Fungi.

Nofiani et al.

corresponding author: russell.cox@oci.uni-hannover.de

Supplementary Information

Supplementary Figures	2
Supplementary Tables	37
Supplementary Methods	43
Supplementary References	61

Supplementary Figures

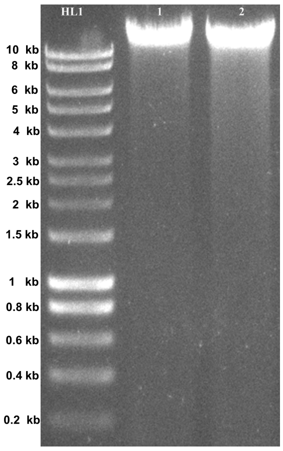

Supplementary Figure 1. Gel image showing the gDNA preparation used for genome sequencing: **HL1,** Hyperladder^{TM} 1kb; **Lane 1**, *S. tenacellus*; **Lane 2**, *S. lutea* F23523.

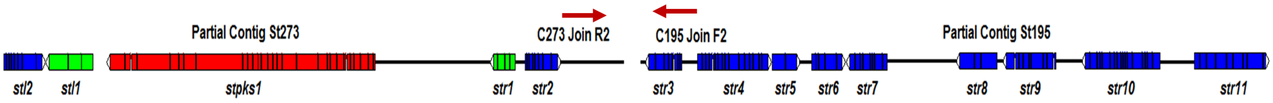

Supplementary Figure 2. Primer positions used to amplify a missing sequence between contigs St-273 and St-195.

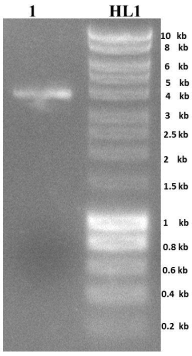

Supplementary Figure 3. PCR product of ~4kb linking contigs St273 and St195.

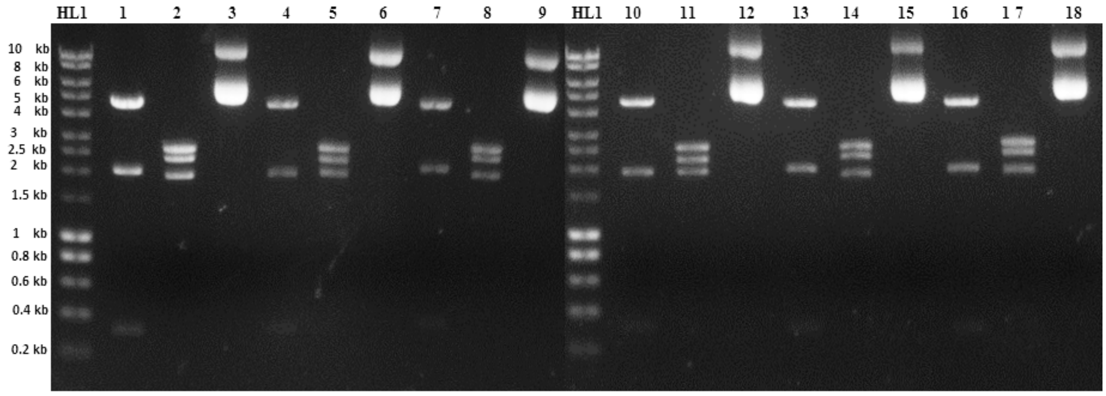

Supplementary Figure 4. Restriction digest analysis of recombinant pJET1.2 containing a PCR product joining contig 273 and contig 195: **Lanes 1, 4, 7, 10, 13, and 16.** The recombinant plasmids cut with PstI/Not1 (309 bp, 3302 bp, 3633bp); **Lanes 2, 5, 8, 11, 14, and 17**, The recombinant plasmids cut with PstI/Xba1 (368 bp, 615 bp, 2641 bp, 2792 bp). Lanes 3,6,9,12,15,18 show the undigested recombinant plasmids.

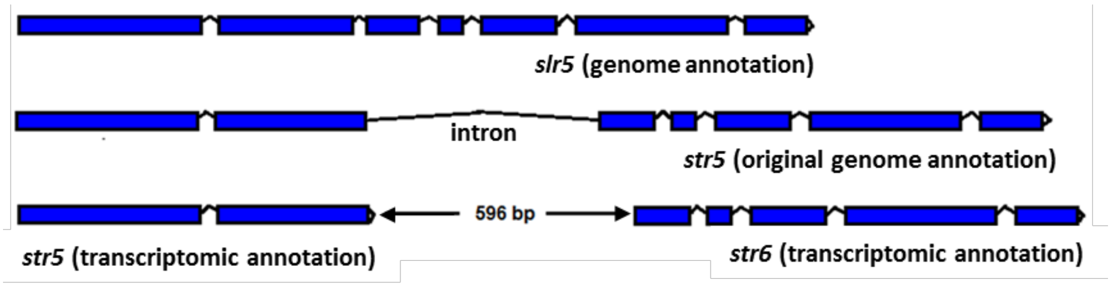

Supplementary Figure 5. Annotation of *slr5*, *str5*, and *str6* based on original genomic and subsequent transcriptomic data.

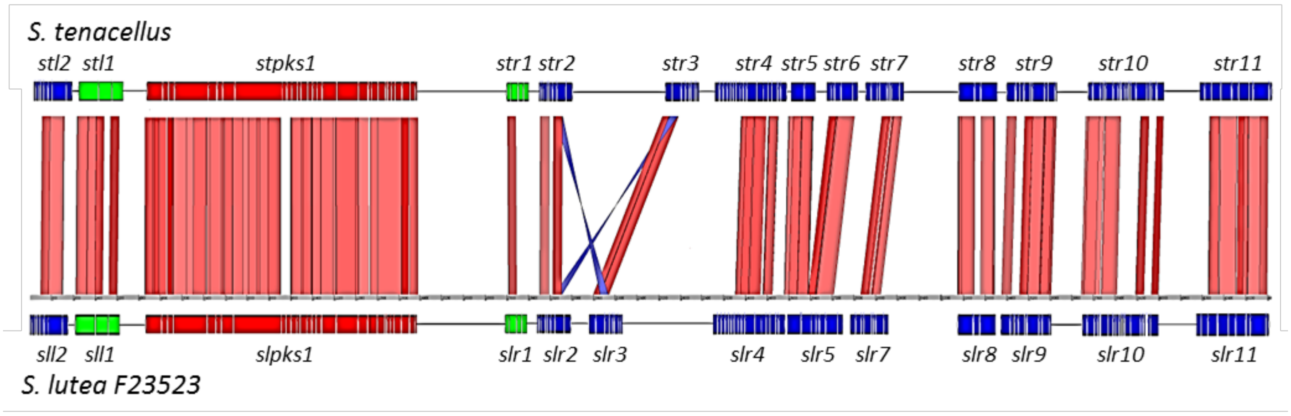

Supplementary Figure 6. Artemis[1] Comparison of the predicted strobilurin BGC of *S. tenacellus* and *S. lutea* F23523 using gene names from Supplementary Table 5.

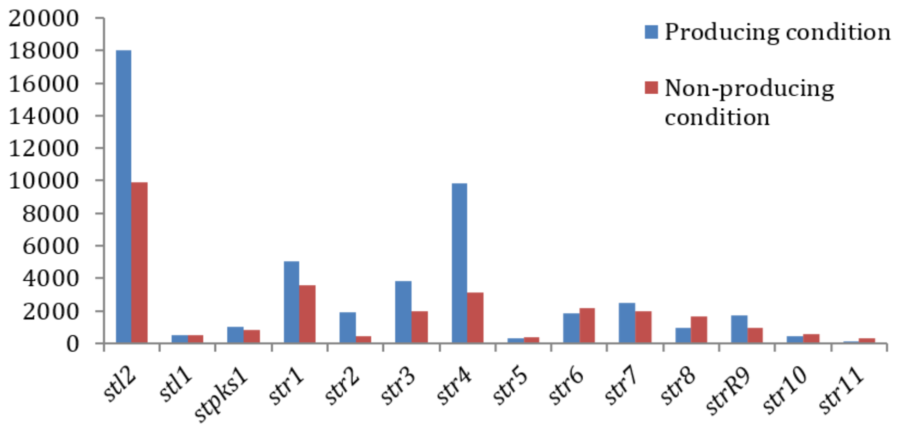

Supplementary Figure 7. Expression level of the predicted SBGC of *S. tenacellus* under producing (CGC medium, 6 days) and non-producing (YM media, 6 days) conditions. Units: Reads Per Kilobase of transcript per Million mapped reads (RPKM). A single replicate was used.

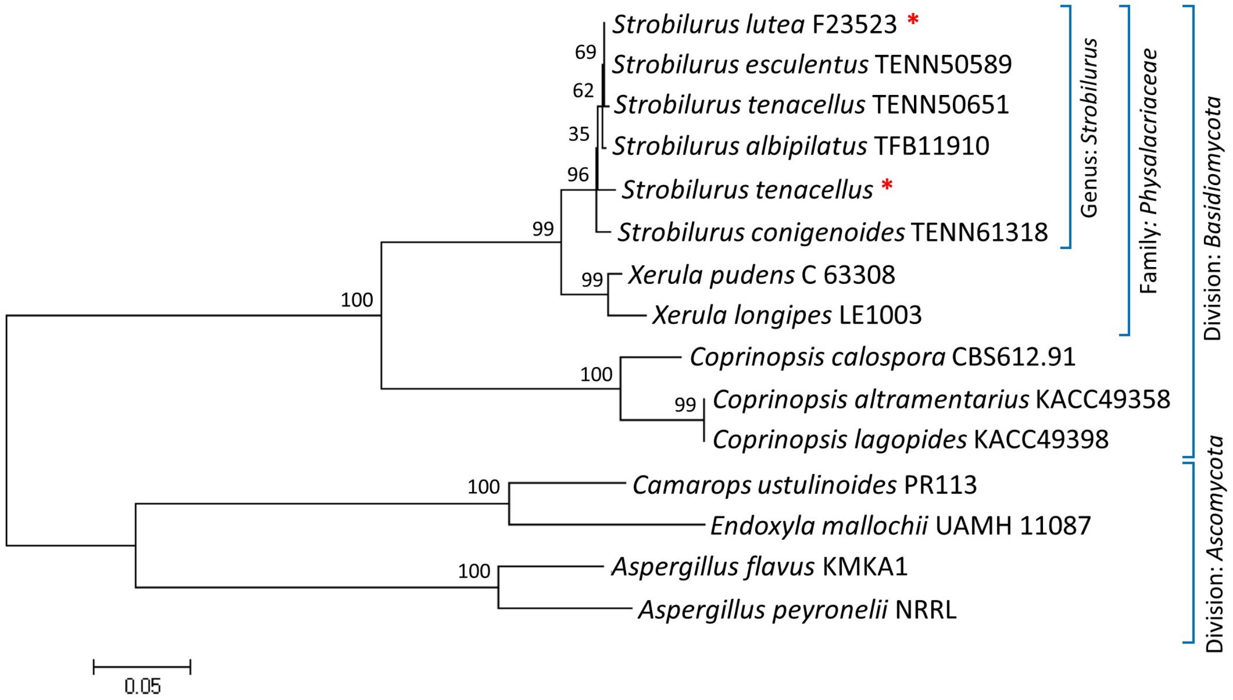

Supplementary Figure 8. A phylogenetic tree of Internal transcriped Spacer (ITS) sequences constructed in MEGA4 using the neighbour-joining method. Bootstrap values from 1000 replicates are shown next to the branches. Sequences generated in this work for *Strobilurus tenacellus* and the newly named *Strobilurus lutea* (previously *Bolinea lutea*) are marked with a red asterisk. The other sequences were obtained from NCBI.

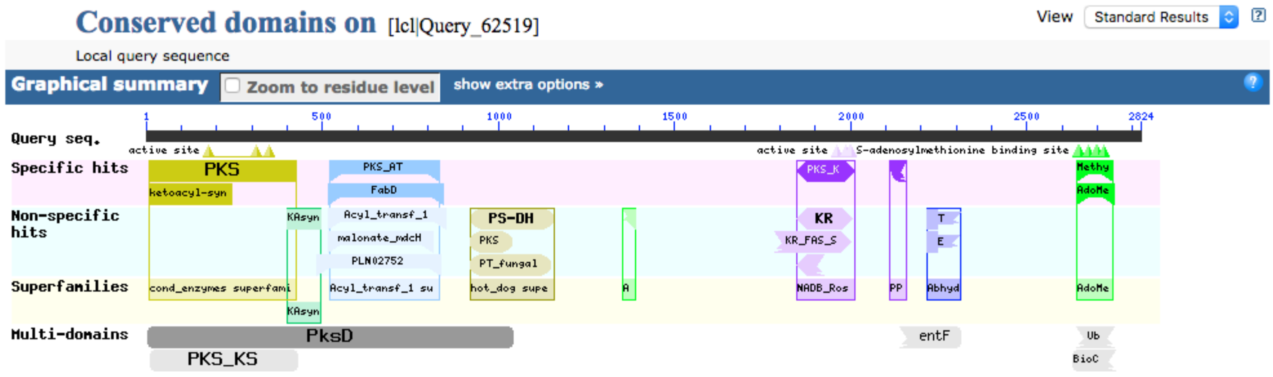

Supplementary Figure 9. Domain analysis of StPKS1 using the NCBI CDD (Conserved Domain Database).[2]

```
                                 KS                                           KS
StPKS1             -------MSPTAEIPKPVAVVGISAEFPSGTLSDANFDHQSFFDFLLSGKDAVERIPKDR
SlPKS1             -------MTPPAADLKPVAVVGISAEFPSGTLSDANFDHQSFFEFLLSGKDAAERIPKDR
mFASSusScrofa      --------------MEEVVIAGMSGKLPESENL------EEFWANLIGGVDMVTAD-DRR
mFASrat            --------------MEEVVIAGMSGKLPESENL------QEFWANLIGGVDMVTDD-DRR
MFSQTKS            MTAMDEY-QHHEDATIPIAIIGMSCRFPGNATS-----PEKLWELCAEGRSAWSKIPKSR
TENS               MSPMKQNESESHSVSEPIAIIGSAYRFPGGCNT-----PSKLWDLLRQPRDILKEIDPER
                                    :.: * : .:* .         ..::       .        *
                   KS                                                        KS
StPKS1             FNIDGWQGSHLGQILPE--------DACFLKNVHLFDHFEFGISSKDALTMGAGTRKLVE
SlPKS1             FNIDGWQGSHLGQILPE--------DACFLKDVHLFDHFEFGISSKDALTMGAGTRKLVE
mFASSusScrofa      WK-AGLYGLPR--------------RMGKLKDLSRFDASFFGVHSKQANTMDPQLRMLLE
mFASrat            WK-AGLYGLPK--------------RSGKLKDLSKFDASFFGVHPKQAHTMDPQLRLLLE
MFSQTKS            FRQEGFYNPNAERVGTVSLDYFGFGGHFLEEDPSLFDASFFNLSAEAAKTMDPQFRLQLE
TENS               LNLRRYYHPDGETHGSTDVANK---AYTLEEDISRFDASFFGISPLEAASMDPQQRTLLE
                    .                            ::   **   *.:    * :*.   *  :*
                   KS                                                        KS
StPKS1             HSFLALLDSGINCRSQNVAAFSSAVAFD-----LLSAADADEFEPRDGFGGGAAAVANRI
SlPKS1             HSFLALLDSGINSRSQNVAAFSSAVAFD-----LLSAADADEFEPRDGFGGGAAAVANRI
mFASSusScrofa      VTYEAIVDGGINPASLRGTSTGVWVGVSSSDASEALSRDPETLVGYSMIGCQRAMMANRL
mFASrat            VSYEAIVDGGINPASLRGTNTGVWVGVSGSEASEALSRDPETLLGYSMVGCQRAMMANRL
MFSQTKS            SVYEAMESAGITLEHIAGSDTSVYAGACFRDYHDSLVRDPDLVPRFLLTGNGAAMSSNRI
TENS               VVYESTETAGIPLDKLRGSLTSVHVGVMTTDWAQMQRRDPETMPQYTATGIASSIISNRI
                     : :   .**       :  .  ..            * : .      *   :  :**:
                   KS           ***********                                  KS
StPKS1             SYQLDLLGPSIPVDTACSSSLMALHLGVQSLRAGECEAAVIGGSQINHRFLDWIFYSQLS
SlPKS1             SYQLDLLGPSIPVDTACSSSLMALHLGVQSLRAGECEAAVIGGSQINHRFLDWIFYSQLS
mFASSusScrofa      SFFFDFKGPSITIDTACSSSLLALQSAYQAIRGGECSAAVVGGLNVLLKPNSSLQFMKLG
mFASrat            SFFFDFKGPSIALDTACSSSLLALQNAYQAIRSGECPAATVGGINLLLKPNTSVQFMKLG
MFSQTKS            SYFYDLHGASMTVDTGCSTTLTALHLACQGLRNRESKTSIVTGANVILNPDMFVTMSSLG
TENS               SYIFDLKGASETIDTACSSSLVALHNAARALQSGDCEKAIVAGVNLILDPDPFIYESKLH
                   *:  *: * *  :**.**::* **: . :.::  :.  : : * ::       :   .*
                   KS                                                        KS
StPKS1             ILSPGGKSIPFDSSADGFGRGEAVVVLVVKLLEDAIRDGDKIYATVLNTAVNST-GSAGP
SlPKS1             ILSPRGKSIPFDSSADGFGRGEAVVVLVVKLLEDAIRDGDKIYATVLNTAVNST-GSAGP
mFASSusScrofa      MLSQDGTCRSFDAEGTGYCRAEAVVAVLLTKKSLARRV----YATILNAGTNTDGSKEQG
mFASrat            MLSPDGTCRSFDDSGNGYCRAEAVVAVLLTKKSLARRV----YATILNAGTNTDGCKEQG
MFSQTKS            LLGPEGKSHTFDARANGYGRGEGIATVIIKRLDEALAAQDPIRCIIRGTALNQD-GKTAT
TENS               MLSPDARSRMWDAAANGYARGEGAAAVVLKTLGHALRDGDRIEGVIRSTFVNSD-GLSSG
                   :*.  .  .  :*  . *: *.*. ..:::.    *          :  .:  *
                   KS                                                        KS
StPKS1             VKTPIAESQAAAMLTAYKGIGRSPSE--ADFIECHATGTSVGDPVEANWVGNHF------
SlPKS1             VKTPIAESQAAAMLTAYKGIGRSPSE--VDFVECHATGTSVGDPVEANWVGKHF------
mFASSusScrofa      VTFPSGDVQEQLIRSLYAPAGPDPES--LEYIEAHGTGTKVGDPQELNGIVNAL------
mFASrat            VTFPSGEAQEQLIRSLYQPGGVAPES--LEYIEAHGTGTKVGDPQELNGITRSL------
MFSQTKS            LTSPSQTAQSDLIRACYRAAALDPND--TAFLAAHGTGTRTGDAVEIAAAAEVF------
TENS               LTMPSSAAQTALIRQTYRKAGLDPVRDRPQFFECHGTGTKAGDPVEARAISDAFLPPSHR
                   :. *    *   :   *   .  *      :. .*.*** .**  *       :
```

```
                   KS                                                        KS
StPKS1             ------KRDSELLIGSVKGNVGHTEITSFLTSFSKVISMFDTNRIPPQANFKEPNPAIHW
SlPKS1             ------KRDSELLIGSVKGNVGHTEITSFLTSFSKVISMFDTNRIPPQANYKNANPAIHW
mFASSusScrofa      ----CATRREPLLIGSTKSNMGHPEPASGVAALIKVLLSLEHGVWAPNLHYHTPNPEIPA
mFASrat            ----CAFRQSPLLIGSTKSNMGHPEPASGLAALTKVLLSLENGVWAPNLHFHNPNPEIPA
MFSQTKS            --GEKRLPDRPLWIGSLKTNIGHSEATSGLASVIQAALALEKGLIPPNINFKEPNEKLSQ
TENS               TNGAATTVDAPLYVGSIKTVVGHLEGCAGLAGLVKVLLSLKHGIIPPNLWFDKLNPEIAR
                              * :** *  :** *  : ::.. :.   :. .   *:  :.  *  :
                   KS                                                KS
StPKS1             EE-YNMRTPTQIEGFTTRNPSGKRLASI--------NASGLLGANGHVIAESPPPKAAKP
SlPKS1             EE-YNMRTTTEIEEFTTRNPSGKRLASMYVVLSLHPNASGLLGANGHVIAESPPPKAAKP
mFASSusScrofa      LQDGRLQVVDRPL------PIRGGNVG--------INSFGFGGSNVHVILQPNSRPAPPP
mFASrat            LLDGRLQVVDRPL------PVRGGIVG--------INSFGFGGANVHVILQPNTQQAPAP
MFSQTKS            VS-SAVKVPSTLEKWPLGS--RVRRAS--------VNNFGYGGANAHVILESGLTGSTQL
TENS               YY-GPLQIPTKAIPWPKLAPGTPLRAS--------VNSFGFGGTNAHAIIERYDASQSYC
                        ::                  ..         *  *  *:* *.* :

StPKS1             SALPTG----------------------M--PVLLMAA-GL-SPRSTTAIAADLSKL--
SlPKS1             SPLPSG----------------------M--PVLLMAA-GL-SPRSTTVIAADLSKL--
mFASSusScrofa      AQHA-------------A-----------L--PRLL-----QASGRTLEAVQTLLEQG--
mFASrat            APHA-------------A-----------L--PHLL-----HASGRTMEA----------
MFSQTKS            ANGNGHYETNGTTNGHKGANGTTNGHK------GANGTTNGHNDYEPLESFVISLSAKEE
TENS               SQ----WRRNMTEEK--TIARTQNNESIEIPVPLVLTAKTGRALWRTVDAYAQHLRQH--
                   :                                            .

StPKS1             -------------AS-----EIPDELPILSNIFGRRARQLTWRAAAI-------------
SlPKS1             -------------AS-----EISNELPSLSNIYGRRARQLTWRAAAI-------------
mFASSusScrofa      -------------LRHSR---------DLAFVGMLN----EIAAVSPVAMPFRGYAVLGG
mFASrat            ------------------------------------------------------------
MFSQTKS            AGTRSMMTNLGEYLRKNHVDDETKHFKSIAYTLGSHRSTFKWTAAKPITSLEELLAAAGG
TENS               --PKLRVTNLSQFMHSRRS---------------THRVRASFSGASREELVENMAKFVQA

                                                       AT                             AT
StPKS1             -------STSDGPFVFPAPRFVPRGTPQLVFVFSGQGPQHIEMGRQLFKYYPVFRDSILK
SlPKS1             -------STSDGPFAFPAPRFVPRGTPQLVFVFSGQGPQHIEMGRQLFKYYPTFRDSILR
mFASSusScrofa      ------------EAGSQEVQQVPGSKRPVWFICSGMGAQWQGMGLSLMRL-DRFRDSILR
mFASrat            ------------------------------------------------------------
MFSQTKS            GQFQASR---------------ALERTRLGFVFTGQGAQWFAMGRELINTYPVFRKSLDR
TENS               HAADAKSPASQNRIGYSPLHIDPKEAPGILGVFTGQGAQWPA------------------

                   AT                                                        AT
StPKS1             MDKVHVELTGKSIVKDLGFFGETRSSTALPDVWPVGLTVPSIAMIQMALVDLLAAFGIRP
SlPKS1             MDKVHVELTGKSIVTDLGFFGETRSSTALPDVWPVGLTVPSIAMIQMALVDLLAAFGIRP
mFASSusScrofa      SDQALKPLGLRV-----SDLL---LSTDEAVLDDIVSSFVSLTSIQIALIDLLTSLGLQP
mFASrat            -------------------------------------VFVSLTAIQIALIDLLTSMGLKP
MFSQTKS            ANGYLKEFGCEW-----SILDELSRDAETSNVNDMTLSPPLCTAVQISLVRLLESWGIVP
TENS               ------------------MA--------------EISQPLCTAVQLALVNVLLASGVHF
                                                             : :*::*: :* : *:
                   AT  **************                                        AT
StPKS1             NLVFGHSAGEAAMSYTSGALPQELAMEIAVRRSQAMS----IVEGSGGMAAVSCAPSVAR
SlPKS1             NLVFGHSAGEAAMSYTSGALPQELAMEIAIRRSQAMS----IVEGSGGMAAVSCAPSVAH
mFASSusScrofa      DGIIGHSLGEVACGYADGCLTQEEAVLSSYWRGYCIK---EANVLPGAMAAVGLSWEECK
mFASrat            DGIIGHSLGEVACGYADGCLSQREAVLAAYWRGQCIK---DANLPAGSMAAVGLSWEECK
MFSQTKS            TAVTGHSSGEIAAAYAAGALDFRSAMAVTYFRGEVGLACQDKIVGKGGMIAVGLGPEEAE
TENS               DAVVGHSSGEIAATYASGIINLEAAMQIAYYRGLYAKLARGETDAAGGMMAAGLSMNDAV
                     : *** ** *  *: *  :  . *:  :  *.             *.* *.. . . .
                   AT                                                        AT
StPKS1             EIVQEVLDEAGPDSGVLEIGCFNAPEAFTISGTHALLDKAVAIASGRGLFARKIKAR-VP
SlPKS1             EIVQEVLQEAGPDGGALEIGCFNAPEAFTISGTHALLDKAVAIASGRGLFARKIKAR-VP
mFASSusScrofa      QRCP----------PGIVPACHNSKDTVTISGPQAAMSEFLQQLKREDVFVKEVRTGGIA
mFASrat            QRCP----------PGVVPACHNSEDTVTISGPQAAVNEFVEQLKQEGVFAKEVRTGGLA
MFSQTKS            DRI------ARVQSGKIVIACINSQSSVTVSGDLAGIVELEEGLKAEGVFARRVKVQ-AA
TENS               KLCR-----LPEFEGRIHVAASNAPQSVTLSGDKEAIKAAKAKLDADGVFARELKVD-TA
                   .               :  .. *: .:.*:**    :       .  .:*.:.::.
```

```
                   AT                                                        AT
StPKS1             GHCTLMEPCKERYVEQMEVAFSR--YPGA----HV----PVVPTFSTQTGARWESEFTPE
SlPKS1             GHCTLMEPCKESYVEQMEIAFSR--YPGA----HV----PVVPTYSTQTGARWESEFTPE
mFASSusScrofa      FHSYFMESIAPTLLRQLRKVILDPKPR------SKRWLSTSIPE--AQWQGSLARTFSAE
mFASrat            FHSYFMEGIAPTLLQALKKVIREPRPR------SARWLSTSIPE--AQWQSSLARTSSAE
MFSQTKS            YHSHHMQVIANGYLTSLKDIL----KPGKKFG-EIIYSSPTTGKRET----SAKLMASAQ
TENS               YHSHHMLPCAEPYLKALLACDIQVSAPTTTPGRKCMWSSSVRGDAELLRHDRNLDSLKGP
                    *.  *       :  :                                        .
                   AT                                                        AT
StPKS1             YMWNNGRVPVQFEQTVTAVVQ-----------EMPEAIFVEIGPHPALSSYISGMGAKPD
SlPKS1             YMWNNGRVPVRFEQTVTAVVE-----------EMPEAIFVEIGPHPALSSYISGMGAKPD
mFASSusScrofa      YSVNNLVSPVLFQEALQHVPA--------------HAVVVEIAPHALLQAVLKRSLESSC
mFASrat            YNVNNLVSPVLFQEALWHVPE--------------HAVVLEIAPHALLQAVLKRGVKPSC
MFSQTKS            HWVNNMLSPVRFAESFQNMCFPTQKVSRSGELEQDVDIILEVGPHGMLQGPIQQMMSLPR
TENS               YWVANMVQTVLFSRAVQSTIWH----------GGPFDLAVEVGPHPALKGPTEQTLKAVY
                   :    *    * * .:.                     : :*:.**  *..  .
                   AT                                   AT
StPKS1             -------KVVCPMRRVKNVTGFNEIFELLTAVGNL-STLGVN----------------TI
SlPKS1             -------KVLCPMRRVKNVTGFNEVYELLSAIGNL-STLGVN----------------TI
mFASSusScrofa      ------TIIPLMK------KDHRDNLEFFLS------NVGR--LHLAGVSVNPNGLFPPV
mFASrat            ------TIIPLMK------RDHKDNLEFFLT------NLGK--VHLTGIDINPNALFPPV
MFSQTKS            FESARMPYLSCLL------RGQSAVYTMQSLAAGL---MG------WGYRV---DMA-AV
TENS               --GSAPLYTGVLS------RGANDAVAFSTAIGNIWSHLGPAFVDITGYQS---IFSSTC
                                       .      :          :*
                                                                 DH          DH
StPKS1             NFHAVNATDCLEISKPIPAYPFAPKTMPFYSESS------------ELAVKMK--RSRKG
SlPKS1             NFHAVNATDSLEISKPIPAYPFAPKSMPFYSESS------------ELAVKMK--RTRKG
mFASSusScrofa      EFPAP---RGTPLI--SPHXKWDHSQA-WDVPSA-ADFPSGSSCSSVAV-----------
mFASrat            EFPVP---RGTPLI--SPHIKWDHSQT-WDIPVA-EDFPNGSSSSSATV-----------
MFSQTKS            NFPQG--THGARILHDLPSYPWNHDNSHWWEPRLNKAHRQRVHPPHDLLGSLIPGRDLRE
TENS               EGHGG--GSAAPFISDLPLYPWDHDEEYWRESRISRRHRTGKDESHELLGRRTPDDNERE
                   :           :    *   :  .   :
                   DH                 ***************                        DH
StPKS1             PLNYDTLAVNALTHPDLAEHVIKGEPILPATGFFEMIFEEGARTIWDIELR---------
SlPKS1             PLNYDTLAVNALTHPDLAEHVIKGEPILPATGFFEMIFEEGARTIWNIELR---------
mFASSusScrofa      -YKF--DVSPESPDHYLVDHCIDGRVLFPGTGYLWLTWKTLARALS----QNLEETPVVF
mFASrat            -YNI--DASSESSDHYLVDHCIDGRVLFPGTGYLYLVWKTLARSLS----LSLEETPVVF
MFSQTKS            PTWR--HFIRVQDIPWIRDHVVQSQLVYPGAGFICMAIEAMVQLHDLKDSQSKKIAGYRL
TENS               IRWR--NLLKVSELPWTQGHRVLGEVLLPGAAYISMAIEAGRRLAL---DQGREARLLEV
                                      * : .. : *.:.:: :  :   :
                   DH                                                        DH
StPKS1             --------SLLPLLPEK---VLNVNV--------------KSDGHAWSIVSSSGGRNPRL
SlPKS1             --------SLLPLLPEK---VLNVNV--------------KSDGHAWSIVSSSGGRNPRL
mFASSusScrofa      EDVTLHQATILPKTGTV---SLEVRL--------------LEASHAFEVSDSNGSLIA--
mFASrat            ENVTFHQATILPRTGTV---PLEVRL--------------LEASHAFEVSDS-GNLIV--
MFSQTKS            ADVDILRAMLIPDTSEGLEAHISLRPCSTK-------LLLTNEWYDFCVSSVGEDDKFVD
TENS               SDVDILRPVVVADNKEGTETLFTVRLLDEYASTGKKSDELITASFSFYIYNSPASTSIVH
                            ::          : :.                   . : : .   .
                   DH                                                        DH
StPKS1             HATGFMT------TEVMDKDAGPIDLAAIR----ARTTPADISNLYAILNNT-AAFGPLY
SlPKS1             HATGFMT------TEVMDKDAGPIDLAAIR----ARTTPADISNLYAILNNT-AAFGPLY
mFASSusScrofa      --SGKVYQWESPDPKLFDTRAAVD---PADS---TAEFRLSQGDVYKDLRLRGYDYGPFF
mFASrat            --SGKVYQWEDPDSKLFDHPEVPI---PAES---ESVSRLTQGEVYKELRLRGYDYGPHF
MFSQTKS            HCRGRIA-VEFNTSSLSDAPKTTSR---ERSRGAGLTRSVDPSNLYSFLRAQGIYHGSIF
TENS               TCEGRIA-VQLGAKLGSEAGANSMPQLPHREPSISNLQQLDCEKLYSVFETIGLEYSGAF
                      * :           :                         .:*  :.     ..  :
                   DH                                       *   DH
StPKS1             RRIEACYEG--DHEILYQVR--GNAPELTAHYNYVFHPSLLDSCIHGLLHPVFTGNADK-
SlPKS1             RRIEACYEG--DHEILYQVR--GNAPELTAHYNYVFHPSLLDSCIHGLLHPVFTGNADK-
mFASSusScrofa      QLVLESDLEGNRGRLQW----NDSWVS------------FLDAMLHMSI-----LAPGQ-
mFASrat            QGVYEATLEGEQGKLLW----KDNWVT------------FMDTMLQISI-----LGFSK-
MFSQTKS            QNLKTISSRKNYSESSFVVADTASVMPDGFQSAHVVHPTTLDSIFQGAYTALPSAGLDQ-
TENS               RRIVSSSRC--LGH----ATATASWPTTDLNDCYLIHPAILDVAFQTIFVARAHPDSGQL
                   : :          .         .                :*  ::           .:
```

```
                        CMeT                                                   CMeT
StPKS1             SVFYLPSHIGRVTLYDRAIEEAVPETLYSY----VVPHDWT----PDSIACDAFIVNERG
SlPKS1             SVFYLPSHIGRVILYDRAVEEAVPETLYSY----VVPHDWT----PDSIACDAFIVNERG
mFASSusScrofa      LGLYLPTRFTSIRIDPVTHRQKL-YTLQDTTQAADVVVDRNL---NTVVAGGALFL----
mFASrat            QSLQLPTRVTAIYIDPATHLQKV-YMLEGDTQVADVTTSRCL---GVTVSGGVYIS----
MFSQTKS            KTAMIPRSIQEIYLSSALTSEVG-QCLVSD--------TSLIRYDGQSFTVNVGISSKAD
TENS               SSALLPSRIERVRVVPSLA--MG-SKLQNNENFNAAIDSWALNQTASSLTGNINVY--DA
                       :*  .  : :            * .                   .:  .  .
                   CMeT                                                    CMeT
StPKS1             ERLVTLI-----------------------DCVLSKHWTGAVP------TR---------
SlPKS1             ERLVTLI-----------------------DCVLSKHWTGAVP------TR---------
mFASSusScrofa      ----------GAH--SSVAPRRPQE--------------------HLKPILEKFCFTPHV
mFASrat            ----------RLQ--TTATSRRQQE--------------------QLVPTLEKFVFTPHV
MFSQTKS            SECTPVLEIKGLRNQSVGQMAPQQGDSGNNDLCFKLEWALDISSVKQERLKEKFGFPLDP
TENS               ESGRALIQVEGFEVRAVGEP----DASKDRLLFYETVWGRDISIMGLSDPIRD--ETSDA

                   CMeT                                                    CMeT
StPKS1             ----------PDTSYEYIYQPLGLPAAELVKSEAQQQDYA---FLDAIVAHADEKVAPPS
SlPKS1             ----------PDTSYEYIYQPLGLPAAELSTSKAQQQDYA---FLDALVAHAGEKEAPPS
mFASSusScrofa      ESGCLAGNTALQEELQLCRG----LAQALQTKVAQQG----LKM---VVPGLDGAQAPRE
mFASrat            EPECLSESAILQKELQLCKG----LAKALQTKATQQG----LKM---TVPGLED------
MFSQTKS            AEADIIM-GLRQACLYYIRQ----ALTSLTPSARDQLDWHQKRFYDWMMLQMHLAEEDRL
TENS               MVHNLSE-AIERVSLFYVRQ----LMGELSTADRRQANWYHTRMLAAFDYHLAKVHEETH
                                               *      *       :
                   CMeT                                                    CMeT
StPKS1             ANGH--------------ANGHANGSANGSAVGTVGEDRKVFEEIVQSIASDELELKASS
SlPKS1             ANGH--------------ANGHANGHANGSAVGTVGEDRKVFEEIVQSIASDELELKPSA
mFASSusScrofa      APQQ---------SLPRLLAAACQLQLNGNLQLELGQVL------AQERP---LLCDDPL
mFASrat            LPQH---------GLPRLLAAACQLQLNGNLQLELGEVL------ARERL---LLPEDPL
MFSQTKS            APNSSAWLQCTSSDEQKLLENVRAASVNGQMVVHVGESI--LAILRHEIAPLELMLQDKL
TENS               LHLRPEWLADDWAVIQ-TIDEAYPDAVELQMLHAVGQNV--ADVIRGKKHLLEVLRVDNL
                                              : .    :*:          .     :
                   CMeT                              **********            CMeT
StPKS1             ILGLFSASLDAPVAAVRQILDHAA---KSGKQVVRILDIGDATASLYKQI-NAFASEYPS
SlPKS1             LLSLFSASLDAPVAAVRQILDHTA---KSGKQVVRILDIGDATASLYKQI-TALASEYPS
mFASSusScrofa      LSGLLDAP------ALKACVDTA--LENMASPKMKVVEVLAGDGQLYSRI-PALLNTQPV
mFASrat            ISGLLNSQ------ALKACIDTA--LENLSTLKMKVVEVLAGEGHLYSHI-SALLNTQPM
MFSQTKS            LYRYYTDAIK--WDRSYQQIDQLVKLHAHKCPSAKIIEIGAGTGGCTRAVLDALSTHGAA
TENS               LDRLYTEDKG--MHMANLFLANALEEITFKFPRCKILEIGAGTGATTWAALSAIG----E
                   :                  :              ::::   . .         *:
```

Strobilurin PKS CMeT domain shows closer similarity to non-functional CMeT domains of mFAS than the functional domains of TENS and SQTKS. Red highlighted text indicates functional SAM-binding residues in TENS and SQTKS.

```
                   CMeT                                                    CMeT
StPKS1             LRVDYTACGHEHATLDLRLA---SYN---------VD-NVSKQAGLSPSTYDVIIETHTL
SlPKS1             LRVDYTACGHEHATLDLRLA---SFN---------VD-NVSKQTGLSPSTYDVVIETHTL
mFASSusScrofa      MDLDYTATDRNPQALEAAQAKLEQLHVT------QGQWDPANPAPGSLGKADLLVCNCAL
mFASrat            LQLEYTATDRHPQALKDVQTKLQQHDVA------QGQWDPSGPAPTNLGALDLVVCNCAL
MFSQTKS            RCAQYDFTDVSSGFFEAAQQKFTAFADVIRFQKLDIEKDI-ETQGFECGSYDLVIASQVL
TENS               AFDTYTYTDLSVGFFENAVERFSAFRHRMVFRALDIEKDP-ASQSFDLNSYDIIIATNVL
                       *   .     :.                    : :       . .  *:::  . .*
                   CMeT                                                    CMeT
StPKS1             GFAAELDRSLEYLHGLLLPGGFLVALEANGSAQASGGKWIDQVFSP-QGRWSGLRS--GK
SlPKS1             GFAAELERSLEYLNGLLVPGGFLVALEANGSAQASGGAWIDHVFPP-QGRWSGLRS--GK
mFASSusScrofa      ATLGDPAVAVGNMAATLKEGGFLLLHTLLAGHPLG----------EMVGFLTSPEQ---G
mFASrat            ATLGDPALALDNMVAALKDGGFLLMHTVLKGHALG----------ETLACLPSEVQ---P
MFSQTKS            HATGKIEDTMANVRRLLKPGGKLLLVETTR-DEMDL----QLVFGLLPGWWLSSEEER-K
TENS               HATRNLGVTLGNVRALLKPGGYLLLNEKTGPESLRA----TFNFGGLEGWWLAEEKER-Q
                       .   ::  :   *  **  *:                        .   .  .
                   CMeT                   CMet                      ΨKR     ΨKR
StPKS1             QHHRLSQSEWSGQLQKAKFQVVDGAQD-AENTLF--------LTLLAQKHSLSTVSASS-
SlPKS1             QYHRLSESDWNGQLQKAKFQVVDGAQD-VGNNLF--------LTLLAQKHSLSVVSASS-
mFASSusScrofa      GRHLLSQDQWESLFAGASLHLVAL-----------------------KRSFYGSVLFL--
mFASrat            GPSFLSQEEWESLFSRKALHLVGL-----------------------KKSFYGTALFL--
MFSQTKS            MSPSLSTSSWEKVLKKTGFNGLDVELRDCDSDQF-----------------YSFSVIMAT
TENS               LSPLMSPDGWDAQLQKASFSGVDHIVHDVQEDQQDKQQNSMIMSQAVDDTFYARLSPLSE
```

```
                         :*  . *.  :    :  :                              .
                         ΨKR                                                         ΨKR
StPKS1                   AASSAKVEEPAVFSF---------------------------------------------
SlPKS1                   TESSAKVEEPVVFNF---------------------------------------------
mFASSusScrofa            CRQQTPQDSPVFLSVE--------------DTSFRWVDSLKDILADASSRPVWLMAVGCS
mFASrat                  SPQDKPIFLPVE--------------DTSFQWVDSLKSILATSSSQPVWLTAMNCP
MFSQTKS                  ASPTVPMNPVDFII-----------LHGKSSIPDQWMNDLRTATSPFT-----------K
TENS                     MANLLPMNEPLLIIGGQTTATLKMIKEIQKLLPRQWRHKVRLIASVD-------------
                                :   .:
                         ΨKR                                       ΨKR
StPKS1                   --------DH-------S--RV---LDLQKTVL---------ASMS--SGASNTKLWIES
SlPKS1                   --------EH-------S--RV---LEVQKAVL---------ASMS--SGASNSKFWIES
mFASSusScrofa            TSGVVGMVNCLRKEPGGHRIRCVLVSNL------------------------------SS
mFASrat                  TSGVVGLVNCLRKEPGGHRIRCILLSNL------------------------------SS
MFSQTKS                  SDPVVGHINNA--DPTGK--FCIFLEDPEEDIL-FHPDEKSYASIKRVITQCKGLLWISR
TENS                     ------HVEAEG-LPAHS--DVICLQELDRGLFTTAMTSKCLDALKTLFINTRNLLWVTN
                                 :                 :
                          ER                                                         ER
StPKS1                   TTGTFD----GAVATGFARSLMRELVAVD---VRLVLFDPAWKAESRIPAIRQLSTLPSL
SlPKS1                   TIGTFD----GAVATGFARSLMRELVAVD---VRLVLFDPVWTAESRIAAIRQLSTLPSL
mFASSusScrofa            TSPAPEMHPSSSEL---QKVLQGDLVMNV-------YRDGAWGAFRHF----------PL
mFASrat                  TSHVPKLDPGSSEL---QKVLESDLVMNV-------YRDGAWGAFRHF----------QL
MFSQTKS                  GGSMHGTLPTSSLKTGLLRTLRLEYAEKRFISLDLNPARAPWAHESISTIREVLRGALAQ
TENS                     AQNSSSMTPRASMFRGITRVLDGEVPHIRTQVLGIEPRETPSATAR-----TLLEAFLRL
                                  .:       :  *   :
                         ER                                                          ER
StPKS1                   ESE-----------------------------IVLDASGVVMVPRLRSYAPRAPDSLDT-
SlPKS1                   ESE-----------------------------IALDASGVIQVPRLRSYAPRAPDTLDD-
mFASSusScrofa            EQDRPEKQTEHAFVNVLSRGDLSSIRWVCSPLHYAL--------PASCQ-----------
mFASrat                  EQDKPEEQTAHAFVNVLTRGDLASIRWVSSPLKHMQ-------PPSSSG-----------
MFSQTKS                  TAEIPIRDS--E---------------------FAENDGQLYVPRISSDIARNEALSSNS
TENS                     RSDDGRHAG--NVDEDGADGSSQQVLWLHEPEAELLSNGTMMVPRVKARKSLNDTYLAS-
                           :                                                .
                         ER                                                          ER
StPKS1                   ----------------------------T-------------KYW------VVDETKTVV
SlPKS1                   ----------------------------S-------------KYW------VVEEAKTVV
mFASSusScrofa            -----------------------------------------DRLC--SVYYTSLNFRDVM
mFASrat                  -----------------------------------------AQLC--TVYYASLNFRDIM
MFSQTKS                  HSPAQTEPFHQPGKLLQMGIKTPGLIDTLQFSKTDAPDHLPADYIEIEPKAFGLNFRDVM
TENS                     --------------------------------TRAISTTVDARCVSVQA---------VA
                                                                                   :
                         ER                                                          ER
StPKS1                   QPAQPL---------PGPHQVLVKISSLSEAEGGLRGIVGTV---------ARSGSSQWP
SlPKS1                   QPALPL---------PGPHQVLVKISSLSDVEGGLRGIVGTI---------ARSGSSQWQ
mFASSusScrofa            LATGKLSPDSIPGKWLTRDCMLGMEF-SGRDASGR-RVMGMVPAEGLATSVLLLQHATW-
mFASrat                  LATGKL-------KWASRDCMLGMEF-SGRDKCGR-RVMGLVPAEGLATSVLLSPDFLW-
MFSQTKS                  VAMGQLEESIMG--FECAGIV---------RRVGP-SSAGHNIKVGDRVCALLGGQWTNT
TENS                     GPAKMLLRPVED--FAGEHAISNQTS-DSKVHIQV-ESTLHIPEALDGTCLYLVCGWTRT
                              *              :
                         ER                                                      ****ER
StPKS1                   VGAHVVTVAPSA--LS---------------------NFTLVHEGQLAQAPQTADE----
SlPKS1                   VGARVVTVAPSA--LS---------------------NFTLVHEGQLAQVPETVDE----
mFASSusScrofa            ---------EVPSTWTLEEAASVPIVYTT-------AYYSLVVRGRM-QPGESVLIHSGS
mFASrat                  ---------DVPSSWTLEEAASVPVVYTT-------AYYSLVVRGRI-QHGETVLIHSGS
MFSQTKS                  VRVHWHAVAPIPQAMGWETAASIPIVFVT-------AYISLVKIAKL-QAKETVLIHAAS
TENS                     AETSVPVIALSANNASMVAVESKAVAMIDEVDVKPETLLRVFQHMAM-QALDSA---VKR
                                                                    :.     :  *   ::.
                         ER*****                                                     ER
StPKS1                   HSTAKVALL-------LVFAALGLRLDSRPLQSLQQIKVVVIHTGTVA------------
SlPKS1                   HSAANVALL-------LVFAALGLRLDSRPLKSLQQIRVVVIHTGKVA------------
mFASSusScrofa            GGVGQAAIAIALSRGCRVFTTVGSAEKRA----YLQARFPQLDETCFANSRDTSFEQ---
mFASrat                  GGVGQAAISIALSLGCRVFTTVGSAEKRA----YLQARFPQLDDTSFANSRDTSFEQ---
MFSQTKS                  GGVGQAAIILAKYAGAEIFATVGTEEKRE----LLIKEYKIPDDHIFSSRNA-LFAK---
TENS                     HGQGQSTAL--IYGADEELA--------K----LTSERFAVRESKVYFASSR-TFAPGDW
                          . .: :           ::                   .    .
```

NB - mFAS and SQTKS have active ER domains, the others do not. Red text indicates functional cofactor binding site in mFAS and SQTKS.

```
              ER                                                      ER
StPKS1        ---------------------------------------SSLARLLEYLGVKPVLV----
SlPKS1        ---------------------------------------SSVARVLEHLGVKPTLV----
mFASSusScrofa ---------HVLRHTAGKGVDLVLNSLAEEKLQASVRCLAQHGRFLE-IGKFDLSNNHAL
mFASrat       ---------HVLLHTGGKGVDLVLNSLAEEKLQASVRCLAQHGRFLE-IGKFDLSNNHPL
MFSQTKS       ---------SIRQRTNGKGVDVVLNCLAGGLLQESFDCLADFGRFIE-IGKRDIELNHCL
TENS          LKVQPLLSKFALSQMIPADVEVFIDCLGDTE---SFDACRTLQSCLSTTRTVQHRLDACL
                                                           :.

              ER                                                      ER
StPKS1        ---------------------------APSLPLLLPRLSPGDVIIGGLSAAFARTVPRIN
SlPKS1        ---------------------------APSLPLLLPRLSPGDVIIGGLSAASARTVPRIT
mFASSusScrofa GMAVFLKNVTFHGILLDSLFEEGGATWQEVSELLKAGIQE-----GVVQPLKCTVFPRTK
mFASrat       GMAIFLKNVTFHGILLDALFEGANDSWREVAELLKAGIRD-----GVVKPLKCTVFPKAQ
MFSQTKS       NMGMFARSATFTAVDLIAIGRDRSYMVAEALPKVMALLQQ--KAVRPVTPISIYKIGDIE
TENS          LSQMS-------------------------------------RCSPDALVDAYSYAKTQ

              ER                                                      ER
StPKS1        GVSVFNWEDPEQGALAAVAQNPWLVGTTVDAHLARALPQVSVEGSSLTPDQLLPSDFSVS
SlPKS1        GVTFFNWEDPEQGALAAVAQNPWLVGSTIDAHLTPALPEVSVEGASLTPAQLLPSDFDVS
mFASSusScrofa VEAAFR------YMAQGKH-IGKVVIQVREEEQGPAPRGLP------PI------ALTGL
mFASrat       VEDAFR------YMAQGKH-IGKVLVQVREEEPEAMLPGAQ------PT------LISAI
MFSQTKS       T--AFR------LMQAGKH-MGKIVITAPEDAMVPVSNQIPVARGVNHMLTFFVQVVTQP
TENS          SNAEFSWNGYVKTFTAAEL-AGKLSHSLIHSVY---------MTNWQKKDSILVTVPPLQ
                  *           .      :     .

              ER KR     ***********                                   KR
StPKS1        QSLALADDKTYLVLGGIGSLGLQIAIWMYQKGARHIVLTSRTGVSRLAGTKNRSLRGAVE
SlPKS1        QSLALADDKFYLILGGIGSLGLQVAIWMYQKGARHIVLTSRTGVSRLAGTKNRSLRGAVE
mFASSusScrofa SKTFCPPHKSYVITGGLGGFGLQLAQWLRLRGAQKLVLTSRSGIRTGYQ--ARQVREWR-
mFASrat       SKTFCPEHKSYIITGGLGGFGLELARWLVLRGAQRLVLTSRSGIRTGYQ--AKHVREWR-
MFSQTKS       PKLQLRSDASYLIVGGLGGIGRSLCKNFVENGARSLVLLSRNANVSRQS--GEFLDELR-
TENS          TRGLFKSDRTYLMVGAAGGLGTSICRWMVRNGARHVVVTSRNPKA---D--PEMLNEAE-
                     .  *:: *. *.:* .:.  :  .**: :*: **.          . :

              KR                                                      KR
StPKS1        YLKTLPDLELRLEPCDASSEESLSKLI---SSLDRPLAGAMLTAAVMADGLFLKQSADTY
SlPKS1        YLKTLPDLELRLEPCDASSLESLSKLI---SSLDRPLAGAMFTAAVMSDGLFLKQNSDTY
mFASSusScrofa ----RQGVQVLVSTSNASSLDGARSLI-TEATQLGPVGGVFNLAMVLRDAVLENQTPEFF
mFASrat       ----RQGIHVLVSTSNVSSLEGARALI-AEATKLGPVGGVFNLAMVLRDAMLENQTPELF
MFSQTKS       ----STGCVVSVVDCDISNKTQVESTMLRLKEEKLPIRGIVHAGMVLQDSVFEHMTLEDY
TENS          ----RYGAAVQVVPMDACSKDSVQTVVDMIRATMPPIAGVCNAAMVLRDKLFLDMNVDHM
                    .  : :   : ..       :        *: *    . *: * :: . . :

              KR                                                      KR
StPKS1        PIPFKPKTDAYFAFEKVVDI--KKLDFLLAVSSVA-GFGAAGQTNYASANTGIEYLTARY
SlPKS1        PIPFKPKTDAYFAFEKVVDI--QKLDFLLAVSSVA-GFGAAGQTNYASANTGIEYLTARY
mFASSusScrofa QDVSKPKYSGTANLDRVTREACPELDYFVIFSSVSCGRGNAGQANYGFANSAMERICEKR
mFASrat       QDVNKPKYNGTLNLDRATREACPELDYFVAFSSVSCGRGNAGQSNYGFANSTMERICEQR
MFSQTKS       NTATRPKVRGSWNLHSALSD--CDLDFFIMLSSLAGVSGSASQANYTAGGAYQDALATYR
TENS          KDVLGPKMQGTEHLDSIFAQ--EPLDFFVLLSSSAAILNNTGQSNYHCANLYMDSLVTNR
                   **  .   :.         **::: .** :   . :.*:**  ..    : :

              KR                                                      KR
StPKS1        PNAWSFVAPGIADSNVGFDLFTSTNSH---------------LEQWESSTMNSYEICLCL
SlPKS1        PNAWSFVAPGIADSNVGFDLFTSTNSH---------------LEQWESSTMNSFEICLCL
mFASSusScrofa RH------DGLPGLAVQWGAIGDVGVVLETMGTNDTVIG-GTLPQRIASCLEVLDLFLSQ
mFASrat       RH------DGLPGLAVQWGAIGDVGIILEAMGTNDTVVG-GTLPQRISSCMEVLDLFLNQ
MFSQTKS       RS------RGLAAVSIDLGMVQSVGYVAETKGVAERLVRMGYSP---ISEMEVLKIVEHA
TENS          RS------RGLAASIIHVGHVCDTGYVARLVDDTKVQMSLGTTRVMSVSETDVHHAFAEA
                       *:    :  . . ...                       *  :  .

              KR                                                      KR
StPKS1        -------------------EDGLLRMANNERISIYVPNLNWDAISQSVSESVLYNHLV--
SlPKS1        -------------------EDGLLRMANKERISIYVPNLNWDAISQSVSESVLYNHLV--
mFASSusScrofa ---PHPV------------------------------------LSSF---VLAEKKAAAP
mFASrat       ---PHAV------------------------------------LSSF---VLVEKKAVAH
MFSQTKS       ITNPPPETS--SGQIITGISTKPG---RHWTESSWLQDARFATLRER---ARDVKEQ-SN
TENS          VRGGQPDSRSGSHNIIMGIEPPTKPLDLTKRKPVWISDPRLGPCLPF---STLENQMMAS
                                                                    :.
```

```
                   KR  KR                       ACP                                  ACP
StPKS1             -------KLDAATDELE-----VEDPYEVLQEI------VLKFVDASEEEFERNVPLTSY
SlPKS1             -------KLDAATDELE-----VEDPYEVLQEI------VLKFVDASEDEFERNVPLTSY
mFASSusScrofa      RDGSSQKD-------------L-------VKAV------AHILGIRDVASINPDSTLVDL
mFASrat            GDGEAQRD-------------L-------VKAV------AHILGIRDLAGINLDSSLADL
MFSQTKS            AQGGGQDKQIGAGQELSMATSLVEAIDVVGRAITAKLATMFLIAAES---IIASKSLSEY
TENS               EQAAAASAVDSLAQQVSEATTDEEAAVAALKGFATKLEGILLLPLGSIGEDSAGRPVTDL
                                                :  .           :    .      .   :  .
                   ACP*******                                                    ACP
StPKS1             GLDSLSAARMSTALKPY-------LAITQIQLLGDLSLDDLVEKMQATKHVAVEE----T
SlPKS1             GLDSLSAARMSTALKPY-------LAITQIQLLGDLSLDDLVEKMQATKHVAVED----T
mFASSusScrofa      GLDSLMGVEVRQILEREHDLVLSMREVRQL-SLRKLQE--LSSKTSTDADPATPTSHEDS
mFASrat            GLDSLMGVEVRQILEREHDLVLPIREVRQL-TLRKLQE--MSSKAGSDTELAAPKSKNDT
MFSQTKS            GVDSLVAVELRNWLAAQLSSDVSVFDVTQSQSLTALAT-----T------VATKSSRIDK
TENS               GIDSLVAVEIRTWFLKQLRVDVPVMKILGGSTVGQLSA--LAAK------LARQDAKKRA
                   *:***  ....:    :            :      :   *       .        *
                   ACP     ACP                        mFAS-TE                mFAS-TE
StPKS1             AV--STAEKPF--------------AWD--AMHQPGQTILKFNIGSGTPLIILHGGAGDT
SlPKS1             PA--STTEKPF--------------AWD--AMHQPGQTILKFNIGSGTPLIILHGGAGDT
mFASSusScrofa      PVRQQ---------------ATLNLSTLLVNPEGPTLTRLNSVQSAERPLFLVHPIEGSI
mFASrat            SL-KQ---------------AQLNLSILLVNPEGPTLTRLNSVQSSERPLF---------
MFSQTKS            SLLVA-------------------------------------------------------
TENS               QLEEPSGNQPVALPSPPPKDKAGGLNKNGKSPKLPEIAQVDTVVERMEPLV---------

                   mFAS-TE    *                                          mFAS-TE
StPKS1             AAFRAIQEQFSTPLWAIQPTPEAPLDTVDTLAQFYFEKIKEARPAGPYRIAGFSASSMVT
SlPKS1             AAFRAIQEQFSTPLWAIQPTPEAPLDTVDTLAQFYFEKIKEARPAGPYRIAGFSASSMVT
mFASSusScrofa      TVFHGLAAKLSIPTYGLQCTGAAPLDSIQSLASYYIECIRQVQPEGPYRIAGYSYGACVA
mFASrat            ------------------------------------------------------------

                   mFAS-TE    UNKNOWN                                    UNKNOWN
StPKS1             LRLAQLLEANEDEIAQLTFVDHFPLFFTSAIHGFTEDHKTFEDLTAYGRKASVALVAECC
SlPKS1             LRLAQLLEANEDEIAQLTFVDHFPLFFTSAIHGFTEDHKTFEDLAAYGKVASVALVAECC
mFASSusScrofa      FEMCSQLQAQQSATPG-----NHSLFLFDGSHTFV---------LAYTQSVRAKMTPGCE
mFASrat            ------------------------------------------------------------
                   UNKNOWN                                               UNKNOWN
StPKS1             RRDTATARRLYGENLVAASNGQ----------------------------PSATNAME-
SlPKS1             RRDSAPARRLYGENLVAASNGL----------------------------PSAANAIE-
mFASSusScrofa      AEAEAKAMYFFVQQFTDMEQGKVLEALIPLQGLEARVAATVDLITQSHAGLDRHALSFAA
mFASrat            ------------------------------------------------------------

                   UNKNOWN                                               UNKNOWN
StPKS1             ----------SWEWIQKTTRMNLKQVV-DFGGGW--------EAWASSDATTRMEAARRR
SlPKS1             ----------SWEWIQKTTRMNLKQVV-DFGGGW--------DAWVAADGKTRMETARRR
mFASSusScrofa      RSFYQKLRAAENYWPQATYHGNVTLLRAKTGGAYGEDLGADYNLSQVCDGKVS-------
mFASrat            ------------------------------------------------------------

                   UNKNOWN                                               UNKNOWN
StPKS1             MVEEIAKVKAPMNVMIANWGIRALINSEWTDLGISRGGREVRTQYYDAGHFDIFEKPDFS
SlPKS1             MVEEIAKVKTPMNVMIANWGIRALINTDWTDLGVSRAEREVRVQYYDSGHFDVFEKPDFS
mFASSusScrofa      ------------------------------------------VHVIEGDHRTLLEGSGL-
mFASrat            ------------------------------------------------------------

                   UNKNOWN                                               UNKNOWN
StPKS1             RNLEFDWVDPHPVHQLATMIHNPAMNDLRALFKILDTKALQVMADTISQNPVVGSEISRQ
SlPKS1             RNLEFDWVDPQPVHQLASMIHNPAMKDLKALFKILDTKALQVMADTISQNPLVGSEISRQ
mFASSusScrofa      -------------ESILSIIHSCL------------------------AEPRVSVREG--
mFASrat            ------------------------------------------------------------

                   UNKNOWN                                               UNKNOWN
StPKS1             RLFEVCKEFVRTQKHSTWTDEEYEHSKALFPTYFETTERISKVHPSIMESPAAAVGALYS
SlPKS1             RLFEVCKEFVRTQSHSTWTDEEYEHSKALFPTYFETTERISKVHPSVMESPAAAVGALYS
mFASSusScrofa      ------------------------------------------------------------
mFASrat            ------------------------------------------------------------
```

```
                   UNKNOWNCMeT                                  ************      CMeT
StPKS1             DDMIDGFYRQNKVFTSMNQEAAKTF-KALVSSPDFGKQRPIRVLEVGAGVGGLTKFLVEA
SlPKS1             DDMIDGFYRQNKVFTSMNQEAAKTF-KSLVSSPDFGKQRPIRVLEVGAGVGGLTKFLVEA
mFASSusScrofa      ------------------------------------------------------------
mFASrat            ------------------------------------------------------------
CurJ               ------LYKDSAVAKVMNTIVEKVIMKAMEKLP---PSRGIRLLEIGAGTGGTTSYILPH

                   CMeT                                                    CMeT
StPKS1             LCDMPNADVEYTVTDLSYTLASSLAESF-SYKNMVAKMYDLSKKPSEQGLQLGHYDVITG
SlPKS1             LCDMPNADVEYTVTDLSYSLASSLAESF-SYKNMVAKMYDLSKKPSEQGLQLGHYDVITG
mFASSusScrofa      ------------------------------------------------------------
mFASrat            ------------------------------------------------------------
CurJ               LN--PN-QTEYIFTDIGALFTSKAQEKFQDYRFLGYQTLDIEVDPSSQGFESHRYDVIIA

                   CMeT                                                    CMeT
StPKS1             LNVIHAVPDLNATLTDLHSLLAPGGRILIVDTDGTARTSNPPRPGAIWNDFIWGSFQGWF
SlPKS1             LNVIHAVPDLNGTLADLHSLLAPGGRILVVDTDGTARTSNPPRPGAIWNDFIWGCFQGWF
mFASSusScrofa      ------------------------------------------------------------
mFASrat            ------------------------------------------------------------
CurJ               ANVLHATTSLKQTLSHVRQLLAPGGILVLYEATTRSR----------WVDLIFGLLEGWW

                   CMeT                          CMeT
StPKS1             GYTDDRT---HCTIDEDEWRKRLTATGYSNVQVCHEDAGTCILFEAEKV
SlPKS1             GYTDDRT---HCTIDEQEWRKRLTATGYSNVQICHEDAGTCILFEAEKV
mFASSusScrofa      --------------------------------------------
mFASrat            --------------------------------------------
CurJ               KFTDYELRPDYPLLNREQWKKVLSETGFTQVVTLPEVEG
```

Supplementary Figure 10. Multiple Alignment of StPKS1 and SlPKS1 with each other and with known polyketide synthases. The coloured bars show Approximate Domain Boundaries. *** = active site / cofactor binding positions; mFAS - mammalian fatty acid synthase; SQTKS - squalestatin tetraketide synthase; TENS - tenellin synthetase polyketide synthase**;** CurJ, Curacin CMeT domain**.** Yellow highlight, 100% conserved; green highlight, similar residues.

```
polyketide synthase [Stereum hirsutum FP-91666 SS1]
Sequence ID: Length: 2927Number of Matches: 1
Related Information
Range 1: 2310 to 2640

Alignment statistics for match #1
Score            Expect  Method                          Identities     Positives      Gaps
300 bits(767)    7e-89   Compositional matrix adjust.    149/331(45%)   203/331(61%)   25/331(7%)

Query  1      DEIAQLTFVDHFPLFFTSAIHGFTEDHKTFEDLTAYGRKASVALVAECCRRDTATARRLY  60
              DEI QLTFVDHFP+ F S +H F    ++FE+L       +V ++++ C RD   ARR Y
Sbjct  2310   DEIVQLTFVDHFPMLFASPLHDFNTTPESFEELGHLAALKTVDMISDSCARDKTAARRAY  2369

Query  61     GENLVAASNGQPSATNAMESWEWIQKTTRMNLKQVVDFGGGWEAWASSDATTRMEAARRR  120
              G  LV+AS   PS   A+ESW +I+K   MNL+Q + F GG+E W+      R E  R R
Sbjct  2370   GLALVSASQNLPSTPTALESWTFIRKIAVMNLRQAIAFSGGFEVWSGLSPAEREERMRVR  2429

Query  121    MVEEIAKV-----KAPMNVMIANWGIRALINSEWTDLGISR--------------GGREV  161
              M+EE+  V        M+  +A+WG+R L+  +W D G+ R                R+
Sbjct  2430   MIEEVRSVGRRLSACRMSAYVADWGLRTLMPDDWNDFGVGRCFGDGRLGDPRAGVDERKA  2489

Query  162    RTQYYDAGHFDIFEKPDFSRNLEFDWVDP------HPVHQLATMIHNPAMNDLRALFKIL  215
              +   Y AGHFDIFE+ DFSR+LE DWVD          ++  +M+H+P  +DL  +F+IL
Sbjct  2490   KVYRYPAGHFDIFERSDFSRSLEVDWVDAAIEAGAESEYEWKSMVHHPLKDDLTTMFRIL  2549

Query  216    DTKALQVMADTISQNPVVGSEISRQRLFEVCKEFVRTQKHSTWTDEEYEHSKALFPTYFE  275
              DT AL+ M ++I QNPVV SEISRQRL+    E++RT+K + WT+EEY   K+++P YFE
Sbjct  2550   DTMALKYMKESIEQNPVVDSEISRQRLYRETLEYLRTRKPAIWTEEEYTRLKSIYPLYFE  2609

Query  276    TTERISKVHPSIMESPAAAVGALYSDDMIDG  306
              TTERI  VH S  +SP AAV ALY+D+MIDG
Sbjct  2610   TTERIGSVHASTFQSPTAAVAALYADNMIDG  2640

ketoacyl-synt-domain-containing protein [Gloeophyllum trabeum ATCC 11539]
Sequence ID: XP_007863729.1Length: 2273Number of Matches: 1
Related Information
Range 1: 1201 to 1300

Alignment statistics for match #1
Score           Expect  Method                        Identities   Positives    Gaps
67.4 bits(163)  8e-09   Compositional matrix adjust.  37/100(37%)  57/100(57%)  1/100(1%)

Query  203    PAMNDLRALFKILDTKALQVMADTISQNPVVGSEISRQRLFEVCKEFVRTQKHSTWTDEE  262
              PA+ D R L++ LD  A+ ++  ++     VG EI R+R  +   E ++ Q  ST  DE
Sbjct  1201   PAIKDSRRLYECLDDLAVTIIRKSLEDPHRVGPEIHRERYMKFAMEALQRQTTSTKVDEA  1260
Query  263    YEHS-KALFPTYFETTERISKVHPSIMESPAAAVGALYSD  301
                +  +A +P YF  T++++K+HP I ES    V ALYSD
Sbjct  1261   VLNELRASYPNYFLITDKVAKIHPLIFESHKVTVEALYSD  1300
```

Supplementary Figure 11. Alignments of the unknown domain of stpks1 with a polyketide synthase from *Stereum hirsutum*, and a ketoacyl-synthase-domain-containing protein from *Gloeophyllum trabeum*, generated using blastp to search the NCBI database.[3]

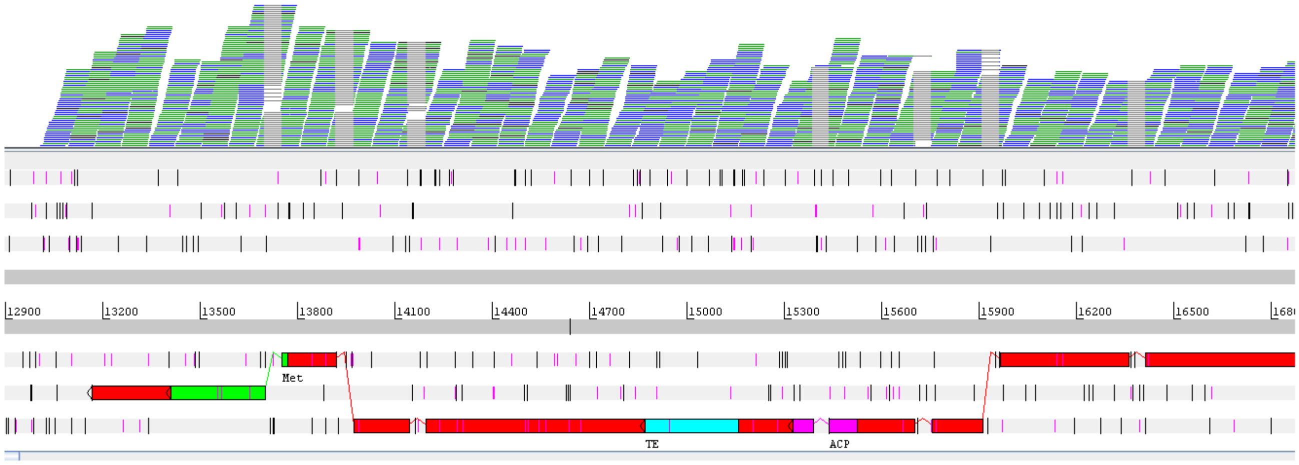

Supplementary Figure 12. Transcription analysis of 3' end of *stpks1*. Grey vertical bars in the mapped reads indicate intron positions. *Stpks1* is in red. ACP domain = purple. Hydrolase/TE domain = blue. MeT domain = green.

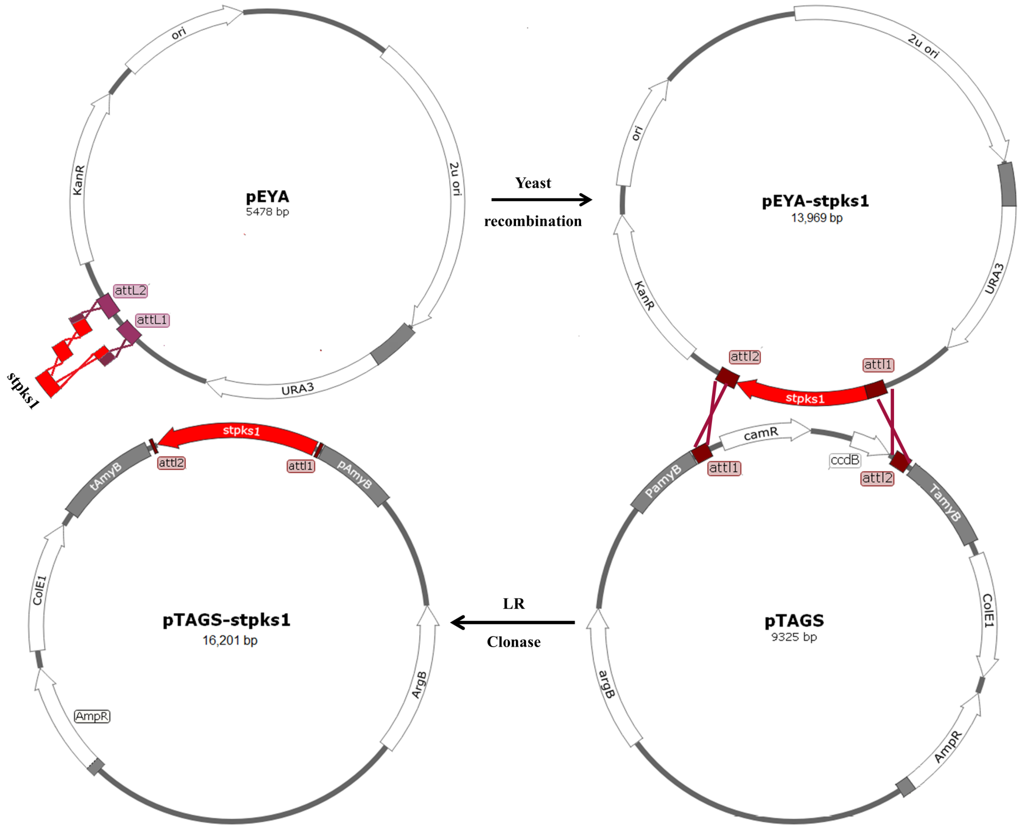

Supplementary Figure 13. The *stpks1* gene was reassembled from cDNA in a yeast assembly vector, pEYA, and transferred into an expression vector, pTAGS, by Gateway® recombination. Sizes of the plasmids and DNA fragments are not to scale.

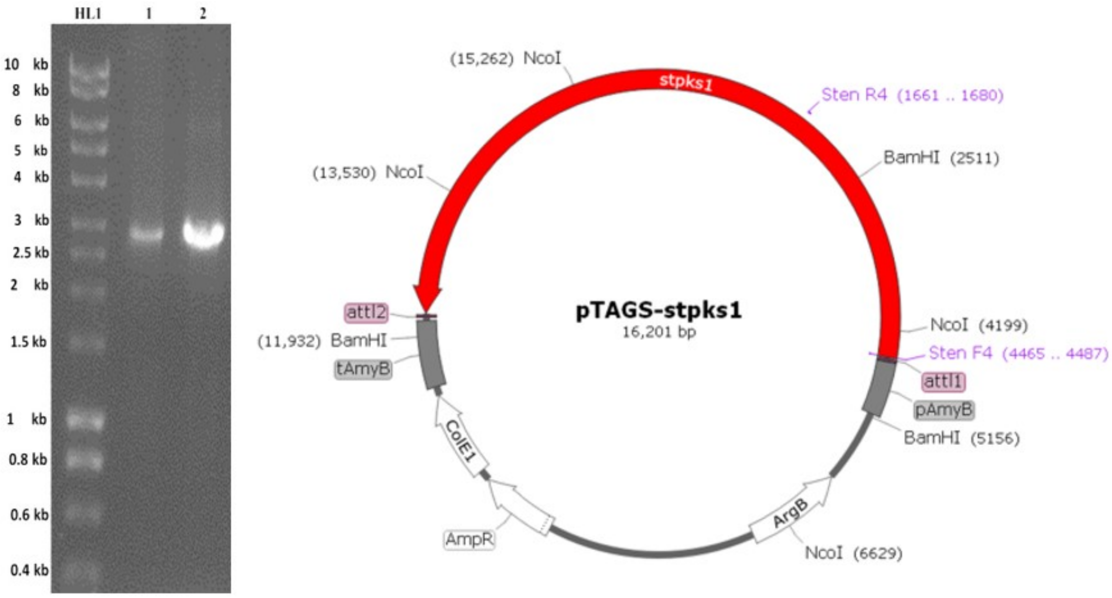

Supplementary Figure 14. Screening of pTAGS-*stpks1*. **Left**, Colony PCR on two separate *E. coli* colonies with primers *stpks* F4 and *stpks* R4; **Middle**, Map of pTAGS-*stpks1* showing *Bam*HI and *Nco*I sites.

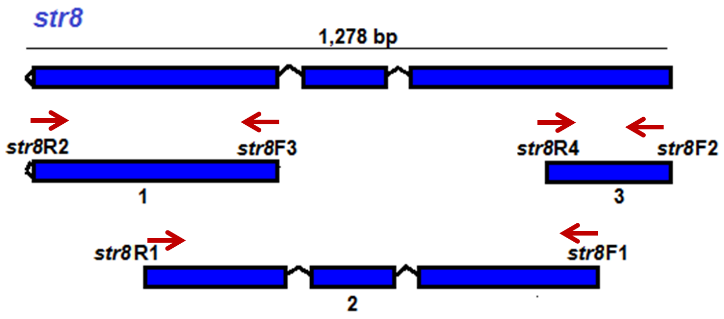

Supplementary Figure 15. Amplification of *str8* using various primers: **Red arrows**, Primer position.

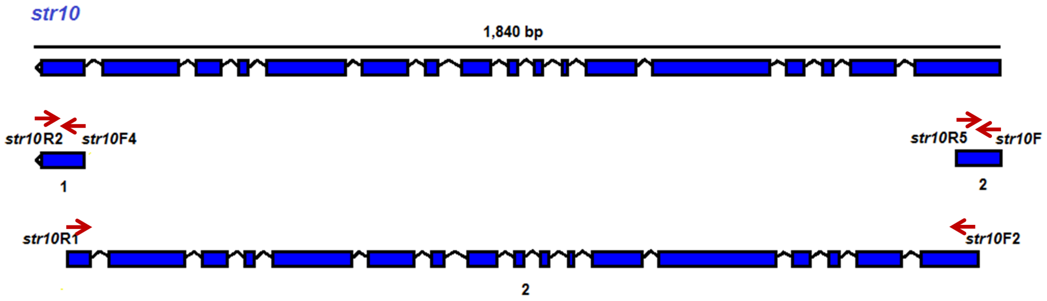

Supplementary Figure 16. The primers positions to amplify *str10*: **Red arrows**, Primer position.

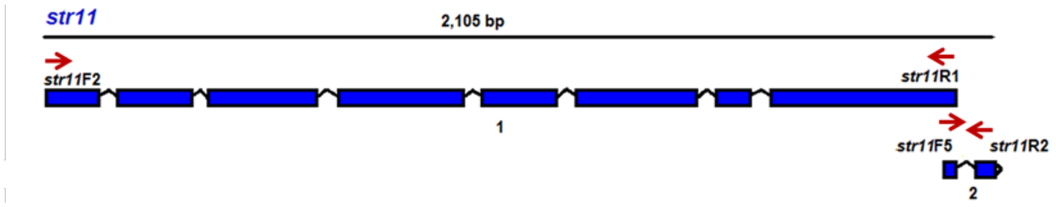

Supplementary Figure 17. Primer positions used to amplify *str11*: **Red arrows**, Primer position.

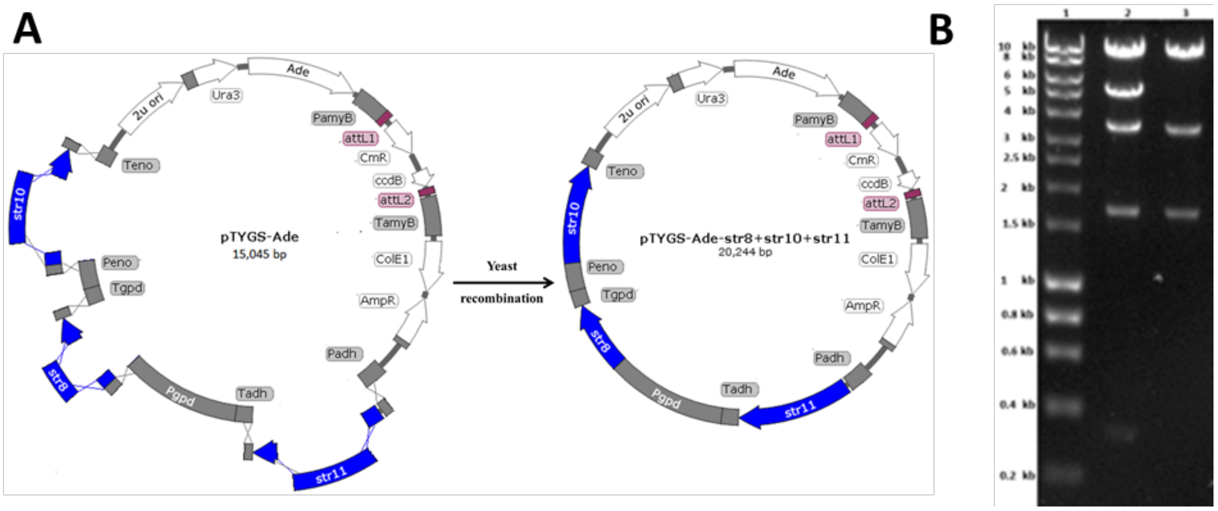

Supplementary Figure 18. A, Construction of pTYGS-Ade-*str8*+*str10*+*str11* from pTYGS-Ade. Size of the plasmids and DNA fragments are not to scale. **B,** Restriction analysis of pTYGS-Ade-*str8*+*str10*+*str11***. Lane 1.** Hyperladder^{TM} 1kb. **Lane 2.** pTYGS-Ade-*str8*+*str10*+*str11* cut with *Eco*RV (9418, 5265, 3533, 1711, 317 bp): **Lane 3.** pTYGS-Ade cut with *Eco*RV (9801, 3533, 1711 bp).

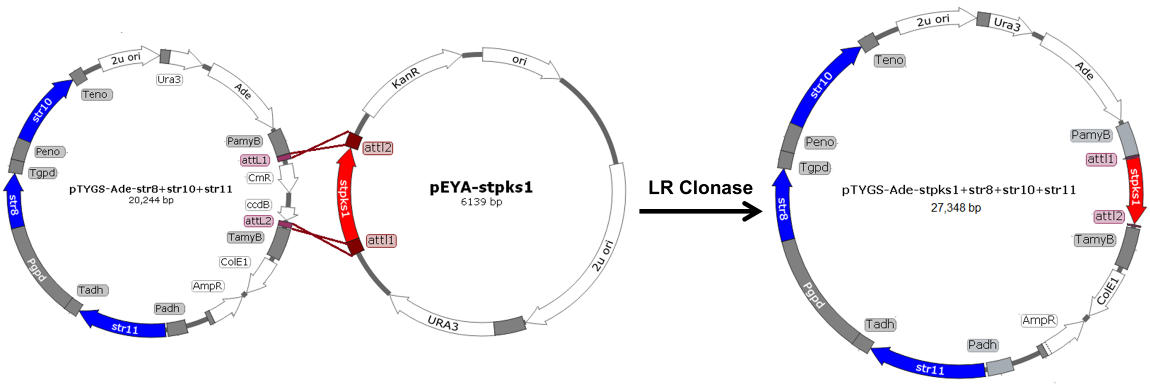

Supplementary Figure 19. Construction of pTYGS-Ade-*stpks1*+*str8*+*str10*+*str11* by the addition of *stpks1* to pTYGS-Ade-*str8*+*str10*+*str11* using Gateway LR Clonase.

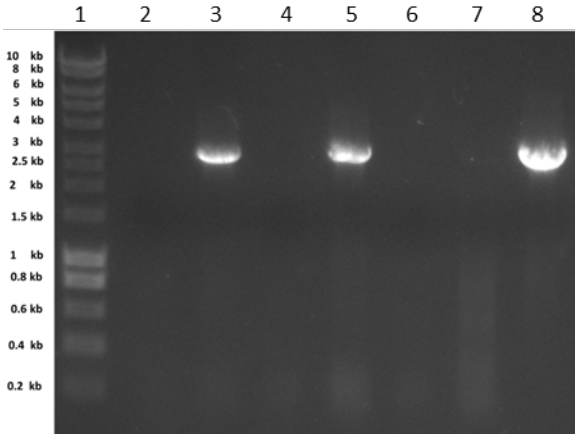

Supplementary Figure 20. Screening of *E. coli* containing pTYGS-Ade-*stpks*+*str8*+*str10*+*str11* by colony PCR using the *stpks1* primers *stpks* F4 and *stpks* R4: **1,** Hyperladder^{TM} 1kb; **2**, Water; lanes **3-7,** Colonies **1-5** respectively; lane **8,** pTAGS-*stpks1* as a positive control.

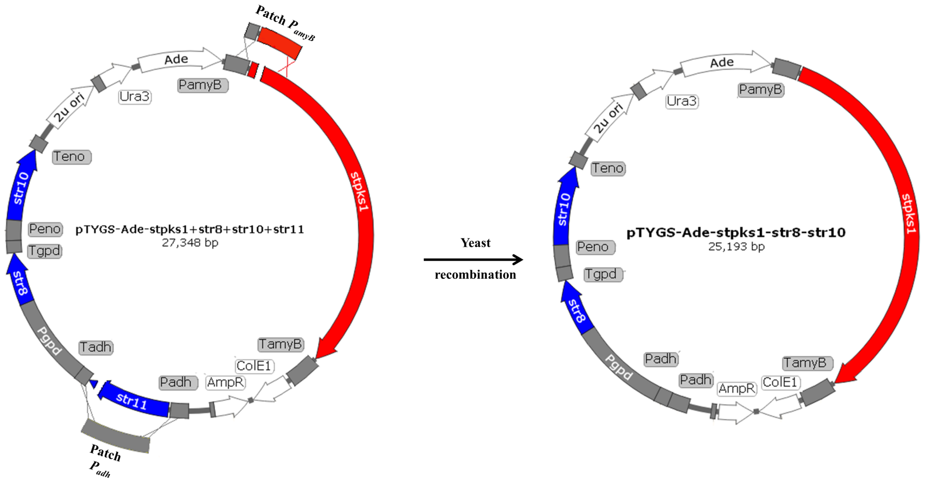

Supplementary Figure 21. Deletion of *str11* from pTYGS-Ade-*stpks1*+*str8*+*str10* by yeast recombination. Sizes of the plasmids and DNA fragments are not to scale.

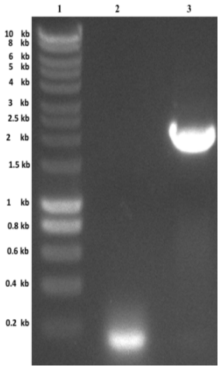

Supplementary Figure 22. Screening for *E. coli* colonies containing pTYGS-Ade-*stpks1*+*str8*+*str10* using colony PCR with primers ExAdhSeq.F and ExAdhSeq.R: **Lane 1,** HyperLadder^{TM} 1kb; **Lane 2,** PCR product from a colony containing pTYGS-Ade-*stpks1*+*str8*+*str10*; **Lane 3,** PCR product from pTYGS-Ade-*stpks1*+*str8*+*str10*+*str11* which is larger due to the presence of *str11.*

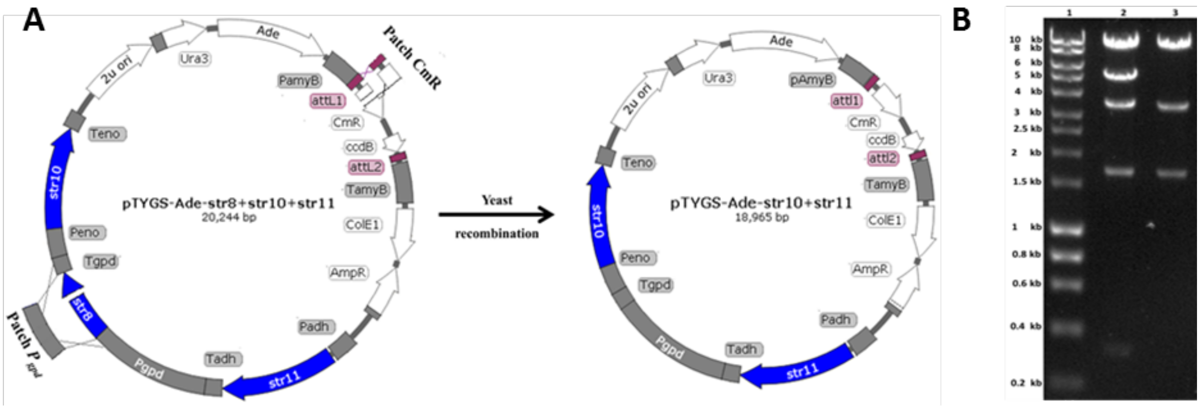

Supplementary Figure 23. A, Construction of pTYGS-Ade-*str10*+*str11* by yeast recombination. **B**, Restriction digest analysis of pTYGS-Ade-*str10*+*str11* using *Eco*RV: **Lane 1,** Hyperladder^{TM} 1kb; **Lane 2,** pTYGS-Ade-*str10*+*str11* cut with *Eco*RV (317 bp, 1711 bp, 3533 bp, 5265 bp and 8139 bp); **Lane 3,** pTYGS-Ade cut with *Eco*RV (1711bp, 3534 bp, and 9801 bp).

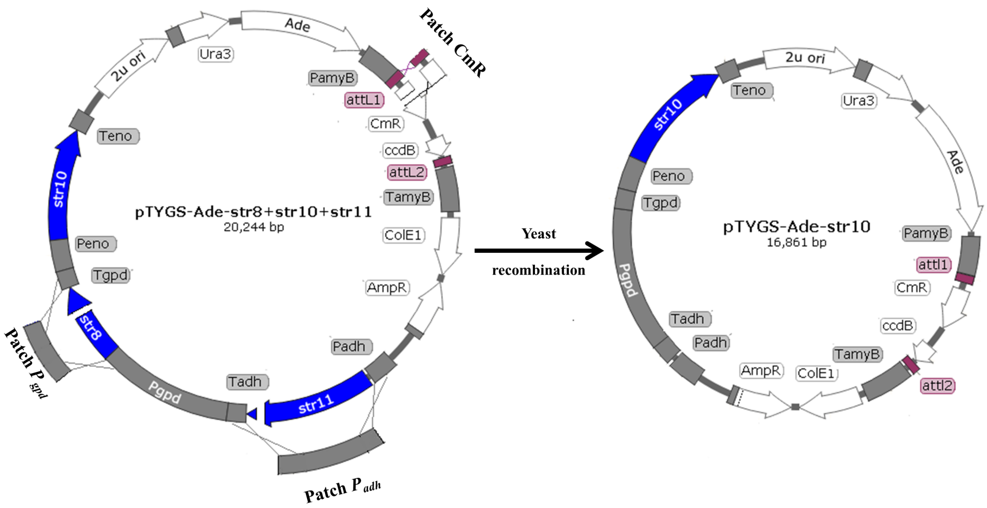

Supplementary Figure 24. Construction of pTYGS-Ade-*str10* by yeast recombination. Sizes of the plasmids and DNA fragments are not to scale.

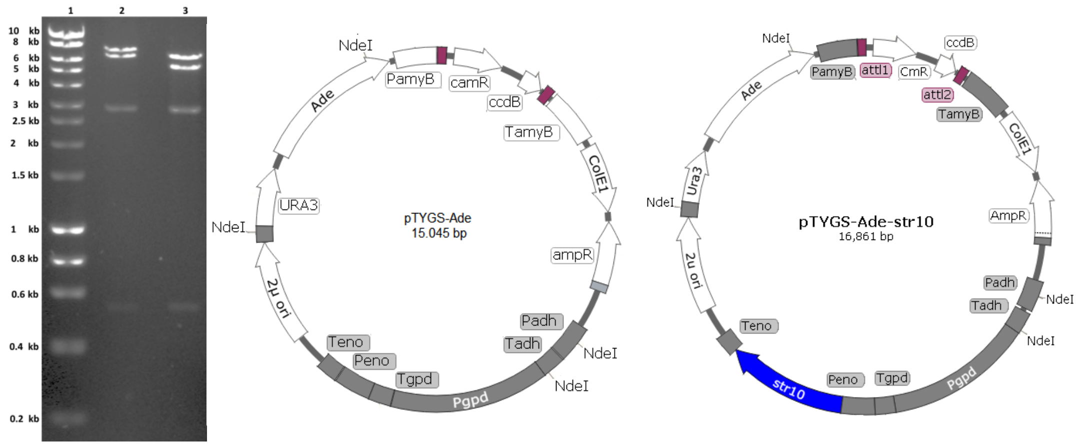

Supplementary Figure 25. Restriction pattern of pTYGS-Ade-*str10* cut with *Nde*I: **Left, Lane 1**, Hyperladder^{TM} 1kb; **Lane 2**, pTYGS-Ade-*str10* digested with *Nde*I (539 bp, 2857 bp, 6310 bp, 7155 bp); **Lane 3**, pTYGS-Ade digested with *Nde*I (547 bp, 2857 bp, 5331 bp, 6310 bp); **Middle,** pTYGS-Ade map showing *Nde*I sites; **Right**, pTYGS-Ade-*str10* map showing *Nde*I sites.

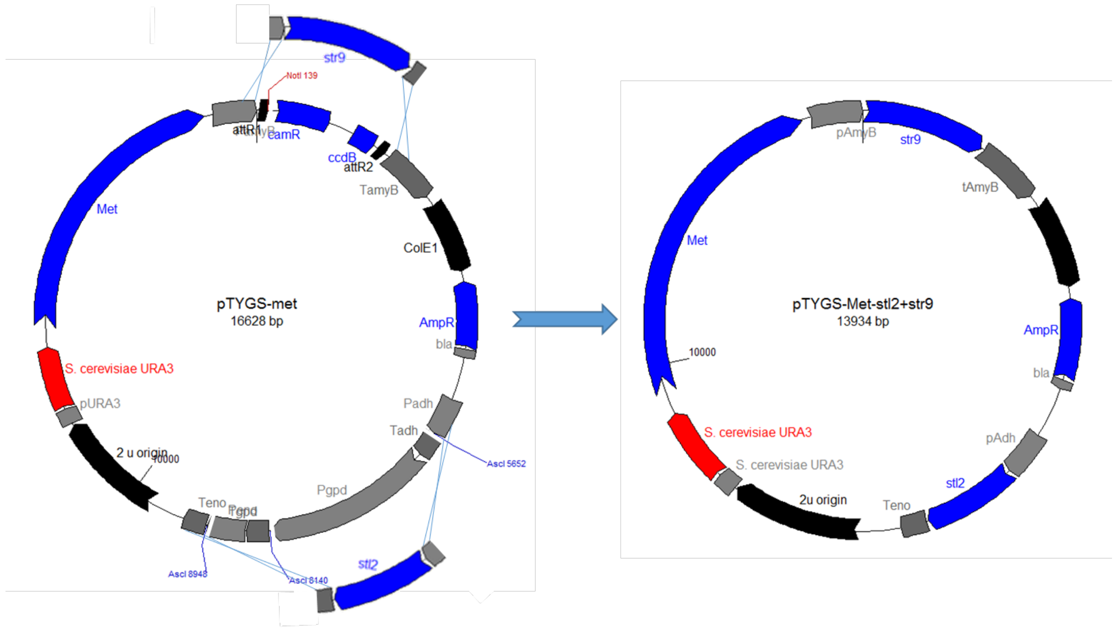

Supplementary Figure 26. Construction of pTYGS-Met-*str9*+*stl2* by yeast recombination.

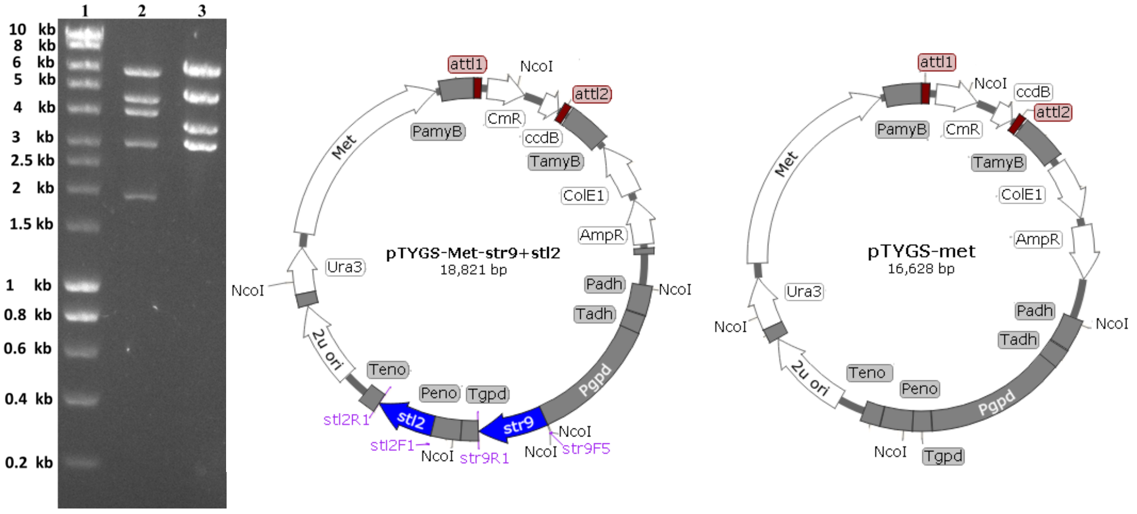

Supplementary Figure 27. Restriction analysis of pTYGS-Met-*str9*+*stl2*. **Left**, **Lane 1**, HyperladderTM 1kb; **Lane 2**, pTYGS-Met-*str9*+*stl2* digested with *Nco*I (5850, 4449, 3968, 2928, 1617 bp); **Lane 3**, pTYGS-Met digested with *Nco*I (5850, 4449, 3276, 2953 bp); **Middle**, pTYGS-Met-*str9*+*stl2* map showing *Nco*I sites; **Right**, pTYGS-Met map showing *Nco*I sites.

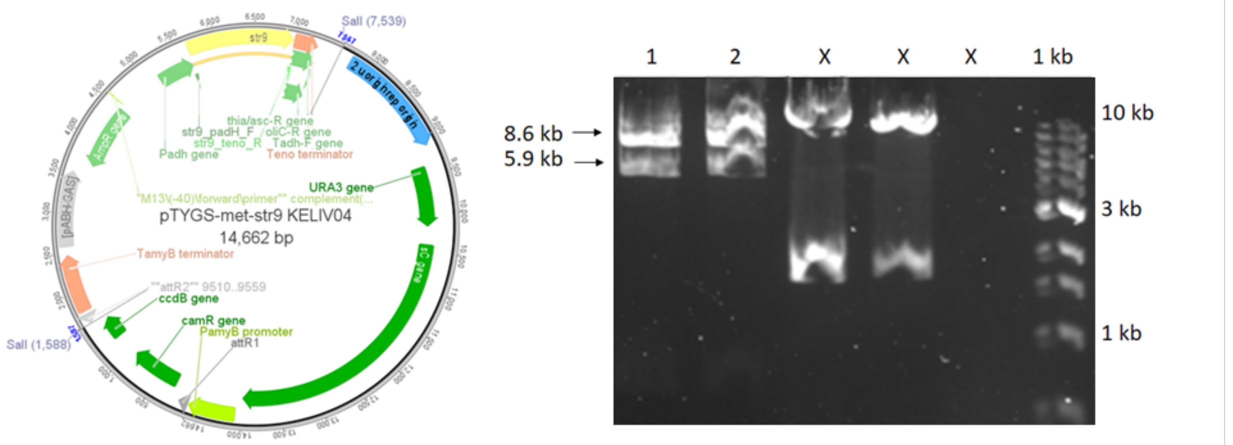

Supplementary Figure 28. Left, Plasmid map of pTYGS-Met-*str9* showing *Sal*I cut sites. **Right**, Lanes 1-2; pTYGS-Met-*str9* digested with *Sal*I showing 5.9 kbp and 8.6 kbp bands. X; another construct, not relevant to this work.

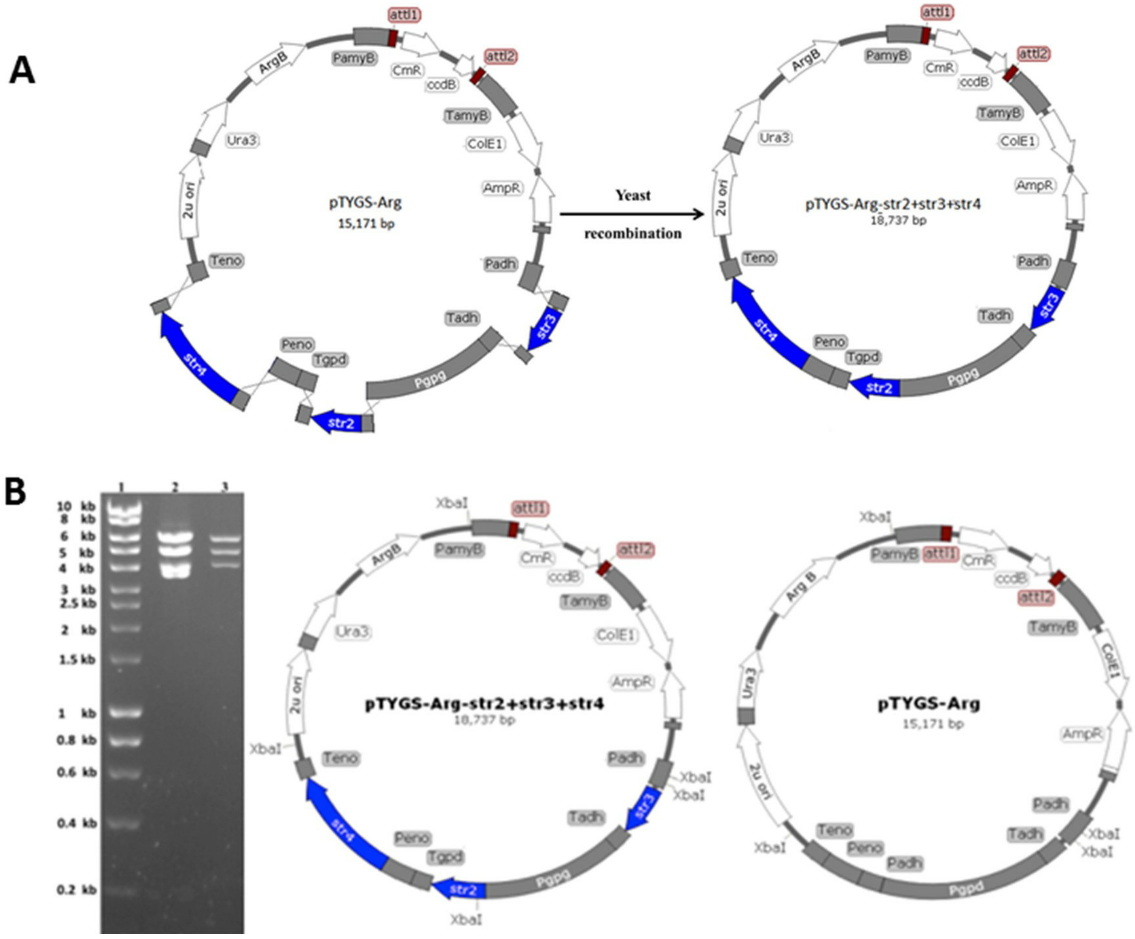

Supplementary Figure 29. A, Construction of pTYGS-Arg-*str2*+*str3*+*str4* by yeast recombination; **B**, Restriction analysis of pTYGS-Arg-*str2*+*str3*+*str4*; **Left, Lane 1**, Hyperladder DNA 1 kb; **Lane 2**, pTYGS-Arg-*str2*+*str3*+*str4* cut with *Xba*I (6003, 5035, 3992, 3576, 131 bp); **Lane 3,** pTYGS-Arg cut with *Xba*I (6003, 5035, 4002, 131 bp); **Middle**, pTYGS-Arg-*str2*+*str3*+*str4* map showing *Xba*I sites; **Left,** pTYGS-Arg map showing *Xba*I sites. Sizes of the plasmids and DNA fragments are not to scale.

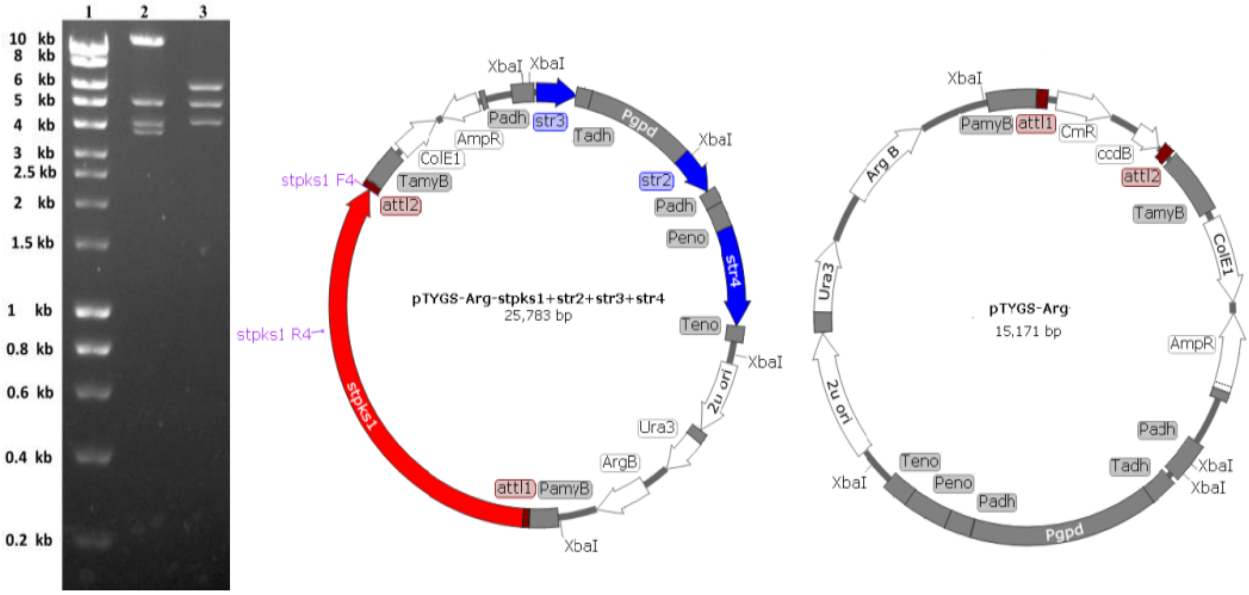

Supplementary Figure 30. Restriction analysis of pTYGS-Arg-*stpks1+str2+str3+str4*. **Left**. **Lane 1**. Hyperladder DNA 1kb. **Lane 2**. pTYGS-Arg-*stpks1+str2+str3+str4* cut with *Xba*I (13049, 5035, 4452, 3576, 131 bp). **Lane 3**. pTYGS-Arg cut with *Xba*I (6003, 5035, 4002, 131 bp): **Middle**. pTYGS-Arg-*stpks1+str2+str3+str4* map showing *Xba*I sites: **Right**. pTYGS-Arg map showing *Xba*I sites.

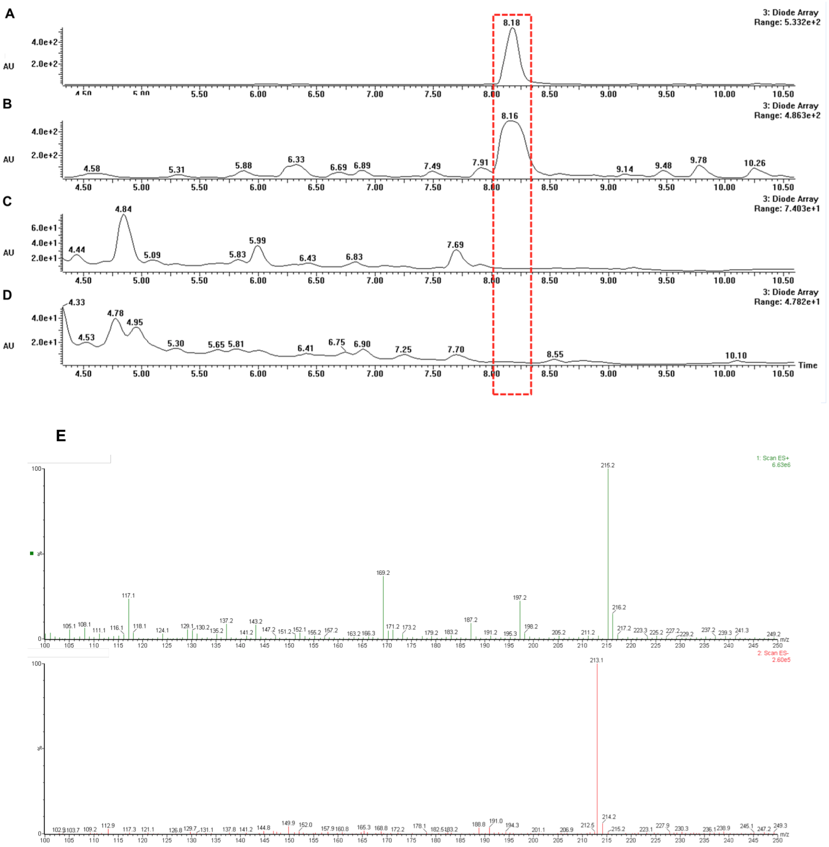

Supplementary Figure 31. Diode Array chromatogram of transformant *stpks1+str10*, showing prestrobilurin **11** production on supplementation with benzoate: **A**, Prestrobilurin **11** standard; **B,** Transformant *stpks1+str10* fed with sodium benzoate; **C,** transformant *stpks1+str10* without benzoate; **D,** Untransformed *A. oryzae* NSAR1; **E**, mass spectrum of **11,** ES+ (top) and ES- (bottom).

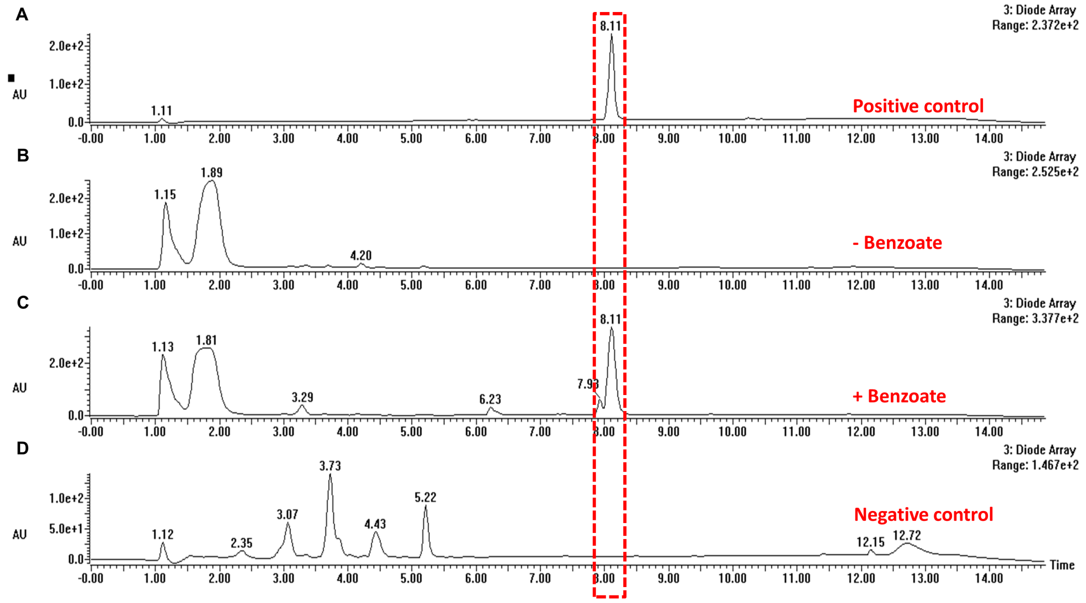

Supplementary Figure 32. Diode Array Chromatogram of Transformant *stpks1+str8+str10* (-PAL): **A**, Prestrobilurin **11** standard; **B**, Transformant *stpks1+str8+str10*; **C**, Transformant *stpks1+str8+str10* fed with 0.1% sodium benzoate; **D**, Untransformed *A. oryzae* NSAR1.

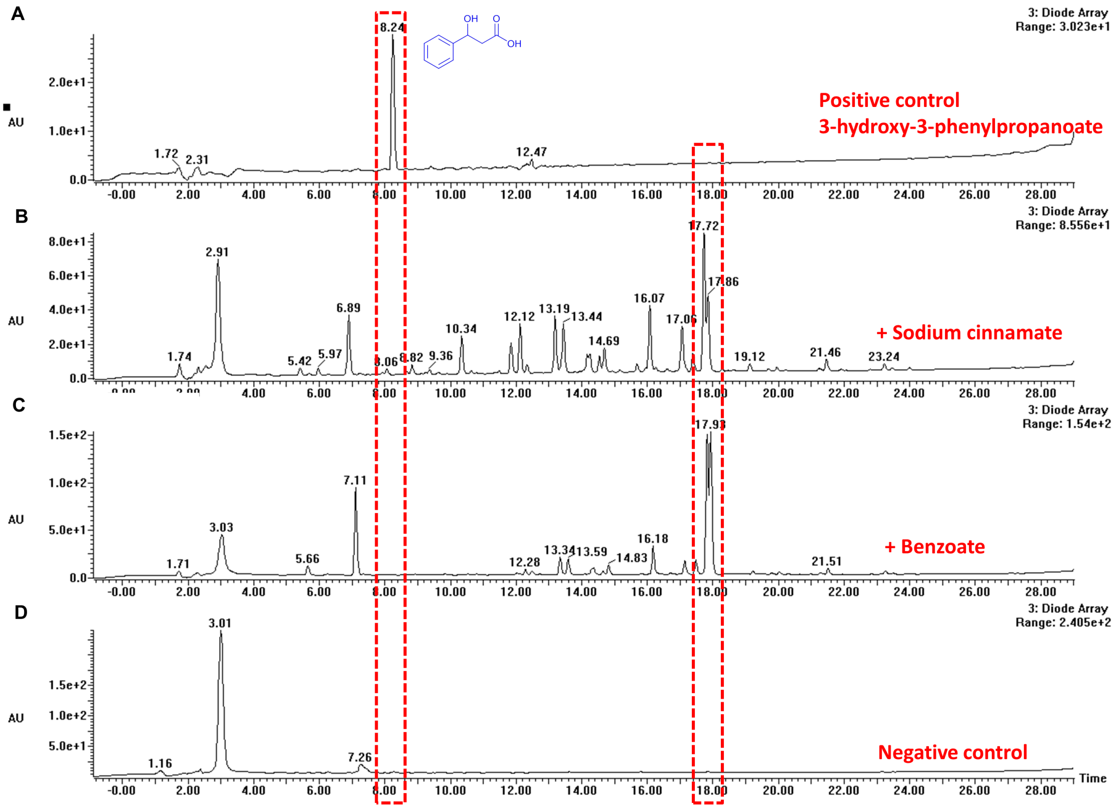

Supplementary Figure 33. Diode Array Chromatogram of transformant *stpks1+str8+str10* supplemented with cinnamate or benzoate, showing prestrobilurin **11** production (approx. 17.7 minutes): **A,** 3-hydroxy-3-phenylpropanoate; **B**, *stpks1+str8+str10* fed with 0.05% sodium cinnamate; **C**. *stpks1+str8+str10* fed with 0.05% sodium benzoate**;** **D**, untransformed *A. oryzae* NSAR1. Peaks at 17.8 min have identical mass spectra and are presumably *E/Z* isomers.

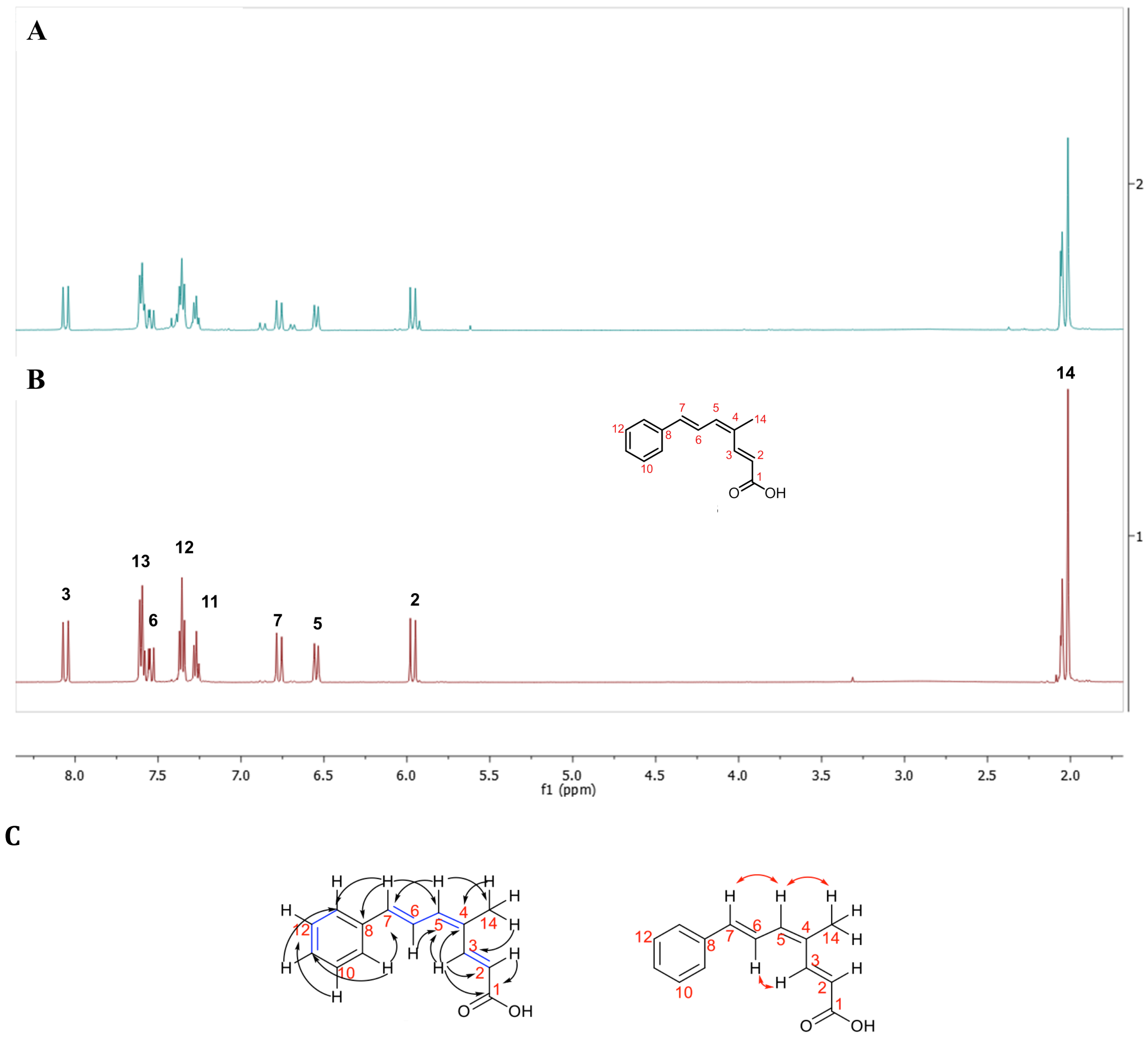

Supplementary Figure 34. NMR analysis of prestrobilurin **11**: **A**, ^{1}H-NMR of Synthetic prestrobilurin **11**; **B**, ^{1}H-NMR of Isolated prestrobilurin **11** from transformant *stpks1+str8+str10+str11.* **C**, Selected NMR correlation data for prestrobilurin **11**: **left**, COSY and selected HMBC correlations for prestrobilurin **11**, **Blue lines** COSY correlations, **Black arrows** Selected HMBC correlations; **right**, NOESY data showing *E*, *Z*, *E* configuration of prestrobilurin **11**.

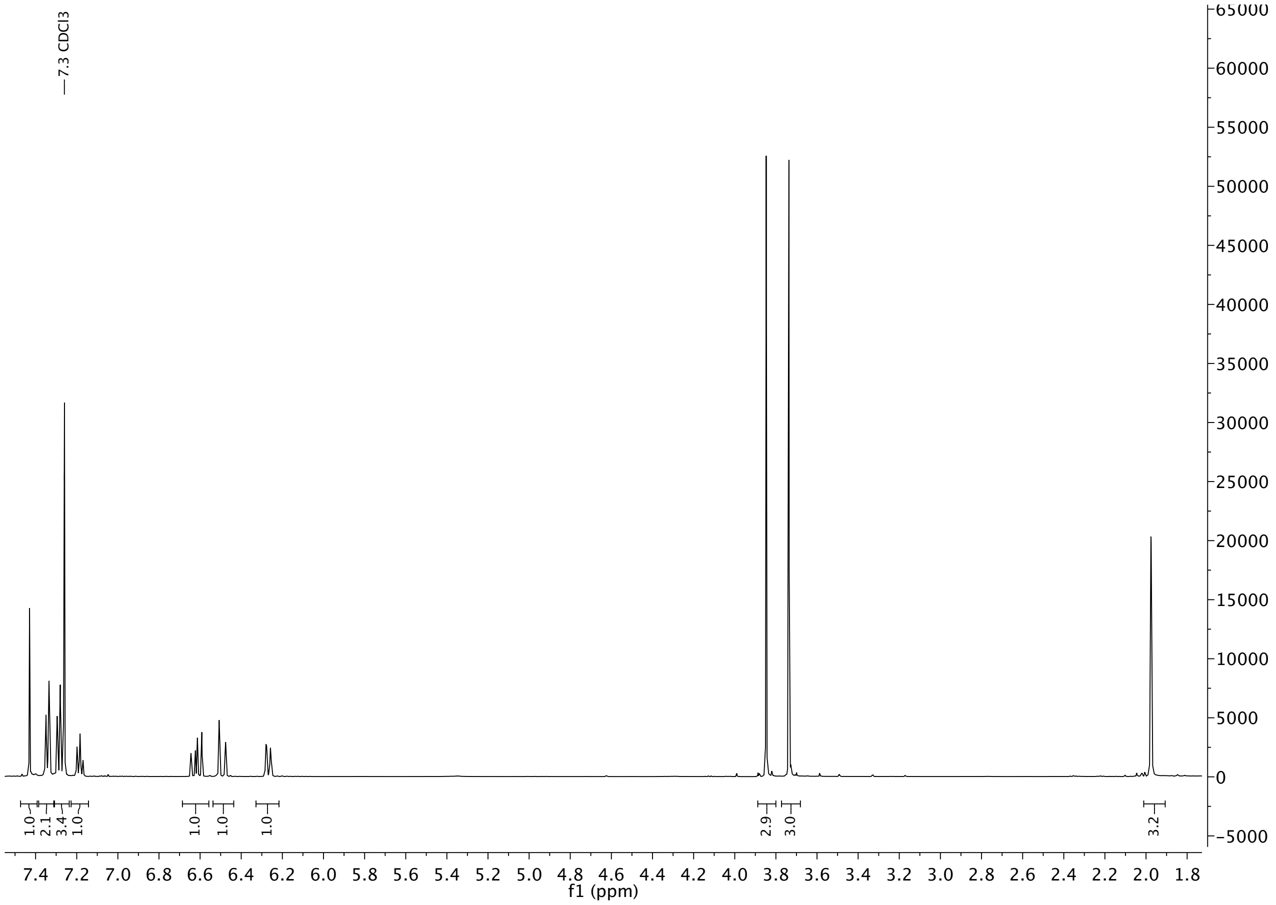

Supplementary Figure 35. ^{1}H NMR of **1** at 500 MHz in $CDCl_3$

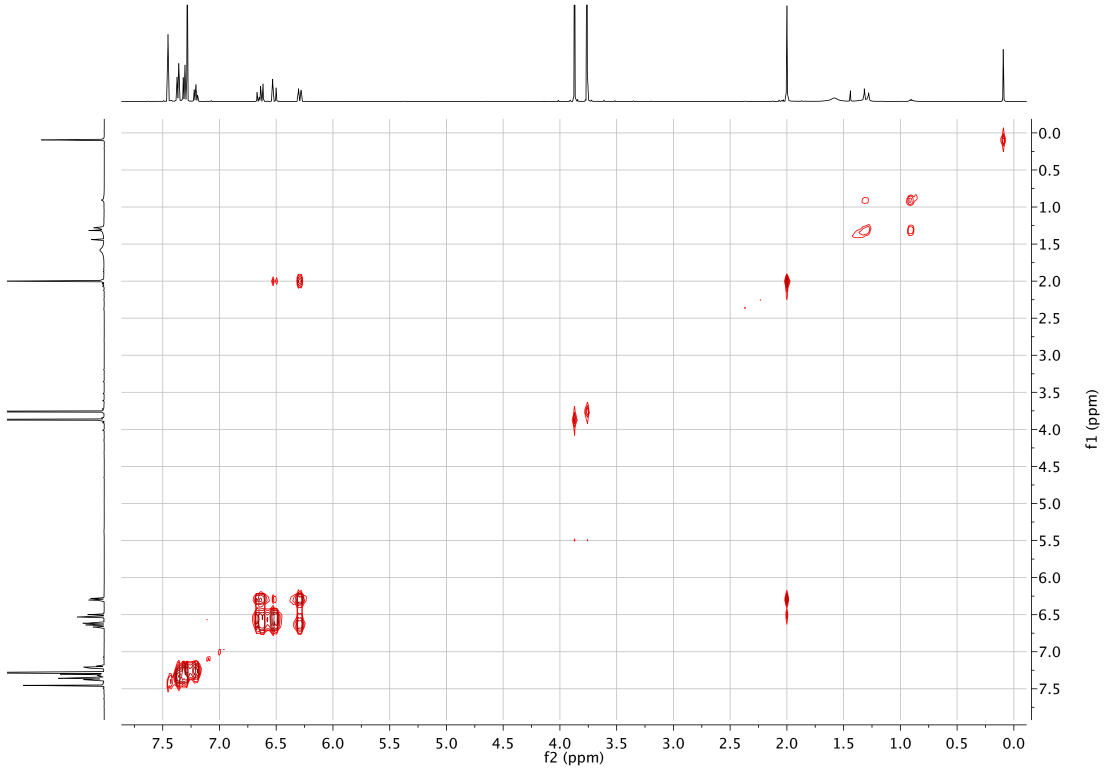

Supplementary Figure 36. COSY NMR of **1** at 500 MHz in $CDCl_3$

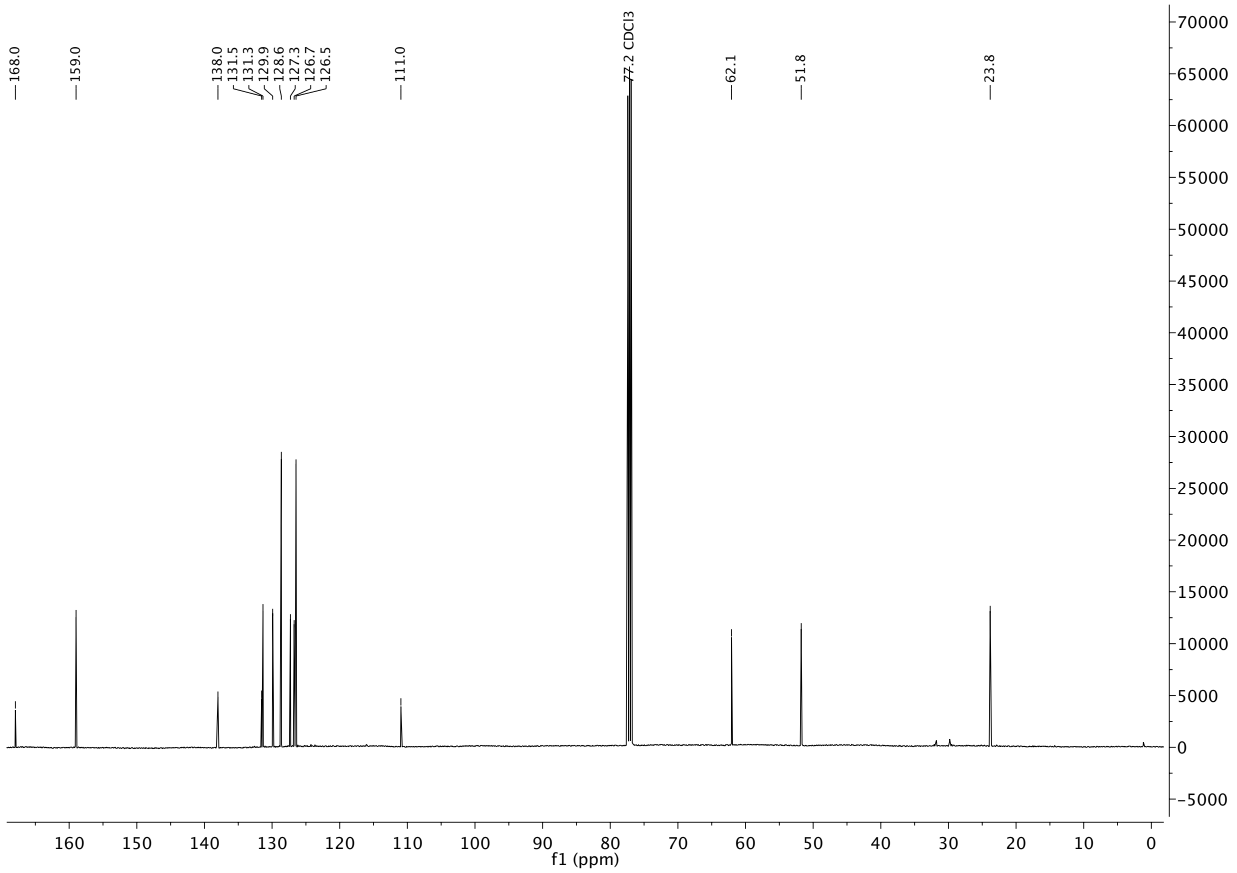

Supplementary Figure 37. ^{13}C NMR of **1** at 125 MHz in $CDCl_3$

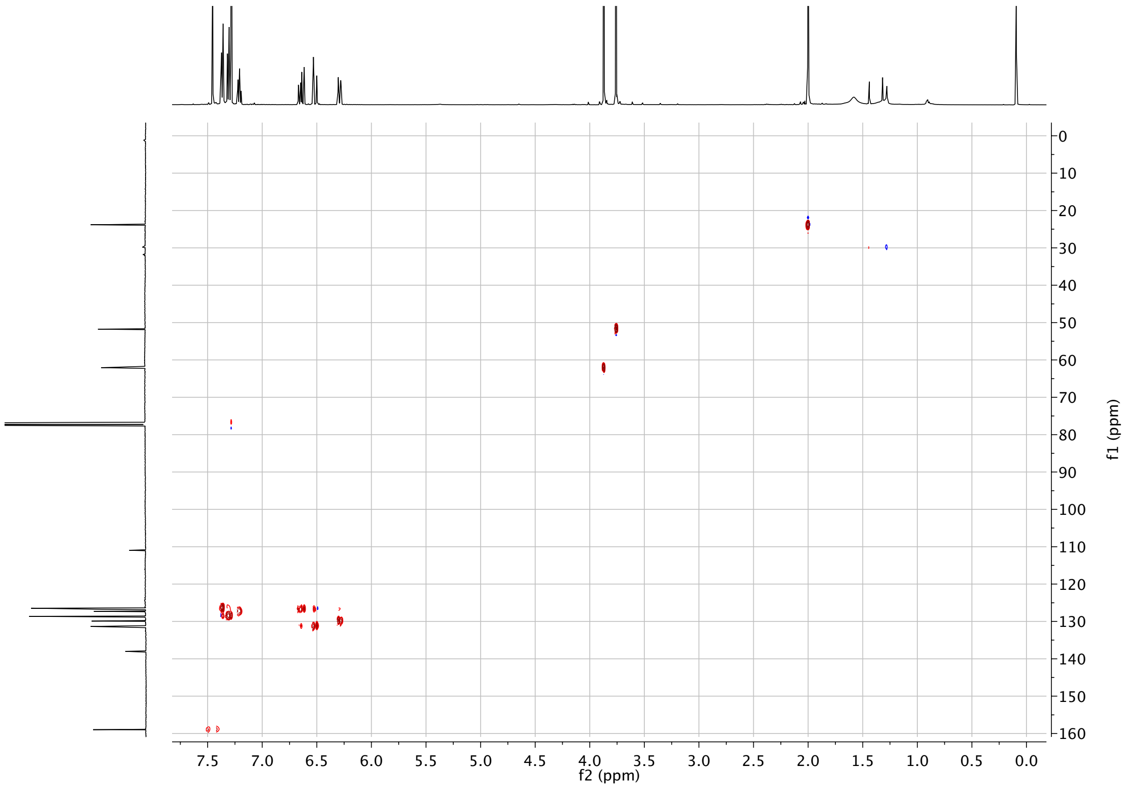

Supplementary Figure 38. HSQC NMR of **1** in $CDCl_3$

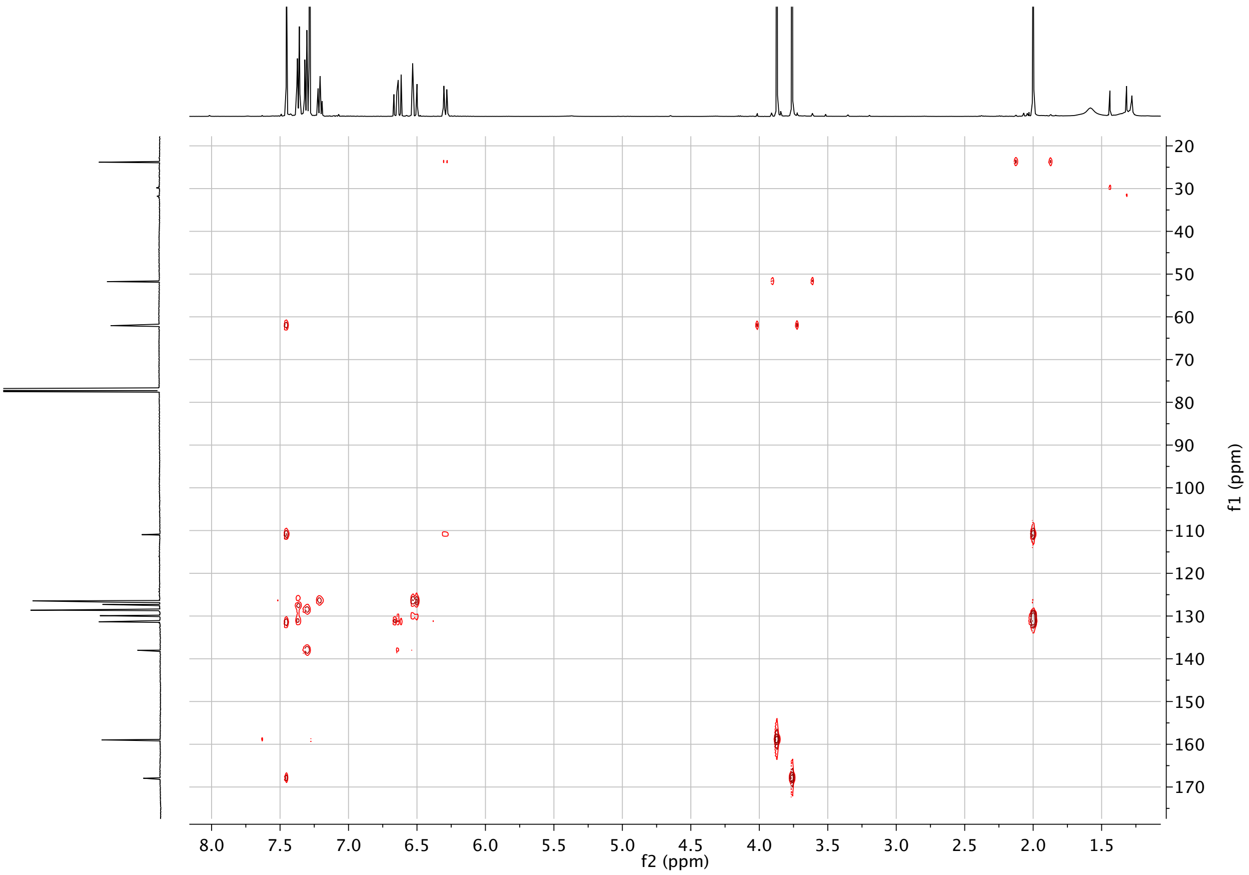

Supplementary Figure 39. HMBC NMR of **1** in $CDCl_3$

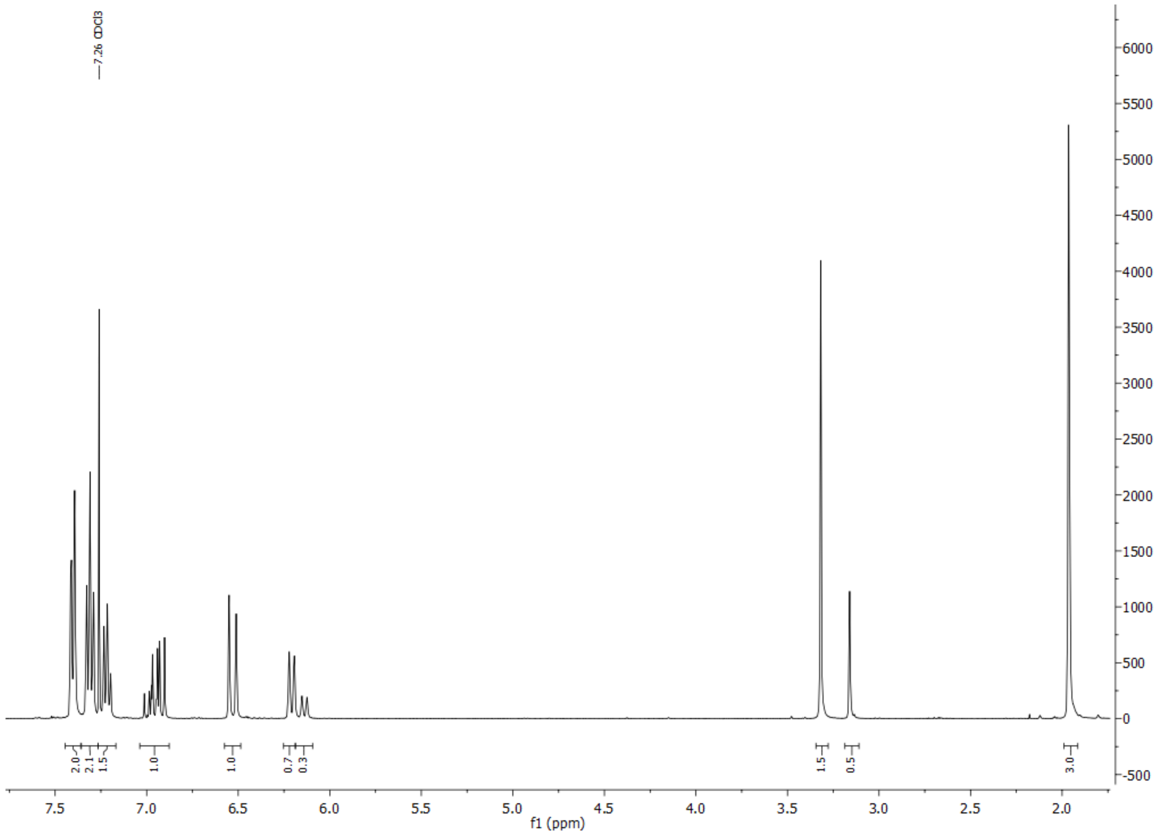

Supplementary Figure 40. ^{1}H NMR of **21** at 125 MHz in $CDCl_3$

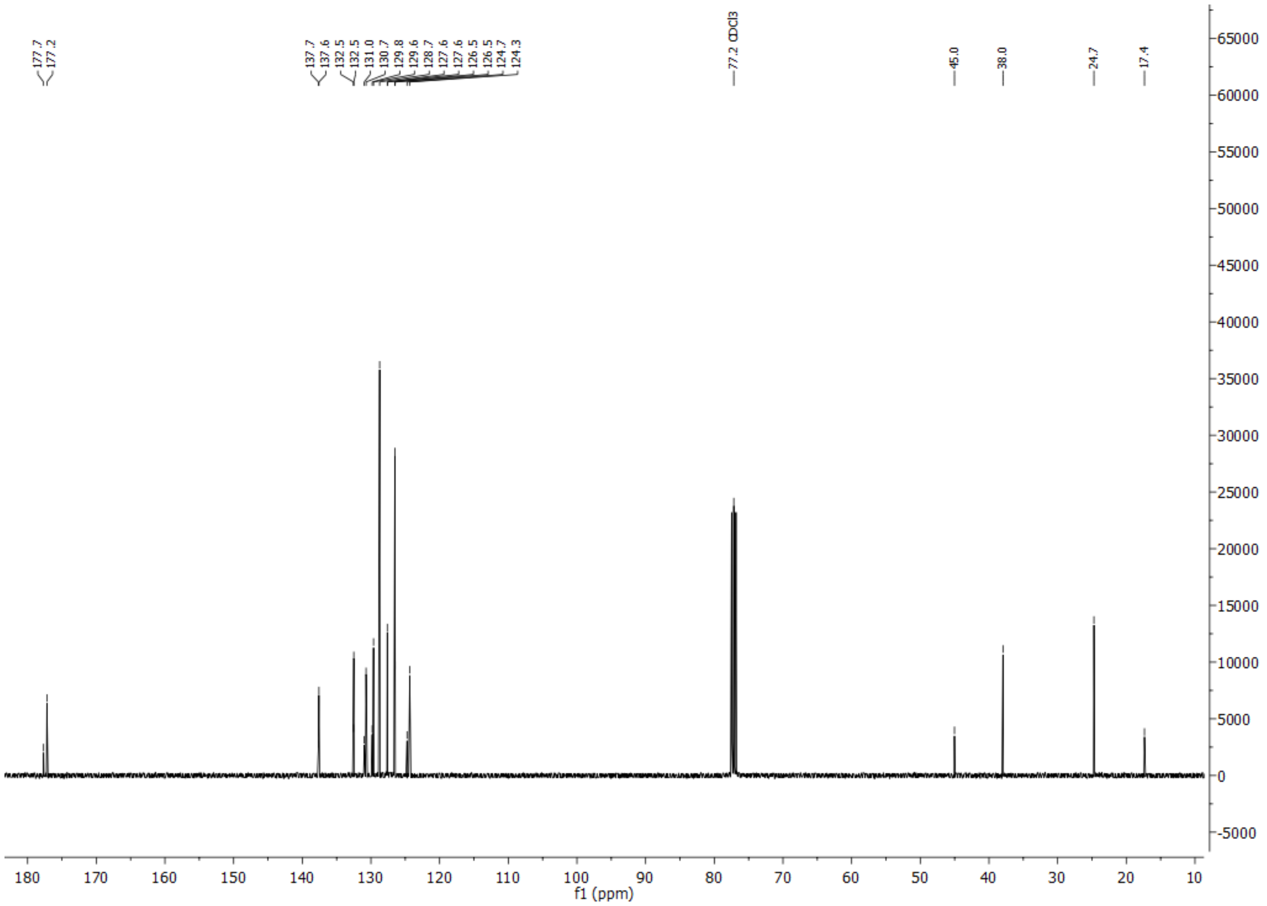

Supplementary Figure 41. ^{13}C NMR of **21** at 125 MHz in $CDCl_3$

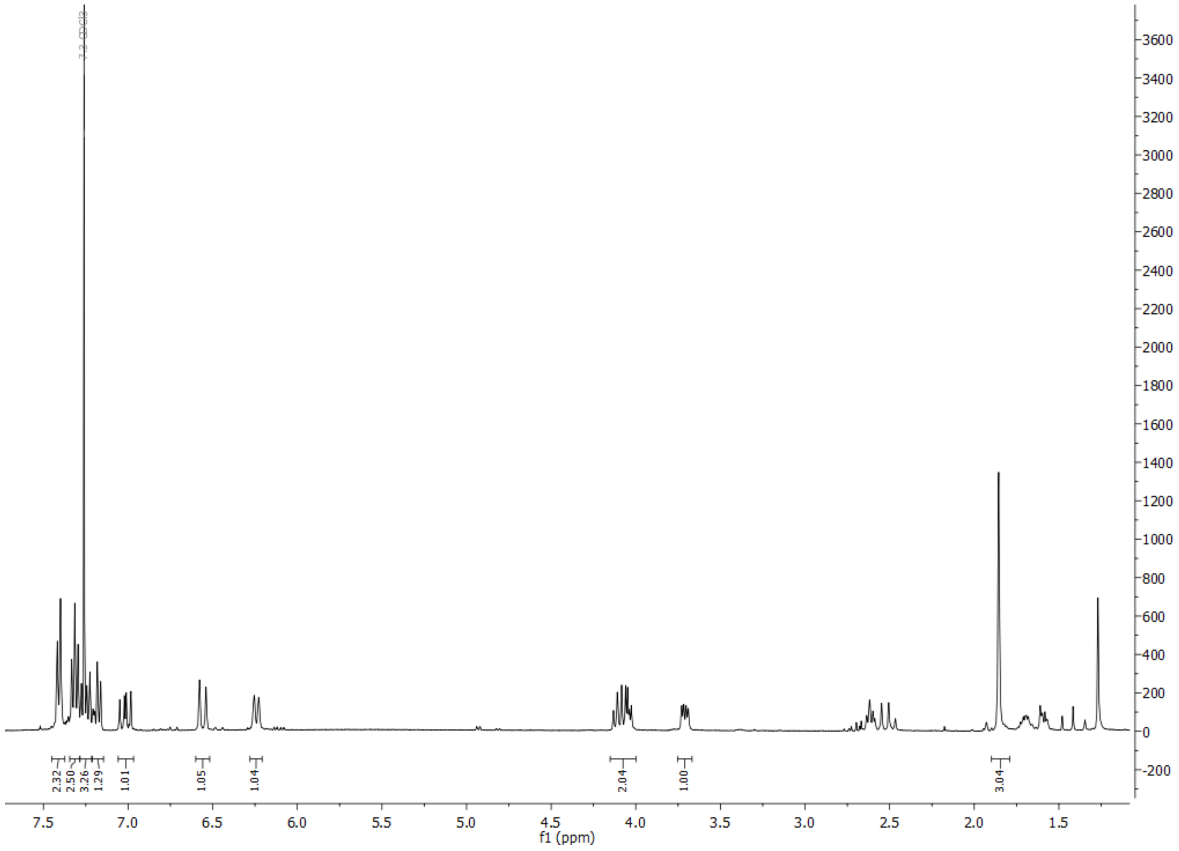

Supplementary Figure 42. ^{1}H NMR of **22** at 125 MHz in $CDCl_3$

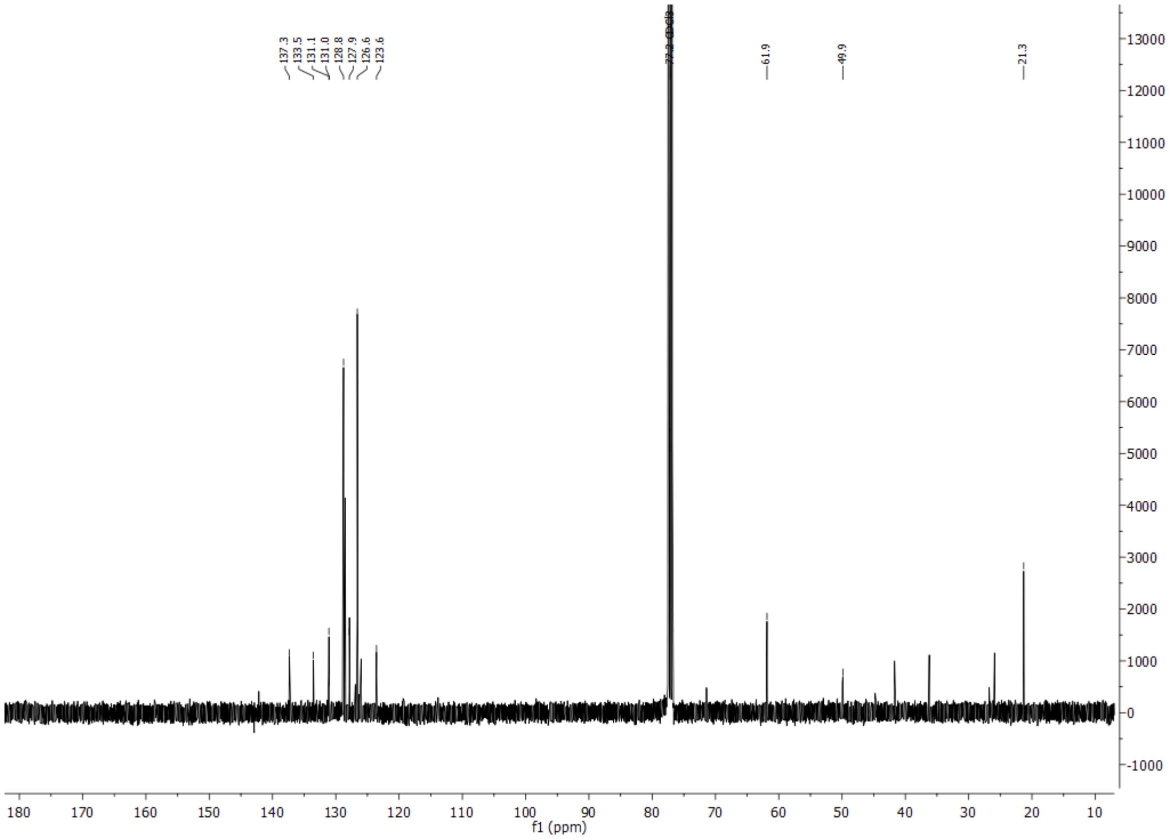

Supplementary Figure 43. ^{13}C NMR of **22** at 125 MHz in $CDCl_3$

```
                                    10        20        30        40        50        60
AFD96009.1(A.o)      MRILKSHPLLKLLNGYLIDSPQPANISYLWNFGSLLALCLVIQIVTGVTLAMHYTPSVLE
Q36551.1(S.t)        MRLLKNNIILRLLNSYMVDSPQPANLTYLWNFGSLLGICLVLQILTGCFLAMHFTPHAEM
S.ten_Cytob          MRTLKDNVMLRTTNSYMVDSPQPANITYLWNFGSLLGICLVLQILTGCFLAMHFTPHAEM
S.lutea_Cytob        MRTLKDNMMLRTTNSYMVDSPQPANITYLWNFGSLLGICLVLQILTGCFLAMHFTPHAEM
                     ** **.: :*:  *.*::*******::*********.:***:**:**  ****:** .

                                    70        80        90       100       110       120
AFD96009.1(A.o)      AFNSVEHIMRDVNNGWLVRYLHANTASAFFFLVYLHIGRGLYYGSYKSPRTLTWAIGTVI
Q36551.1(S.t)        AFNSVEHIMRDVQSGWIVRYTHANVASFFFIFVYAHIGRGLYYNSYKSPRVLLWSIGVII
S.ten_Cytob          AFNSVEHIMRDVQSGWMVRYTHANVASFFFIFVYAHIGRGTYYNSYKSPRVLLWSIGVIM
S.lutea_Cytob        AFNSVEHIMRDVQSGWMVRYTHANVASFFFIFVYAHMGRGTYYNSYKSPRVLLWSIGVIM
                     ************:.**:*** ***.** **::** *:*** **.******.* *:**.::

                                   130       140       150       160       170       180
AFD96009.1(A.o)      LIVMMATAFLGYVLPYGQMSLWGATVITNLMSAIPWIGQDIVEFIWGGFSVNNATLNRFF
Q36551.1(S.t)        LVLMMAIGFLGYVIPFGQMSLWGATVITNLLSAIPVFGQDIVELIWGGFSVSNATLNRFF
S.ten_Cytob          TVLMMAMGFLGYVIPFGQMSLWGATVITNLLSAIPVFGTDIVELIWGGFSVSNATLNRFF
S.lutea_Cytob        TVLMMAMGFLGYVMPFGQMSLWGATVITNLLSAMPVFGQDIVELIWGGFSVSNATLNRFF
                      ::***  .*****:*:*************:**:* :* ****:*******.********

                                   190       200       210       220       230       240
AFD96009.1(A.o)      ALHFLLPFVLAALALMHLIAMHTVGSGNPLGISGNYDRLPFAPYFIFKDLVTIFIFFIVL
Q36551.1(S.t)        SLHYILPFVLAALVVAHFMALHIHGSNNPNGVTSNTDRYPMYPYFIFKDLVTIFAFFWIL
S.ten_Cytob          SLHYILPFVLAALVVAHFMATHIHGSNNPNGVTSNTDRYPMYPYFMFKDLVTMFAFFWML
S.lutea_Cytob        SLHYILPFVLAALVVAHFMATHMHGSNNPNGVTSNTDRYPMYPYFMFKDLVTMFAFFWML
                     :**::********.: *::* *  **.** *::.* ** *: ***:******:* ** :*

                                   250       260       270       280       290       300
AFD96009.1(A.o)      SIFVFFMPNALGDSENYVMANPMQTPPAIVPEWYLLPFYAILRSIPNKLLGVIAMFAAIL
Q36551.1(S.t)        SVIVFFYPNLMGHQDNYIPADPMVTPASIVPEWYLLPFYAILRSIPDKLLGVVAMFGSLL
S.ten_Cytob          SVIVFFYPNTMGHQDNYMPADPMVTPASMVPEWYTLPFYAMTRSIPDKLLGVVAMFGSLL
S.lutea_Cytob        SVIVFFYPNTMGHQDNYMPADPMVTPASMVPEWYTLPFYAMTRSIPDKLTGVVAMFGSLL
                     *::*** ** :*..:**: *:** **.::***** *****: ****:** ***.***.::*

                                   310       320       330       340       350       360
```

```
AFD96009.1(A.o)      ALMVMPITDLSKLRGVQFRPLSKVAFYIFVANFLVLMQIGAKHVETPFIEFGQISTVLYF
Q36551.1(S.t)        ILLVLPLTDLSRIRGNQFRPAMKFFFWFFVVNFIMLFWLGSQHPNTPYLEIGQLSTTFYF
S.ten_Cytob          MLLMLPLTDTSRMRGNQFRPAMKLFFWFFVVNFMMLFWIGSQHPNTPYLEIGQLSTTFYF
S.lutea_Cytob        MLLVLPLTDTSRMRGNQFRPAMKLFFWFFVVNFMMLFWIGSQHPNTPYLEMGQLSTTFYF
                      *:::*:** *::** ****  *.  *::**.**::*: :*::* :**::*:**:**.:**

                                370       380      389
AFD96009.1(A.o)      AYFFVIVPVVSLIENSLVELATKK-----
Q36551.1(S.t)        SFFLVIVPFTGLVENTLLDLNIKELDLNL
S.ten_Cytob          SFFLVIVPVTGLVENTTLDLNIKKLSLNT
S.lutea_Cytob        SFFLVIVPITGLVENTTLDLNIKKLGLNM
                     ::*:****...*:**: ::*  *:
```

Supplementary Figure 44. Alignment of the publicly available *S. tenacellus* cytochrome b protein sequence (Q36551.1) with a homologue from the publicly available *A. oryzae* genome (AFD96009.1) and the predicted proteins from the *S. lutea* and *S. tenacellus* strains sequenced in this work. As with the previously published sequence, both novel sequences lack the G143A or F129L substitutions (highlighted in blue), but do have glutamine at position 254 and aspartate at position 261 (highlighted in red). The *A. oryzae* sequence lacks any of the substitutions previously identified as encoding resistance to strobilurins and related ß-methoxyacrylate antifungal compounds.

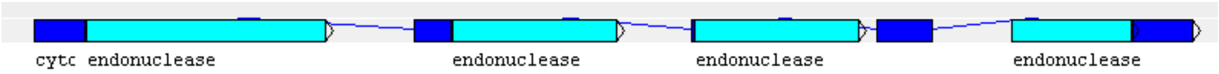

>Q36551.1(S.ten_cytob_NCBI)
MRLLKNNIILRLLNSYMVDSPQPANLTYLWNFGSLLGICLVLQILTGCFLAMHFTPHAEMAFNSVEHIMRDVQSGWIVRY
THANVASFFFIFVYAHIGRGLYYNSYKSPRVLLWSIGVIILVLMMAIGFLGYVIPFGQMSLWGATVITNLLSAIPVFGQD
IVELIWGGFSVSNATLNRFFSLHYILPFVLAALVVAHFMALHIHGSNNPNGVTSNTDRYPMYPYFIFKDLVTIFAFFWIL
SVIVFFYPNLMGHQDNYIPADPMVTPASIVPEWYLLPFYAILRSIPDKLLGVVAMFGSLLILLVLPLTDLSRIRGNQFRP
AMKFFFWFFVVNFIMLFWLGSQHPNTPYLEIGQLSTTFYFSFFLVIVPFTGLVENTLLDLNIKELDLNL

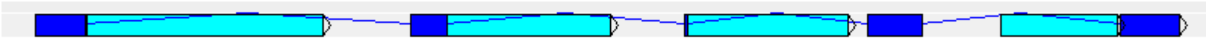

>S.ten_Cytob
MRTLKDNVMLRTTNSYMVDSPQPANITYLWNFGSLLGICLVLQILTGCFLAMHFTPHAEMAFNSVEHIMRDVQSGWMVRY
THANVASFFFIFVYAHIGRGTYYNSYKSPRVLLWSIGVIMTVLMMAMGFLGYVIPFGQMSLWGATVITNLLSAIPVFGTD
IVELIWGGFSVSNATLNRFFSLHYILPFVLAALVVAHFMATHIHGSNNPNGVTSNTDRYPMYPYFMFKDLVTMFAFFWML
SVIVFFYPNTMGHQDNYMPADPMVTPASMVPEWYTLPFYAMTRSIPDKLLGVVAMFGSLLMLLMLPLTDTSRMRGNQFRP
AMKLFFWFFVVNFMMLFWIGSQHPNTPYLEIGQLSTTFYFSFFLVIVPVTGLVENTTLDLNIKKLSLNT

Supplementary Figure 45. Structure of the cytochrome b gene of *S. tenacellus* and *S. lutea*. Dark blue boxes represent the exons of the cytochrome b gene. Light blue boxes represent ORFs contained within the type 1 introns of the gene, which all encode putative LAGLIDADG type homing endonuclease / maturase proteins (Supplementary Table 8)

>S.ten-Intron1 endonuclease
MGLHNLQTKDWIFSNGTKTVIMNNTYSMQTFTLNAIVLMKKMIDEFLNIKDKTLLNKSKFSGLVDNTETESDFLQWFAGF
SDAEASFMISLKNNSSFGEAHFIFRMTLHIDDCAVLFTIRERLGIGVVSMRNQTCTYSVHSFQNMMNVTLPIFDKYPLLT
LKQLDYSDWRKAMILKKENKDQTSKMSLSTTTFNQMSHIKNNMNTNRTDYQNYKLKKEMISKYWLVGFVEGDGSFFFTNG
NAVFSTTQKDKRMLEVIRDYLENIPLSPPYKGLVKPSKPHCSMTRKNNNTAYQLDIQDKDVLFQYITPFFKNLQFFSRKG
IDFSIWCLGLHILINGYNHLFKGKELTLKLSNNMNSKRYFSDISELLDIQDTKETFDTQPPFDIYSGKSHFVLAKEFHAK
KGSRRGYKVYVYKNGIQMKESPFHSYRDCCKVLDVISPSSIKNYIDSGKPYKEYQFYSTLQIFS

>S.ten-Intron2 endonuclease
MENLTLMTKDFFFFNNLSEPSTYIFSNMGIMTTNGQTMEKTFLPTMGTVSPHALKGKKKIRKDKKEYTSMPSQFMAFFVG
LMDGDGYIQVTKTTKGFMTMKLTTTLSLDDLSTLEYIHSVWGLGLMTMSKDRLNPICTYRMNRTDLQEVMFPLLHYHKIF
FFMNSRRKQYNKALYILKNDVKLYDEKFTNNIPNEFNLPDSSYEYTNLNFYKNWLMGFTNSEGSFLIKKNNDGCFQLKQR
IDTTLFESFNLLFKSNRKMDMETDKYAQFSVSSKKDIQEVMNYFSFSGLHPLVGKKNIQYFKWLNDLQNSKRYKDLNYPL

>S.ten-Intron3 endonuclease
MDTEEPYNSDIVTKTQTIAGTFPKWNIRYYPSRTKVVKKTMRRKKPAGIRHFLNVGMPQRTNAVETKKAMLVGFVEKYGW
FYVFKKGKYLMYEFGMELSIRDVQLIYKMKEFLGMGTIMFRNTEGRSKTVIYRVRNKSHLKAIIFPMFDKYPLLSNKQKD
YLRFRDATFKGSMTFENLNSEDIHKNKDLNSVDSMTSAYYLPSWLVGFMEAEGCFTMYQPSKSHSKVASFEMSKTNGENL
ILAMSKYLSFTQKIHIDKTNCFKLKISSVRSVENIIKFMQKAPVKLKGHKKLQYTLWTKETRQIPRYTTKFSIPDMY

>S.ten-Intron4 endonuclease
MNFISATLISVLLFAVGSTKGIKKTSKLQREAISINDETMSCITGMMLSDGHIQQRSVTGNARFIFAQSGKPSKREYFNL
VLEMMKPFCSANYVPYAKEWKDTRSKTMNSSISTTTMQTPCFSRLHNTWYYNNMKMVPSNIKEMTTPLATAHWMMGDDSK
QNEGIHLSVYAFTTSDVQLLMTALNERYNTECSMHIPDRGPRIYTNKLNMVNLKPLVSPYMVTLWNMK

Supplementary Figure 46. Predicted *S.tenacellus* endonuclease protein sequences identified within the type 1 introns of the cytochrome b gene.

>S.lutea_Cytob
MRTLKDNMMLRTTNSYMVDSPQPANITYLWNFGSLLGICLVLQILTGCFLAMHFTPHAEMAFNSVEHIMRDVQSGWMVRY
THANVASFFFIFVYAHMGRGTYYNSYKSPRVLLWSIGVIMTVLMMAMGFLGYVMPFGQMSLWGATVITNLLSAMPVFGQD
IVELIWGGFSVSNATLNRFFSLHYILPFVLAALVVAHFMATHMHGSNNPNGVTSNTDRYPMYPYFMFKDLVTMFAFFWML
SVIVFFYPNTMGHQDNYMPADPMVTPASMVPEWYTLPFYAMTRSIPDKLTGVVAMFGSLL
MLLVLPLTDTSRMRGNQFRPAMKLFFWFFVVNFMMLFWIGSQHPNTPYLEMGQLSTTFYFSFFLVIVPITGLVENTTLDL
NIKKLGLNM

>S.lutea-Intron1 endonuclease
MMLHNLQTKDWIFSNGTETLIKNNTYSMQFFTLNAIVLMKKMIDEFLNIKDKTLLNKSKYSGTEDNTETESDFLQWFVGF
SDAESSFMISLKNNSPSTSTPPPRRGGVGEAHFIFRMTLHIDDCAVLFTIRERLGIGVVSMRNQTCTYSVHSFQNMMNVT
LPIFDKYPLLTLKQLDYSDWRKAMILKKENKDQSSKMSLSTTTFNQMSHIKNNMNTNRTDYQNYKLKKEMISKYWLVGFV
EGDGSFFFTNGNAVFSTTQKDKRMLEVIRDYLENIPLNPPYKGLVKPSRGKPHCSMTRKN
KNTAYQLDIQDKDVLFQYITPFFKNLQFFSRKGIDFSIWCLGLHILINGYNHLFKGKELTLKLSNNMNSKRYFSDISELL
DIQDTKETFDTQPPFDIYSGKSHFVLAKEFHAKKGSRRGYKVYVYKNGIQMKESPFHSYRDCCKVLDVISPSSIKNYIDS
GKPYKEYQFYSTLQIFS

>S.lutea-Intron2 endonuclease
MENLTLMTKDFFFFNNLSEPSTYIFSNMGIMTTNGQTMENTFLPTMGTVSPHAWKKKIRKDKKEYTSMPSQFMAFFVGLM
DGDGYIQVTKTTKGFMTMKLTTTLSLDDLSTLEYIHSVWGLGLMTMSKDRLNPICTYRMNRTDLQEVMFPLLHYHKIFFL
MNSRRKQYNKALYILKNDVKLYDEKFTNNIPNEFNLPDSSYEYTNLNFYKNWLMGFTNSEGSFLIKKNNDGCFQLKQRID
TTLFESFNLLFKSNRKMDMETDKYAQFSVSSKKDIQEVMNYFSFSGLHPLVGKKNIQYFKWLNDLQNSKRYKDLNYPL

>S.lutea-Intron3 endonuclease
MNTEEPYNSDIVTQTQTIAGTFPKWNIRYYPSTTKVVKKTMRRKKPAGIRHYLNVGMPQRTNAVETKKAMLVGFVEGDGW
FSVSKKGKYLMYEFGMELSIRDVQLIYKMKEFLGMGTIMFRNTEGRSKTVIYRVRNKSHLKAIIFPMFDKYPLLSNKQYD
YLRFRDATFKGIMTFENLNSEYIRPNNDLNSVDSMTSASYFSSWLVGFMEAEGCFSIYQPSNSNSKVASFEMSQTNGENL
ILAMSKYLSFTQKIHKDKTNCFKLKISSVRSVENIIKFMQKAPVNLKGHKNLQYTLWTKETRQIPRYTTKFSIPDMY

>S.lutea-Intron4 endonuclease
MNLIFDTLISVLLFAVRSTKGIKKTSKLQREAISINDETMSCITGMMLSDGHIQQRSVTGNARFIFAQSGKPSKREYFNL
VLEMMKPFCSANYVPYAKEWKDTRSKTMNSSISTTTMQTPCFSRLHNTWYYNNMKMVPSNIKEMTTPLALAHWMMGDGSK
QNEGIHLSVYAFTTSDVQLLMTALNERYNTECSMHMTDRGPRIYMNKMNMVNLKPLVSPYMVPSMKYKMGN

Suplememtary Figure 47. Predicted *S. lutea* endonuclease protein sequences identified within the type 1 introns of the cytochrome b gene.

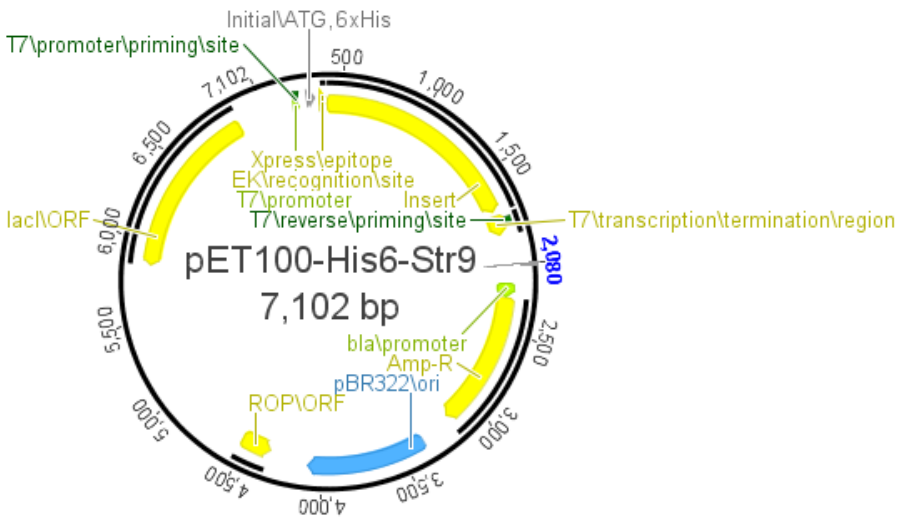

Supplementary Figure 48. Plasmid map of pET100-His_6-Str9.

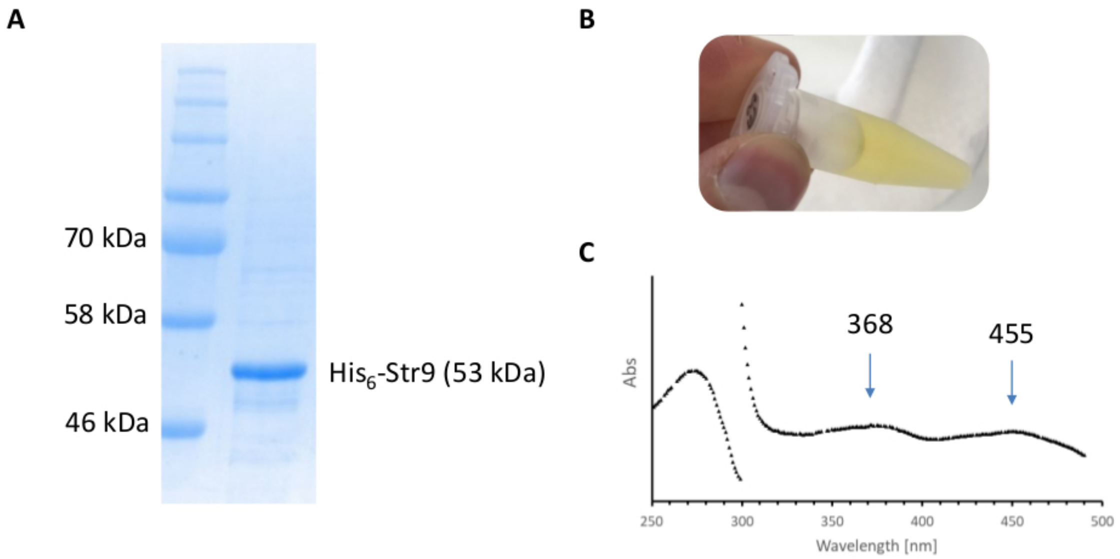

Supplementary Figure 49. A, SDS-PAGE analysis of purified and concentrated His$_6$-Str9, 12% polyacrylamide gel, protein pre–stained marker; **B**, Elution fraction after Ni-NTA of His$_6$-Str9 showing its yellow colour (image K. Lebe); **C**, UV/VIS spectrum of purified His$_6$-Str9 with FAD specific wavelength maxima at 368 nm and 455 nm.

Accession	Description	Score	Coverage	# Proteins	# Unique Peptides	# Peptides	# PSMs	# AAs	MW [kDa]	calc. pI
str9	str9	6641.30	72.97	1	28	28	168	481	53.2	5.85

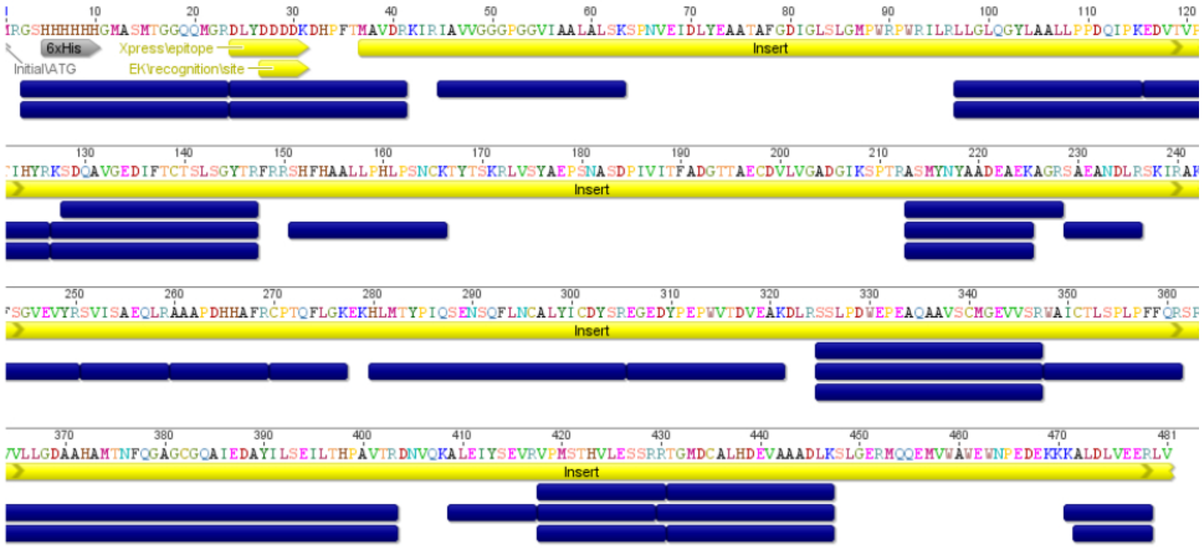

Supplementary Figure 50. ESI–MS/MS analysis of His6–Str9. The expressed protein band was cut from the SDS PAGE and confirmed by mass spectrometry using the methods of Klodmann and coworkers.[4] Blue bars indicate detected peptide fragments.

ATGGCCGTGGATCGTAAAATTCGTATTGCAGTTGTTGGTGGTGGTCCTGGTGGTGTTATTGCAGCACTGGCACTGAGCAA
AAGCCCGAATGTTGAAATTGATCTGTATGAAGCAGCAACCGCCTTTGGTGATATTGGTCTGAGCCTGGGTATGCCGTGGC
GTCCGTGGCGCATTCTGCGTCTGCTGGGTCTGCAGGGTTATCTGGCAGCCCTGCTGCCTCCGGATCAGATTCCGAAAGAA
GATGTTACCGTTCCGACCATTCATTATCGTAAAAGCGATCAGGCAGTTGGCGAAGATATTTTCACCTGTACCAGCCTGAG
CGGTTATACCCGTTTTCGTCGTAGCCATTTTCATGCCGCACTGCTGCCGCATCTGCCGAGCAATTGTAAAACCTATACCA
GCAAACGTCTGGTTAGCTATGCAGAACCGAGCAATGCAAGCGATCCGATTGTTATTACCTTTGCAGATGGCACCACCGCA
GAATGTGATGTTCTGGTTGGTGCAGATGGTATTAAAAGTCCGACACGTGCAAGCATGTATAACTATGCAGCAGATGAAGC
AGAAAAAGCAGGTCGTAGTGCCGAAGCAAATGATCTGCGTAGCAAAATTCGTGCAAAATTTAGCGGTGTTGAAGTGTATC
GTAGCGTTATTAGCGCAGAACAGCTGCGTGCAGCAGCACCGGATCATCATGCATTTCGTTGTCCGACACAGTTTCTGGGT
AAAGAAAAACATCTGATGACCTATCCGATTCAGAGCGAAAATAGCCAGTTTCTGAATTGTGCCCTGTACATTTGTGATTA
TAGCCGTGAAGGTGAAGATTATCCGGAACCGTGGGTTACCGATGTTGAAGCCAAAGACCTGCGTAGTAGCCTGCCGGATT
GGGAACCTGAAGCACAGGCAGCAGTTAGCTGTATGGGTGAAGTTGTTAGCCGTTGGGCAATTTGTACCCTGAGTCCGCTG
CCGTTTTTTCAGCGTAGCCGTGTTGTGCTGCTGGGTGATGCAGCACATGCAATGACCAATTTTCAAGGTGCAGGTTGTGG
TCAGGCAATTGAAGATGCATATATTCTGAGCGAAATTCTGACCCATCCGGCAGTTACCCGTGATAATGTTCAGAAAGCAC
TGGAAATCTATAGCGAAGTTCGTGTTCCGATGAGCACCCATGTTCTGGAAAGCAGCCGTCGTACCGGTATGGATTGTGCA
CTGCACGATGAAGTTGCAGCAGCCGATCTGAAAAGCCTGGGTGAACGTATGCAGCAAGAAATGGTTTGGGCATGGGAATG
GAATCCGGAAGATGAAAAAAAGAAAGCCCTGGATCTGGTTGAAGAACGCCTGGTTTAA

Supplementary Figure 51. Codon optimised sequence of *str9*.

MRGSHHHHHHGMASMTGGQQMGRDLYDDDDKDHPFTMAVDRKIRIAVVGGGPGGVIAALALSKSPNVEIDLYEAATAFGD
IGLSLGMPWRPWRILRLLGLQGYLAALLPPDQIPKEDVTVPTIHYRKSDQAVGEDIFTCTSLSGYTRFRRSHFHAALLPH
LPSNCKTYTSKRLVSYAEPSNASDPIVITFADGTTAECDVLVGADGIKSPTRASMYNYAADEAEKAGRSAEANDLRSKIR
AKFSGVEVYRSVISAEQLRAAAPDHHAFRCPTQFLGKEKHLMTYPIQSENSQFLNCALYICDYSREGEDYPEPWVTDVEA
KDLRSSLPDWEPEAQAAVSCMGEVVSRWAICTLSPLPFFQRSRVVLLGDAAHAMTNFQGAGCGQAIEDAYILSEILTHPA
VTRDNVQKALEIYSEVRVPMSTHVLESSRRTGMDCALHDEVAAADLKSLGERMQQEMVWAWEWNPEDEKKKALDLVEERL
V

Supplementary Figure 52. Protein sequence of recombinant Str9.

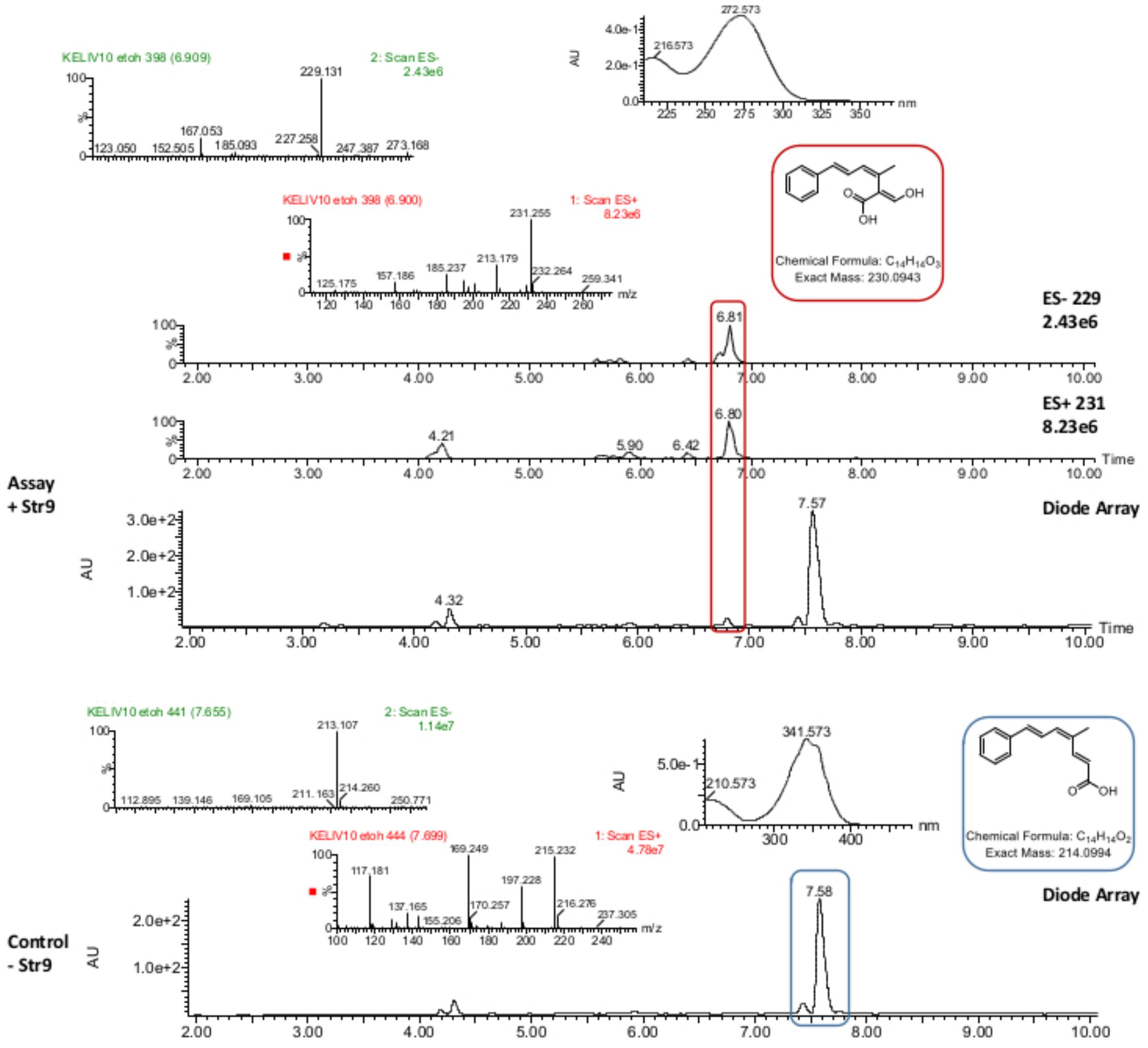

Supplementary Figure 53. LCMS analysis of Str9 *in vitro* assay with prestrobilurin A **11** (blue box) demonstrating the conversion to oxidation product highlighted in red. From top: Low resolution MS data and uv data for oxidised product eluting at 6.8 min; LCMS spectra of assay extract showing DAD data and extracted ion chromatograms corresponding to the molecular ions of the oxidised product; Low resolution MS data and uv data for prestrobilurin A **11** eluting at 7.6 min; LCMS spectra of assay extract showing DAD data and extracted ion chromatograms corresponding to the molecular ions of **11**.

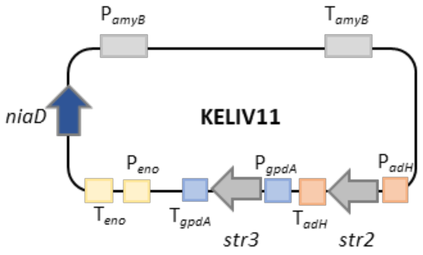

Supplementary Figure 54. Plasmid map of KELIV11 pTYGSniaD-str2-str3.

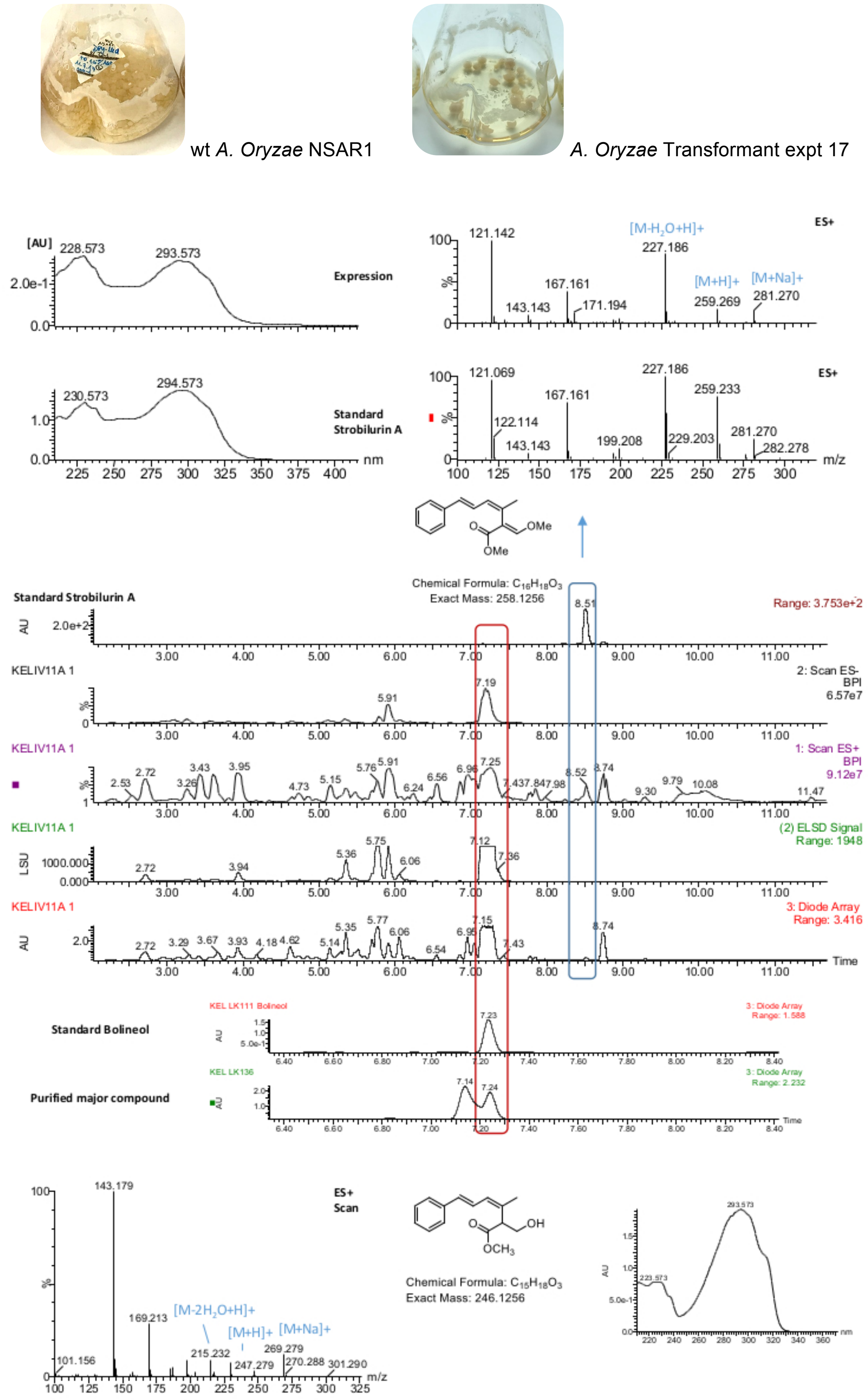

Supplementary Figure 55. Detailed LCMS analysis for experiment 17. From top: fermentations of wild-type A. Oryzae NSAR1 and a transformant from experiment 17 after 7 days of growth; uv and MS analysis of strobilurin A detected at Rt 8.5 min compared with pure standard; LCMS analysis for culture extract from expt 17 showing (from top) extracted ion chromatogram for 259.1, total ion current ES- and ES+, ELSD and DAD traces; DAD chromatograms of standard bolineol (Rt 7.2 min) and bolineol purified from the experiment. MS and uv dta for bolineol detected in the experiment. Photographs K. Lebe.

Supplementary Tables

Supplementary Table 1. Distribution of nucleotides in the genomes of *S. lutea* F23523 and *S. tenacellus*.

Nucleotide	*S. lutea*. F23523		*S. tenacellus*	
	Count (bp)	Frequency	Count (bp)	Frequency
Adenine (A)	10, 283, 445	24.1 %	9, 700, 825	23.9%
Cytosine (C)	10, 992, 169	25.8%	10, 523, 129	26.0%
Guanine (G)	10, 998, 111	25.8%	10, 539, 856	26.0%
Thymine (T)	10, 283, 478	24.1%	9, 695, 192	23.9%
Any nucleotide (N)	47, 681	0.1%	88, 187	0.2%

Supplementary Table 2. Genome assembly statistics for *S. lutea* F23523 and *S. tenacellus*.

**Parameter**	**Length**	
	***S. lutea* F23523**	***S. tenacellus***
N_{75}	27 355	46 297
N_{50}	63 543	121 343
N_{25}	129 610	258 346
Minimum	1 002	1 000
Maximum	553 944	988 442
Average	20 925	20 847
Count	2 036	1 944
Total genome size	42 603 884	40 527 189

Supplementary Table 3. AntiSMASH identified gene clusters in *S. lutea* F23523 and *S. tenacellus.*

***S. lutea* F23523**		***S. tenacellus***	
**Contig**	**Gene Type**	**Contig**	**Gene Type**
**Sl-298**	**t1pks**	**St-2**	**t1pks**
**Sl-1078**	**t1pks**	**St-273**	**t1pks**
Sl-88	terpene	St-43	terpene
Sl-117	terpene	St-78	terpene
Sl-246	terpene	St-186	terpene
Sl-267	terpene	St-255	terpene
Sl-447	terpene	St-276	terpene
Sl-891	terpene	St-288	terpene
Sl-132	lantipeptide	St-296	siderophore
Sl-184	siderophore	St-2	putative
		St-19	putative
Sl-23	putative	St-39	putative
Sl-42	putative	St-42	putative
Sl-61	putative	St-42	putative
Sl-86	putative	St-45	putative
Sl-105	putative	St-55	putative
Sl-155	putative	St-66	putative
Sl-439	putative	St-82	putative
Sl-621	putative	St-118	putative
Sl-1074	putative	St-129	putative
Sl-20	other	St-232	putative
Sl-190	other	St-35	other
Sl-293	other	St-60	other
Sl-342	other	St-71	other
Sl-342	other	St-90	other
Sl-360	other	St-105	other
Sl-392	other	St-160	other
Sl-447	other	St-233	other
Sl-695	other	St-288	other

Supplementary Table 4. Annotation of genes found on contigs St-2 and Sl-1078.

Gene cluster St-2	Putative Function	Gene cluster Sl-1078	Putative Function
stl5	Hypothetical Protein	*slr5*	Cytochrome P450
stl4	Hypothetical Protein	*slr4*	Hydroxymethylglutaryl-CoA synthase
stl3	Hypothetical Protein	*slr3*	Hypothetical protein
stl2	Endonuclease	*slr2*	FAD-dependent oxidoreductase
stl1	Hypothetical Protein	*slr1*	Hydrolase
stpks2	PKS	*slpks2*	PKS
stlr1	Heat shock protein	*slr1*	Heat shock protein
str2	NADH oxidase	*slr2*	Hypothetical Protein
str3	Hypothetical Protein	*slr3*	Glutathione transferase

Supplementary Table 5. Annotation of strobilurin BGCs and surrounding genes.

Strobilurus lutea F23523					***Strobilurus tenacellus***				
**Gene Name**	**Function**	**Boundaries**	**Size (bp)**	**Size amino acids)**	**Gene Name**	**Function**	**Boundaries**	**Size (bp)**	**Size (amino acids)**
sll18	Tropinone reductase/opsin-5-like	301-1991	870	290					
sll17	MFS General Substrat Transporter	2143-3567	768	256					
sll16	Hypothetical protein	3995-5779	1184	3943					
sll15	Triphosphate Hydrolase	6077-10397	3367	1122					
sll14	Hypothetical Protein	10725-12066	1144	381					
sll13	Hypothetical Protein	13525-14781	1257	418					
sll12	Hypothetical Protein	15177-15863	581	193					
sll11	Cyclin	17030-18889	1644	547					
sll10	Hypothetical Protein	19409-21079	906	302					
sll9	Acetyltransferase	21716-22406	531	176					
sll8	Kinase	22661-24001	1212	403					
sll7	Acid phosphatase	24402-26388	1254	417					
sll6	Glycoside hydrolase	26967-27938	669	223					
sll5	MFS General Substrate Transporter	27995-30525	1086	361					
sll4	PLAC8-domain containing protein	31922-32704	477	158	*stl4*	PLAC8 domain protein	2585-3357	390	129
sll3	Hypothetical protein	33106-36029	2574	857	*stl3*	Hypothetical protein	3672-6881	2781	926
sll2	**Alcohol dehydrogenase**	**38234-39627**	**1008**	**335**	***stl2***	**Alcohol dehydrogenase**	**9186-10578**	**1023**	**340**
sll1	**O-fucosyl transferase**	**39928-41527**	**1500**	**499**	***stl1***	**Hypothetical protein**	**10827-12489**	**1497**	**498**
slpks1	**PKS**	**42484-52449**	**8448**	**2815**	***stpks1***	**PKS**	**13165-23119**	**8475**	**2824**
slr1	**Hypothetical Protein**	**54641-56527**	**1215**	**404**	***str1***	**Hypothetical protein**	**27594-28370**	**624**	**207**
slr2	**S-Adomet dependent methyltranferase**	**56897-58091**	**810**	**269**	***str2***	**S-Adomet dependent MeT 1**	**28770-29980**	**828**	**275**
slr3	**S-Adomet dependent methyltranferase**	**58455-60222**	**999**	**332**	***str3***	**S-Adomet dependent MeT 2**	**35252-35276**	**864**	**287**
slr4	**GMC-oxidoreductase**	**60809-63409**	**1801**	**600**	***str4***	**GMC Oxidoreductase**	**37771-37792**	**1899**	**632**

slr5	**Hydrolase**	**63664-65658**	**1893**	**630**	***str5***	**Hydrolase 1**	**39540-40659**	**837**	**279**
					str6	**Hydrolase 2**		**915**	**305**
slr7	**Aldoketoreductase**	**65974-67343**	**1011**	**336**	***str7***	**Aldoketoreductase**	**40980-**	**1011**	**336**
slr8	**Non-heme iron oxidase**	**70875-72238**	**1251**	**416**	***str8***	**Non heme iron oxidase**	**45119-46512**	**1278**	**425**
slr9	**Salicylate hydroxylase**	**72349-74289**	**1359**	**452**	***str9***	**Hydroxylase**	**46883-48503**	**1187**	**395**
slr10	**CoA-ligase**	**75250-78210**	**1730**	**576**	***str10***	**CoA-Ligase**	**49862-52629**	**1914**	**637**
slr11	**PAL**	**79654-83790**	**2976**	**991**	***str11***	**PAL**	**53967-56595**	**2109**	**702**
slr12	Anthranilate synthase	84332-85941	1542	513	*str12*	Anthranilate synthase	58620-61547	2106	701
slr13	Hypothetical Protein	86663-87367	402	133	-	-	-	-	-
slr14	Copper radical oxidase	87705-89665	1410	470	*str14*	Copper radical oxidase	61792-64060	1728	575
slr15	Hypothetical Protein	89890-92045	1017	338	-	-	-	-	-
slr16	DNA mistmatch protein	92051-94590	1659	552	*str16*	DNA mismach protein	67840-75582	2064	687
slr17	Cell Morphogenesis	95237-102979	7260	2419	*str17*	Cell morphogenesis	67840-75582	7047	2348
slr18	Hypothetical protein	103283-103895	480	160	*str18*	Hypothetical Protein	77672-78545	389	129
slr19	Hypothetical protein	105239-105807	510	169	*str19*	WD40 repeat like protein	78764-79858		
					str20	MFS general substrate transporter	80239-82097	1725	574
					str21	Hypothetical protein	82398-84665	1345	448
					str22	Polynucleotide 5-hydroxyl kinase	84936-87284	2251	750
					str23	Hypothetical protein	87973-90004	1340	446
					str24	Hypothetical protein	90121-92566	2293	764
					str25	Hypothetical protein	98096-100316	1239	412
					str26	Hypothetical protein	101697-103020	978	325
					str27	Hypothetical protein	104544-108093	2694	897
					str28	Hypothetical protein	110077-111170	720	239
					str29	Hypothetical protein	112420-117150	3453	1150
					str30	N-alpha acetyltranferase	117477-120728	2295	764
					str31	Triphosphatase	121274-121832	258	85
					str32	Kinase	122086-121832	2394	797
					str33	S-Adomet dependent methyltransferase	131005-132003	897	299
					str34	Hypothetical Protein	132814-235653	2718	905
					str35	Desaturase	135862-137143	1146	381
					str36	Oxidase	137301-138446	840	279
					str37	Hydrolase	139089-140287	646	215
					str38	Hypothetical protein	144872-145691	621	206
					str38	A/B/C/D cyclin	147331-149204	1644	547
					str39	Hypothetical protein	158610-159636	735	244
					str40	Hypothetical protein	160119-160682	564	187
					str41	Peptidase	161952-163391	624	207
					str42	FAD dependent oxidoreductase	166431-168103	1308	435
					str43	L-ornithine transportase	168242-170572	770	256
					str44	Cytochrome P450	170582-172075	1270	422
					str45	Aldoketoreductase	175196-177452	1059	353
					str46	Alcohol dehydrogenase	177532-179162	1038	345

Supplementary Table 6. Primer Sequences

Oligo Name	Sequence 5' to 3'
**ITS**	
ITS 4	TCCTCCGCTTATTGATATGC
ITS 5	GGAAGTAAAAGTCGTAACAAGG
**Amplification of missing sequence between contigs 273 and 195**	
C195JoinF2	TCTCCAATAAGCGACGCATGCTACTACTC
C273JoinR2	TCCGAGATGGCCCGTGAGGATATGGATTGG
stpks1	
stpks F4	ATGTCACCTACTGCTGAAATTCC
stpks F4t	ATGCCAACTTTGTACAAAAAAGCAGGCTCCATGTCACCTACTGCTGAAATTCC
stpks R4	CGGGTAAGATAGGTTCACCT
stpks F3	GATCTCGCTGAGCACGTCAT
stpks R3	GAGCCATGGGTTTTGAGCGA
stpks F2	AAGATCCAGAACAAGGTGCC
stpks R2A	CCACCATGTTCTTGTAGGAG
stpks F1B	TGGTTTGACGAAGTTCCTCGT
stpks R1t	TATAATGCCAACTTTGTACAAGAAAGCTGGTTAGACCTTCTCTGCTTCGAACA
str8	
str8 R2	CCTATCGCAGCATCCTCCCTCT
str8 F3	TCTCCATTGCCTTCGCAATC
str8 R1	AATGCCCTTCTTGCATGTAACAC
str8 F1	ATGGTCTACATGTTATTCCCCTCG
str8 R4	ATCTGGGCATCGGCATGCAT
str8 F2	ATGTCTCGCCTGTTGTGCCCTT
str8 R2t	CGACAATGTCCATATCATCAATCATGACCCTATCGCAGCATCCTCCCTCT
str8 F2t	AACAGCTACCCCGCTTGAGCAGACATCACCATGTCTCGCCTGTTGTGCCCTT
str10	
str10 R1	AATCGATCCCGGCTCCAAGGTGCTT
str10 F2	ATGCAGAATGGGTGCGGCCTAGTC
str10 R2t	AGGTTGGCTGGTAGACGTCATATAATCATAATTGTCTACGATCGGTCGCG
str10 F4	AAGGTCTCCTACAAGCACCT
str10 R5	TGCTCGGTGCAAGCAAACGA
str10 Ft	TCGACTGACCAATTCCGCAGCTCGTCAAAGATGACCATCCTCCGTTCTCG
str11a	
str11 F2t	TTTCTTTCAACACAAGATCCCAAAGTCAAAATGCCATACCCGTCTGACTT
str11 R1	AATCAACGAGACGTTCTGGC
str11 F5	GTGACGATTGGCCAGAACGT
str11 R2t	TTCATTCTATGCGTTATGAACATGTTCCCTCAAGCAAATAGTCCAACGAC
stl2	
stl2-Ft	TTTCTTTCAACACAAGATCCCAAAGTCAAAATGCCTGAAGGAACCATGAA
stl2 R1t	GGTTGGCTGGTAGACGTCATATAATCATACTTAGGGCTTGATCGCAATTTTCAAGGC
str2	
*str2*F1	ATGGCCGCCGAATCTGCTAA
*str2*R1	TTAGGCACTCTTCTTCACCG
*str2*F1 tail	CCTTTCTTTCAACACAAGATCCCAAAGTCAAAATGGCCGCCGAATCTGCTAA
*str2*R1 tail	TCATTCTATGCGTTATGAACATGTTCCCTGTTAGGCACTCTTCTTCACCG
str3	
*str3*F1	ATGTCTTCTCCTGCTGCTCA
*str3*R1	TTAGACACGCTTCTGCGACC
*str3*F1 tail	AACAGCTACCCCGCTTGAGCAGACATCACCATGTCTTCTCCTGCTGCTCA
*str3*R1 tail	ACGACAATGTCCATATCATCAATCATGACCTTAGACACGCTTCTGCGACC
str4	
*str4*F1	ATGGGAGCCTCGCACTCGACAGTCT
*str4*R1	TTATGCGACGCCCTTGATGAG
*str4*F1 tail	GTCGACTGACCAATTCCGCAGCTCGTCAAAATGGGAGCCTCGCACTCGACAGTCT
*str4*R1 tail	GGTTGGCTGGTAGACGTCATATAATCATACTTATGCGACGCCCTTGATGAG
str9	

str9-Ft	TCTGAACAATAAACCCCACAGCAAGCTCCGATGGCCGTTGATCGCAAGAT
str9-Rt	CTCTCCACCCTTCACGAGCTACTACAGATCCTAGACAAGGCGTTCCTCAA
*str9*_padh_F	TTTCTTTCAACACAAGATCCCAAAGTCAAAATGGCCGTTGATCGCAAGAT
*str9*_teno_R	GGTTGGCTGGTAGACGTCATATAATCATACCTAGACAAGGCGTTCCTCAA
**Patch-adh**	
ExAdhSeq.F	GTAAATCAACCCTAACTCCA
ExAdhSeq.R	GAGGAAGAGACGATGGAATC
**Patch-gpd**	
ExGpdSeq.F1	CCTTTCAGTTCGAGCTTTCC
ExGpdSeq.R1	TACAATTAAGCTCTTCATTT
**Patch-amyB**	
stpks R4	CGGGTAAGATAGGTTCACCT
PamyB F1	CCTTGTCGATGCGATGTATC
**Patch CmR**	
CmR F1	GAATTGGATATCACAAGTTTGT
CmR R1	TAACAAGGGTGAACACTATC
**Patch Remove**	(For construction of pTYGS-ARG-stPKS)
P1136_R	GCACGTTCCTTATATGTAGCT
P1137_F	TCCTTTCCCTCTCTCCGGTGACTCGTTTTCGCAGTTCCCTACTCTCGCGT
	Oligonucleotides for cloning plasmid pTYGS-NiaD-*str2*-*str*
str2_F_padH	TTTCTTTCAACACAAGATCCCAAAGTCAAAATGTCTTCTCCTGCTGCTCA
str2_R_TadH	TTCATTCTATGCGTTATGAACATGTTCCCTTTAGACACGCTTCTGCGACC
str3_F_pgpdA	TAACAGCTACCCCGCTTGAGCAGACATCACATGGCCGCCGAATCTGCTAA
str3_R_TgpdA	ACGACAATGTCCATATCATCAATCATGACCTTAGGCACTCTTCTTCACCG

Supplementary Table 7. Vector combinations for heterologous expression experiments. Table positions refer to entries in Table 2 in the main paper.

Vector	pTAGS-Arg-*stpks1*	pTYGS-Arg-*stpks1+str2+str3+str4*	pTYGS-Ade-*str10*	pTYGS-Ade-*stpks1+str8+str10*	pTYGS-Ade-*str8+str10+str11*	pTYGS-Met-*str9+stl2*	pTYGS-Met-*str9*	pTYGS-NiaD-*str2-str3*
**Table Position**	**Arginine Selection**		**Adenine Selection**			**Methionine Selection**		**Nitrate Selection**
2-1	✓							
2-2	✓							
2-3	✓							
2-4	✓		✓					
2-5	✓		✓					
2-6				✓				
2-7	✓							
2-8				✓				
2-9				✓				
2-10	✓				✓			
2-11		✓						
2-12		✓			✓			
2-13		✓			✓		✓	
2-14		✓			✓	✓		
2-15	✓				✓		✓	
2-16	✓				✓	✓		
2-17	✓				✓		✓	✓
2-18	✓				✓	✓		✓

Supplementary Table 8. blastp analysis of predicted proteins encoded by ORFs located within type 1 introns of the cytochrome b genes of *S. tenacellus* and *S. lutea* (see Supplementary Figures 47 and 48 for protein sequences).

	Strain	Size (a.a)	NCBI homologue	% id.	E value	Domains
Intron1 ORF	*S.ten.*	464	I-AbiIII-cob-P, partial (mitochondrion) *Agaricus bisporus* (AFP72247.1)[16]	55	$7e^{-125}$	pfam00961, pfam14528
	S.lutea	477		55	$5e^{-121}$	
Intron2 ORF	*S.ten.*	320	*Neurospora crassa* cytochrome b mRNA maturase bI3 (P0CY43.1)[17]	59	$1e^{-114}$	-
	S.lutea	318		59	$4e^{-114}$	
Intron3 ORF	*S.ten.*	317	*Aspergillus nidulans* I-AniI maturase (P03880.3)[13]	48	$3e^{-96}$	pfam00961, pfam00961
	S.lutea	317		50	$5e^{-106}$	
Intron4 ORF	*S.ten.*	228	LAGLIDADG endonuclease from *Podospora curvicolla* (CAB72448.1)[18]	42	$1e^{-53}$	pfam03161
	S.lutea	231		43	$5e^{-61}$	

Supplementary Methods

gDNA Preparation and Genome Sequencing

S. tenacellus and *S. lutea* gDNA were extracted from frozen mycelia using chemical lysis.[5] Genomic DNA was then purified using equilibrium centrifugation in CsCl-ethidium bromide gradients.[6] Analysis by gel electrophoresis showed good quality gDNA, visualised as a compact, high molecular weight band (Supplementary Figure 1). The gDNA was sequenced using an Illumina HiSeq2500 system at the University of Bristol Sequencing Facility. GC content is 53.6% for *S. tenacellus* and 52% for *S. lutea* F23523 (Supplementary Table 1). The sequence for each species was assembled using the De Novo Assembly tool of CLC Genomic Workbench (Supplementary Table 2).

Identification of PKS Gene Clusters *in silico*

The assembled genomes of *S. tenacellus* and *S. lutea* F23523 were submitted to antiSMASH,[7] which identified twenty-eight putative biosynthetic gene clusters (BGC) for *S. lutea* F23523 and twenty-nine putative gene clusters for *S. tenacellus* (Supplementary table 3).

In addition to using antiSMASH, a BLAST-searchable Bio-Linux[8] database was created and used to determine whether there were any additional polyketide gene clusters. A PKS from contig Sl-298 of *S. lutea* F23523 was used as a query to search for other polyketide gene clusters. Only contigs Sl-1078, St-2 and St-273 contained putative polyketide encoding genes with significant homology to the query (*E*-value < 0.05), which matched those identified using antiSMASH.

Contigs St-2 and St-273 from *S. tenacellus* and contigs Sl-298 and Sl-1078 from *S. lutea* F23523 were further analyzed to identify the position of the *pks* gene and potential surrounding tailoring genes. Each cluster was manually translated *in silico* using the FGENESH program from Softberry,[9] using parameters based on the model basidiomycete *Coprinopsis*

cinerea. Each predicted protein was then submitted to protein BLAST (blastp) to predict the functions of the encoded protein. The lack of genes encoding certain tailoring enzymes such as methyltransferases made these clusters poor candidates for strobilurin biosynthesis.

In contrast to the PKS BGCs found on contigs St-2 and Sl-1078, the PKS BGC found on contig Sl-298 contained various interesting genes. These genes include *slr9*, *slr10*, and *sll2* which encode a ligase, a PAL, and an alcohol dehydrogenase respectively (Supplementary Table 5). The presence of such genes made this cluster a strong candidate for strobilurin biosynthesis in *S. lutea* F23523.

Some genes from contig St-273 of *S. tenacellus* were predicted to be homologous to genes within contig Sl-298 of *S. lutea* F23523 (Supplementary Table 5). However, contig St-273 does not contain the ligase, PAL, GMC oxidoreductase or aldoketoreductase encoding genes. To investigate whether contig Sl-298 of *S. lutea* F23523 and contig St-273 of *S. tenacellus* are actually homologous, further investigations of contig St-273 were carried out by bioinformatic analysis and PCR.

Identifying an adjacent contig to contig 273 *S. tenacellus*

The genes encoding the PAL, GMC oxidoreductase, aldoketoreductase, and acetyl-CoA ligase from contig Sl-298 of *S. lutea* F23523 were used to search the genome data of *S. tenacellus*. Homologues of all of these genes were identifed on contig St-195.

To determine whether contigs St-273 and St-195 represent neighbouring fragments of the genome a pair of primers (C195JoinF2 and C273JoinR2) were designed to bind within each contig and amplify any missing sequence (Supplementary Figure 2). A PCR product of around 4 kb was generated (Supplementary Figure 3), showing that the two contigs are adjacent.

The PCR product was ligated into a blunt cloning vector (pJET 1.2), transformed into *E. coli* (TOP10, ThermoFisher), and the resulting plasmid was isolated from separate colonies. A restriction digest analysis was then conducted using two different combinations of enzymes (*Pst*I/*Not*1 and *Pst*1/*Xba*I) to confirm the presence of the insert. The restriction patterns were predicted, based on the known *S. lutea* sequence, to include 4 bands (368, 615, 2641 and 2792 bp) for *Pst*I/*Xba*1 and 3 bands (309, 3302 and 3633 bp) for *Pst*I/*Not*1 (Supplementary Figure 4). The restriction pattern of all plasmids were similar to the prediction, suggesting that the correct fragment was amplified and cloned.

Four of the cloned PCR products were sequenced and the length of each was found to be 3481 bp. Alignment of the resulting sequences showed some single nucleotide

polymorphisms (SNPs) demonstrating the dikaryotic nature of *S. tenacellus*. The sequences were assembled along with contigs Sl-195 and St-273 using Sequencher Software.[10] The assembly conclusively demonstrated that the two contigs (St-195 and St-273) are adjacent in the genome, and they represent one gene cluster. The total sequence of both contigs was 179254 bp (renamed contig St-195+273). This sequence was translated *in silico* using FGENESH from Softberry with *C. cinerea* as a model, and homologues within the NCBI database were identified using the blastp algorithm to assign putative function. The results were visualized and annotated using Artemis.[11]

Transcriptomic Analysis

Annotation of contig St-195+273 from *S. tenacellus* was revised using transcriptomic sequence data. *S. tenacellus* mycelium prepared from strobilurin non-producing (YM media, 6 days) and producing conditions (CGC medium, 6 days) was used to isolate mRNA. Both conditions were verified by LCMS and ^{1}H-NMR. RNA was isolated using an RNA isolation kit (ThermoFisher Scientific) and cDNA libraries were constructed using the TruSeq RNA-sequencing kit v2 (Illumina). The libraries were submitted for sequencing at the University of Bristol Genomic Facility using a HiSeq 2500 instrument, with a paired end 2 x 100 bp run type. The resulting RNA data for each condition were mapped to the *S. tenacellus* genome using Galaxy software. The data were visualized using Artemis for further analysis.[12] Normalisation was performed by calculating the RPKM (reads per kilobase per million reads mapped to the genome), which takes into account both the gene length and the total library size.

The re-annotation of contig St-195+273 focused only on the region from *stl2* to *str11* which was predicted to be involved in strobilurin biosynthesis (the strobilurin biosynthetic gene cluster [SBGC] in *S. tenacellus*). Miscalled introns in almost all of the genes were revised using the RNA-seq data. Start and stop codons were also revised where necessary.

A hydrolase encoded by *slr5* and *str5* was identified from genomic sequence, with the initial annotation suggesting that *str5* was much longer than *slr5* (Supplementary Figure 5). Surprisingly, the transcriptomic data revealed that the gene cluster from *S. tenacellus* actually contains 2 separate genes both encoding hydrolases, which have now been named *str5* (885 bp) and *str6* (1120 bp). The two genes were separated by 596 bp (Supplementary Figure 5). *str5* is homologous to the N-terminus of *slr5* from *S. lutea* F23523 and *str6* is homologous to the C-terminus of *slr5*.

Contig 298 of *S. lutea* F23523 was also re-annotated by sequence comparison with the revised annotation of the predicted SBGC of *S. tenacellus*. Some of the genes showed the same

intron positions such as *sll2, sll1, slpks1, slr7 slr10*. The gene *slr11* showed some different intron positions due to a different amino acid sequence from its homologue *str11*. The predicted SBGC of *S. lutea* F23523 has a slightly different gene organization from the predicted SBGC of *S. tenacellus*. *S. lutea* contains only one gene encoding a hydrolase (SLR5) whilst the predicted SBGC of *S. tenacellus* contains 2 hydrolases (STR5 and STR6, Supplementary Figure 6, Supplementary Table 5). However, all other homologues from the cluster showed over 75% identity.

The expression level of each gene from the Strobilurin BGC of *S. tenacellus* was analyzed from transcriptomic data. The expression level of each gene was counted using Artemis as:

$$Expression\ level\ of\ gene = \frac{read\ count\ total\ without\ intron\ from\ sense\ and\ antisense}{gene\ length\ (kb)}$$

From the 17 genes surrounding *stpks1*, some showed high levels of expression under producing conditions compared with non-producing conditions. These were *stl2*, *stpks1*, *str1*, *str2*, *str3*, *str4*, *str7*, and *str9* (Supplementary Figure 7). The genes *str6*, *str8*, *str10*, and *str11* had low level gene expression under production conditions whilst *stl1* showed the same level gene expression under both conditions.

Confirmation of Identity of the Strains Producing Strobilurins

Genomic DNA (gDNA) of *S. tenacellus* and *B. lutea* F23523 was isolated using the GenEluteTM Plant Genomic DNA Miniprep Kit (Sigma). The universal primers, ITS4 and ITS5, were used to amplify the ITS region. The amplified PCR product in both cases was approximately 800 bp. The product was purified and directly sequenced (Beckman Coulter Genomics). The resulting ITS sequences were aligned with publicly available ITS sequences from a range of *Strobilurus* strains as well as other basidiomycete species and some Ascomycete species. A phylogenetic tree was then constructed in MEGA4[13] using the neighbour-joining method. As expected, the sequence generated from *S. tenacellus* clusters with the sequences of other *Strobilurus* species. A slightly unexpected result was that *B. lutea* F23523 also falls within the *Strobilurus* species clade (Supplementary Figure 8). This demonstrates that *B. lutea* F23523 has been incorrectly identified as an Ascomycete and is in fact a Basidiomycete, likely belonging to the *Strobilurus* genus. It was therefore re-named *Strobilurus lutea* F23523.

PKS Domain Analysis

Domains within the predicted protein stpks1 were initially identified by searching against the conserved domain database (CDD) of NCBI. This identified a total of 9 putative catalytic domains including a β-ketoacylsynthase (KS), acyltransferase (AT), dehydratase (DH), methyltransferase (MeT), ketoreductase (KR), acyl carrier protein (ACP), hydrolase and a C-terminus methyltransferase (Supplementary Figure 9). StPKS1 was also aligned with other known sequences (Supplementary Figure 10).

BLAST analysis of Unknown Domain

The unknown domain of StPKS1 was blasted against the NCBI non-redundant (nr) protein database in isolation. Two proteins were shown to have shown to have significant homology (E-value ≤ 0.05); a polyketide synthase from *Stereum hirsutum*, and a ketoacyl-synthase-domain-containing protein from *Gloeophyllum trabeum*. Both of these strains are Basidiomycetes, but are not closely related to the *Strobilurus* genus, belonging to different orders of Basidiomycetes. This demonstrates that similar protein sequences are relatively rare, but do exist within PKS enzymes or similar predicted proteins (Supplementary Figure 11).

Transcription analysis of *stpks1*

Transcriptomic data obtained during the production of strobilurin A **1** by *S. tenacellus* were mapped to genomic sequence containing *stpks1* and displayed using Artemis. The results show that the sequence downstream of the *stpks1* ACP forms part of a single transcribed CDS (Supplementary Figure 12).

Construction of *A. oryzae* Expression Plasmids

pTAGS-*stpks1*

The *stpks1* gene was amplified from cDNA using 4 pairs of primers (*stpks* F4t/*stpks* R4, *stpks* F3/*stpks* R3, *stpks* F2/*stpks* R2A, *stpks* F1B/*stpks* R1t) to generate 4 fragments with sizes of approximately 3, 3, 3, and 0.6 kb, respectively. The fragments were reassembled by yeast recombination into a cloning vector, pEYA, using *Saccharomyces cerevisae*. The coding sequence of *stpks1* was then transferred from pEYA-*stpks1* into the expression vector pTAGS using GatewayLR *in vitro* recombination to produce pTAGS-*stpks1* (Supplementary Figure 13).

pTAGS-*stpks1* was propagated in *E. coli* and the resulting colonies were screened by colony PCR with primers *stpks* F4 and *stpks* R4. Five colonies produced PCR products with the correct size (approximately 3 kb, Supplementary Figure 14). Two of the positive colonies were cultured in

LB medium and the plasmids were isolated. Correct construction was confirmed by restriction digest analysis using *Bam*HI (2645, 6776 and 6780 bp fragments) and *Nco*I (1732, 2430, 5138 and 6901 bp fragments), as well as sequencing.

pTYGS-Ade-*str8*+*str10*+*str11*

Str8, *str10* and *str11* were amplified from the cDNA of *S. tenacellus* grown under production conditions. Due to difficulties in obtaining long RT-PCR products, in some cases full length coding sequence was generated by amplifying and reassembling overlapping fragments.

Amplification of the PCR product for *str8* was carried out using 3 primer pairs. *Str8* R2/*str8* F3, *str8* R1/*str8* F1, and *st8* R4/*str8* F2 produced fragments of 422 bp, 1,002 bp and 230 bp respectively (Supplementary Figure 15). Each fragment was ligated into the cloning vector, pJET1.2, and confirmed by sequencing (Beckman Coulter Genomics). The fragments of *str8* were then re-amplified from clones, introducing tails to the 5' end of fragment 1 and the 3' end of fragment 3 by using primers *str8* R2t and *str8* F2t. The introduced tails were homologous to the expression vector, pTYGS-Ade,[14] which allowed *str8* to be reconstructed directly into the expression vector by yeast recombination.

The amplification of *str10* was achieved using *str10* F2/ *str10* R1 from cDNA, while *str10* F4/ *str10* R2t and *str10* Ft/*str10* R5 successfully amplified the intron-free fragments 1 and 3 from gDNA respectively (Supplementary Figure 16). Tails were added to the 5' end of fragment 1 and the 3' end of fragment 3 which were homologous to the expression vector, pTYGS-Ade.

The majority of *str11* was amplified successfully using just one pair of primers; *str11* F2t/*str11* R1 (Supplementary Figure 17), to give fragment 1. A short portion at the 3' end of the gene (fragment 2) was synthesized by Life Technology and assembled into vector pMA-RQ (ampR). Fragment 2 was then amplified using primers *str11* F5 and *str11* R2t (Supplementary Figure 17). As with previous genes, tails were added to either end of the gene by using compound primers, which allowed direct recombination into pTYGS-Ade.

Recombination of all fragments for *str8*, *str10*, and *str11* into pTYGS-Ade was conducted in *S. cerevisiae* (Supplementary Figure 18). pTYGS-Ade was digested with *Asc*I to allow the insertion of the three genes. Yeast recombination placed *str10* between the enolase promoter (P_{eno}) and terminator (T_{eno}), *str8* between the gpdA promoter (P_{gpdA}) and terminator (T_{gpdA}), and *str11* between the alcohol dehydrogenase promoter (P_{adh}) and terminator (T_{adh}). The recombinant plasmid (pTYGS-Ade-*str8*+*str10*+*str11*) was isolated from *S. cerevisiae* and propagated in *E. coli*. The recombinant plasmid was screened using *Eco*RV to identify the

correct fragment pattern (317, 1711, 3533, 5265, 9418 bp, Supplementary Figure 18). The original plasmid was also cut with *Eco*RV to differentiate between the recombinant plasmid and the original plasmid (1711, 3533, 9801 bp). From this it can be concluded that pTYGS-Ade-*str8*, *str10*, and *str11* was constructed correctly.

pTYGS-Ade-*stpks1+str8+str10*

pTYGS-Ade-*stpks1+str8+str10* was constructed in two steps. Firstly, *stpks1* was transferred from pTAGS-*stpks1* to pTYGS-Ade-*str8+str10+str11* using Gateway™ Recombination (Supplementary Figure 19). Resulting *E. coli* colonies were screened for the presence of *stpks1* by colony PCR using the primer pair *stpks* F4/ *stpks* R4. Two of five colonies were positive, producing a band of the correct size (Supplementary Figure 20). From a positive colony, the plasmid pTYGS-Ade-*stpks1+str8+str10+str11* was isolated.

The second step was deletion of *str11* from pTYGS-Ade-*stpks1+str8+str10+str11*. pTYGS-Ade-*stpks1+str8+str10+str11* was cut using *Not*I which generated 2 breaks, one within the *PamyB*/*stpks1* cassette and one within *str11*. A region of *PamyB* and *stpks1* (Patch-amyB) was amplified using the primers *stpks* R4 and PamyB F1 to allow the repair of the break within *stpks1* during yeast recombination. A second fragment, consisting of a portion of an empty *adh* cassette from pTYGS-Ade (Patch-adh:148 bp), was amplified with primers ExAdhSeq.F and ExAdhSeq.R. Recombination of this fragment with the digested pTYGS-Ade-*stpks1+str8+str10+str11* served to remove *str11* (Supplementary Figure 21). The various fragments were transformed into *S. cerevisiae* and the resulting reassembled plasmid was then propagated in, and isolated from, *E. coli.*

The resulting *E. coli* colonies were screened by colony PCR with primers ExAdhSeq.F and ExAdhSeq.R (Supplementary Figure 22). The resulting plasmid, pTYGS-Ade-*stpks1+str8+str10*, was isolated for heterologous expression in *A. oryzae*

pTYGS-Ade-*str10+str11*

Construction of pTYGS-Ade-*str10+str11* (lacking *str8*) was achieved by deletion of *str8* from pTYGS-Ade-*str8+str10+str11* (Supplementary Figure 23). pTYGS-Ade-*str8+str10+str11* was digested using *Eco*RI, which produced 2 breaks; in the CmR and *str8* regions. The first gap located in CmR was repaired with the DNA fragment, "Patch-CmR", amplified from pTYGS-Ade-*str8+str10+str11* using primers CmR F1 and CmR R1. The second gap was fixed using "Patch-gpd" to remove *str8*. Patch-gpd (148 bp) was a short DNA fragment amplified from pTYGS-Ade by PCR using primers ExGpdSeq.F1 and ExGpdSeq.R1.

Cut pTYGS-Ade-*str8*+*str10*+*str11* was assembled with Patch-CmR and Patch-gpd by yeast homologous recombination (Supplementary Figure 23). The assembled plasmid was isolated from *S. cerevisiae* and transformed into *E. coli*. *E. coli* colonies were screened by colony PCR using primers ExGpdSeq F1 and ExGpdSeq R1. A colony which gave the expected product was used to isolate the plasmid, which was then analyzed by restriction digest analysis (Supplementary Figure 23).

pTYGS-Ade-*str10*

pTYGS-Ade-*str8*+*str10*+*str11* was cut with a mixture of *Eco*RI and *Not*I which produces 3 breaks; in the CmR, *str8*, and *str11* regions (Supplementary figure 24). The CmR break was repaired with Patch-CmR. The break within *str8* was fixed using Patch-gpd to remove *str8*. The third break within *str11* was closed with Patch-adh which removed *str11*. Digested pTYGS-Ade-*str8*+*str10*+*str11* was recombined with Patch-CmR, Patch-gpd, and Patch-adh by yeast recombination. The resulting plasmid was isolated from *S. cerevisiae* and propagated in *E. coli*. Restriction digest analysis using *Nde*I was used to confirm correct construction of pTYGS-Ade-*str10* (Supplementary Figure 25).

pTYGS-Met-*str9*+*stl2*

pTYGS-Met[14] was fully digested with *Asc*I and *Not*I to create breaks within all four of the promoter/terminator cassettes (*amyB*, *adh*, *gpd* and *eno*). The genes *stl2* and *str9* were amplified from cDNA using the primer pairs stl2-Ft/stl2R1t and str9-Ft/str9-Rt respectively. *Stl2* was amplified to introduce tails homologous to P_{adh} and T_{eno}. *Str9* was amplified with tails homologous to the *amyB* cassette. These fragments (*stl2*, *str9*, and Patch-Padh) were recombined into pTYGS-Met by yeast recombination to produce the plasmid pTYGS-Met-*str9*+*stl2* (Supplementary Figure 26). pTYGS-Met-*str9*+*stl2* was isolated from *S. cerevisiae* and propagated in *E. coli*. Confirmation of the correct construction was carried out by restriction digest analysis using *Nco*I.

pTYGS-Met-*str9*

The vector pTYGS-met was digested using *Asc*I to create breaks between the promoter and terminator sequences of the *gpd*, *adh* and *eno* cassettes. The intron-free gene *str9* was amplified from expression vector pTYGS-met-*str9*-*stl2* using oligonucleotides str9_padh_F and str9_teno_R and recombined into pTYGS-met between P_{adh} and T_{eno} using yeast recombination. The plasmid was purified from *S. cerevisiae* and propagated in ccdB survival™ cells

(ThermoFisher). Confirmation of correct construction was carried out by restriction digest analysis using *Sal*I (Supplementary Figure 28). Furthermore, sequencing of *str9* confirmed that str9 was cloned without introducing any mutations.

pTYGS-Arg-*stpks1+str2+str3+str4*

The tailoring genes were constructed in derivatives of the expression vector pTYGS for expression in *A. oryzae* NSAR1. *Str2*, *str3, and str4* were amplified successfully by PCR from cDNA, adding tails to the 5' and 3' ends of each gene to allow recombination into the expression vectors.

pTYGS-Arg[14] was cut with *Asc*I to produce breaks within the three cassettes where *str2*, *str3* and *str4* would be inserted (P_{adh}/T_{adh}, P_{gpdA}/T_{gpdA}, and P_{eno}/T_{eno}, respectively), and the digested plasmid was combined with the amplified coding sequences in yeast (Supplementary Figure 29). The resulting recombinant plasmid (pTYGS-Arg-*str2+str3+str4*) was isolated from *S. cerevisiae* and propagated in *E. coli* (ccdB survival™ cells). The recombinant plasmid was digested using *Xba*I to confirm correct construction (Supplementary Figure 29).

The vector pTYGS-Arg-*str2+str3+str4* was then used in an LR Clonase™ gateway reaction, to transfer *stpks1* from pEYA-*stpks1* into the spare expression cassette in the vector to produce pTYGS-Arg-*stpks1+str2+str3+str4*. *E. coli* colonies were initially screened by colony PCR using primers *stpks1* F4 and *stpks1* R4. The resulting plasmid was digested using *Xba*I (predicted fragment pattern: 13049, 5035, 4452, 3576, 131 bp). The smallest fragment size of 131 bp was too small to be visable using our gel electrophoresis methology, but all other predicted bands were correct for both pTYGS-Arg-*stpks1+str2+str3+str4* and the control plasmid pTYGS-Arg, implying correct construction (Supplementary Figure 30).

pTYGS-NiaD-*str2-str3*

The vector pTYGS-NiaD was digested using *Asc*I to create breaks between the promoter and terminator sequences of the *gpd*, *adh* and *eno* cassettes. The intron-free genes *str2* and *str3* were amplified from expression vector pTYGS-ARG-*stpks1+str2+str3+str4* using oligonucleotides str2_F_padh + str2_R_tadh and str3_F_pgpdA + str3_R_tgpdA. Together with Patch Peno (prepared from amplification of a region of pTYGS-arg using primers Patch_Peno_F and Patch_Peno_R to rejoin the *eno* promoter and terminator fragments.) the amplified gene fragments str2 and str3 were recombined into pTYGS-niaD using yeast recombination. The plasmid was purified from *S. cerevisiae* and propagated in ccdB survival cells (ThermoFisher). Confirmation of correct construction was carried out by PCR.

pTYGS-Arg-*stpks1*

The vector pTYGS-Arg-*stpks1+str2+str3+str4* was digested using *Nde*I to remove the tailoring genes *str2, str3* and *str4*. A patch was amplified from the empty pTYGS-Arg plasmid using oligonucleotides P1137_F and P1136_R and recombined using yeast recombination. The plasmid was purified from *S. cerevisiae* and propagated in *E. coli* TOP10 cells (ThermoFisher). Confirmation of correct construction was carried out by sequence analysis.

Coexpression of *stpks1* and *str10* (PKS + CoA ligase).

pTAGS-Arg-*stpks1* and pTYGS-Ade-*str10* were transformed into *A. oryzae* NSAR1 and selected on minimal media plus methionine and ammonium nitrate. Two transformants were isolated and grown in CMP liquid media. The cultures were fermented in the presence or absence of benzoic acid. After fermentation the cultures were extracted with EtOAc. The organic extracts were concentrated, dissolved in HPLC grade methanol and examined by LCMS. Prestrobilurin **11** A was present in the crude extracts of cultures fed with benzoic acid (Supplementary Figure 31).

Coexpression of *stpks1*, *str10 and str8* (PKS + CoA ligase + Non-heme Iron oxygenase).

pTYGS-Ade-*stpks1+str8+str10* was transformed into *A. oryzae* NSAR1 and selected on minimal media plus methionine, arginine and ammonium nitrate. Seven transformants were isolated and grown in CMP liquid media. Screening of transformants was carried out by inoculation of each transformant into CMP medium supplemented with and without 0.1% *w/v* sodium benzoate. None of the transformants inoculated into standard CMP were able to produce prestrobilurin **11**. However, when inoculated into CMP supplemented with 0.1% of sodium benzoate, three tranformants produced prestrobilurin **11** (Supplementary Figure 32). This result proves that *str11* (PAL) is necessary for the supply of the starter unit in the strobilurin biosynthetic pathway. The genes *str8* and *str10* are sufficient to make benzoyl-CoA **16** if the media is supplemented with sodium benzoate, which implies that the CoA ligase encoded by *str10* does indeed activate benzoic acid. The activity of the non-heme iron oxidase encoded by *str8* remains unproven.

Feeding of *trans*-cinnamic acid **17** into transformant *stpks1+str8+str10* lacking PAL was used to confirm whether *str8* and *str10* were sufficient to use exogenously supplied cinnamate to make benzoyl-CoA **16** (Supplementary Figure 33). A *stpks1+str8+str10* transformant was fed with 0.05% of sodium cinnamate. In parallel it was fed with 0.1% sodium benzoate to confirm that STR10 is still active. LCMS analysis of this transformant

indicated a prestrobilurin **11** peak around 17.7 min (Supplementary Figure 33). It can therefore be concluded that *str8* and *str10* together are sufficient to convert cinnamate to benzoyl-CoA **16**, with the cinnamate normally being generated by *str11* (PAL).

Coexpression of *stpks1* and *str10, str8 and str11* (PKS + CoA ligase + Non-heme Iron oxygenase + PAL).

pTAGS-Arg-*stpks1* and pTYGS-Ade-*str10-str8-str11* were transformed into *A. oryzae* NSAR1 and selected on minimal media plus methionine and ammonium nitrate. Eleven transformants were isolated and grown in CMP liquid media. After fermentation the cutures were extracted with EtOAc. The organic extracts were concentrated, dissolved in HPLC grade methanol and examined by LCMS. Four of the extracts showed the presence of prestrobilurin A **11**.

Synthesis of Prestrobilurin A 11

General Experimental Details

Commercially available compounds were used without further purification except where stated. Experiments which included moisture or air sensitive reactions were carried out in flame-dried glassware under a positive pressure of nitrogen using standard syringe/ septa techniques. Anhydrous solvents dichloromethane and tetrahydrofuran were obtained by passing through a modified Grubbs system of alumina columns, manufactured by Anhydrous Engineering. Petroleum ether is of the 40-60 °C boiling point range. Routine monitoring of reactions was performed using precoated Merck-Keiselgel 60 F_{254} aluminium backed T.L.C. plates. The spots were visualised by UV_{254} light, or potassium permanganate. Flash column chromatography was performed using silica gel (40-63 micron, obtained from Fluorochem Ltd.) as the adsorbent and carried out according to the procedure outlined by Still *et al.*[15] Melting points were determined on an Electrothermal IA6301 melting point apparatus and are uncorrected. Infrared (IR) spectra were recorded on a Perkin Elmer Spectrum One FT-IR spectrometer as either a neat solid or liquid. ^{1}H, ^{13}C and CRAPT NMR spectra were recorded as solution in $CDCl_3$ unless stated otherwise. The spectra were recorded on a lambda 300 MHz, varian 400 MHz or a Jeol Eclipse 400 MHz spectrometer. The chemical shifts (δ) are reported in parts per million (ppm) and the coupling constants (*J*) are in Hertz (Hz). Electrospray (ESI) mass spectra were recorded on a Bruker Daltonics Apex 4e 7.0T FT-MS mass spectrometer. Methane was the ionised gas used for the chemical ionisation. Unless stated, data for all known compounds are in agreement with published data.

3-Methyl-6-phenyl-dihydro-pyran-2,4-dione 12[16]

Diisopropylamine (2.476 mL, 17.668 mmol) was dissolved in anhydrous THF (15 mL) and then cooled to 0 °C under an atmosphere of nitrogen. *n*-BuLi (1.6 M in hexane, 4.418 mL, 7.068 mmol) was added dropwise followed by adding HMPA (1.230 mL, 7.068 mmol) dropwise to the solution. The mixture was cooled to –78 °C and then ethyl 2-methylacetoacetate (1.000 mL, 7.068 mmol) in anhydrous THF (2.2 mL) was added. After the reaction was stirred for 1 h, benzaldehyde (0.790 mL, 7.775 mmol) was added and stirred for 2 h. The reaction was quenched by $HCl_{(aq)}$ (6 M, 8.6 mL) and then allowed to warm to room temperature. The aqueous layer was extracted into Et_2O (3 × 40 mL). The organic layers were combined, dried over $MgSO_4$, filtered and concentrated *in vacuo*. The crude oil was used in the next step without further purification. The crude oil was diluted with $KOH_{(aq)}$ (1 M, 35 mL) and stirred for 14 h. The mixture was cooled to 0 °C and acidified with $HCl_{(aq)}$ (6 M) to pH 2, and a yellow solid was precipitated from the solution. The solid was filtered and washed with water (20 mL). The aqueous layer was extracted with Et_2O (3 × 40 mL). The organic layers were combined, dried over $MgSO_4$, filtered and concentrated *in vacuo*. The yellow solid collected from the filtrate and the concentrated residue was recrystallised from MeOH giving lactone **12** (0.938 g, 65%) as a white solid. m.p. 139-140 °C, lit.[16] m.p. 139 °C; δ_H(400 MHz, DMSO-d$_6$) 1.69 (3H, s, 3-CH_3), 2.64 (1H, dd, *J* 17.0, 3.9, 5-*H*H), 2.87 (1H, ddd, *J* 17.0, 12.0, 1.6, 5-H*H*), 5.41 (1H, dd, *J* 12.0, 4.0, 6-H), 7.31-7.47 (5H, m, ArH); δ_C(100 MHz, DMSO-d$_6$) 8.8 (3-CH_3), 34.7 (C-5), 75.3 (C-6), 97.3 (C-3), 126.4 (Ar), 128.3 (Ar), 128.5 (Ar), 139.3 (Ar), 165.5 and 168.0 (C-2 and C-4). Spectroscopic data in accord with literature.[16]

3-Methyl-6-phenyl-5,6-dihydro-2*H*-pyran-2-one 13

Lactone **12** (0.556 g, 2.722 mmol) was dissolved in anhydrous dichloromethane (13 mL) and cooled to –78 °C under an atmosphere of nitrogen. *N*,*N*-Diisopropylethylamine (0.711 mL, 4.083 mmol) in anhydrous dichloromethane (3.4 mL) was added dropwise and stirred for 20 minutes. Triflic anhydride (0.509 mL, 3.023 mmol) in anhydrous dichloromethane (3.4 mL) was added dropwise over 5 minutes. The reaction was stirred for 30 minutes and then concentrated *in vacuo* giving a residue which was diluted with Et_2O (20 mL). The organic layer was washed with cold $HCl_{(aq)}$ (6 M, 2 × 5 mL) and brine (10 mL). The organic layer was dried over $MgSO_4$, filtered and concentrated *in vacuo*. The crude triflate was dissolved in DMF (14 mL), and then tetrakis(triphenylphosphine)palladium (0.031 g, 0.027 mmol) and triethylsilane (0.870 mL, 5.444 mmol) were added to the solution. The mixture was heated to 60 °C and stirred for 2 h. The mixture was cooled to room temperature and water (10 mL)

was added. The aqueous layer was extracted into EtOAc (3 × 50 mL). The organic layers were combined, extracted into brine (10 mL), dried over $MgSO_4$, filtered and concentrated *in vacuo*. The crude solid was purified by flash chromatography (SiO_2, 20% EtOAc in petroleum ether 40-60 °C) giving lactone **13** (0.492 g, 96% over 2 steps) as a white solid. m.p. 88-90 °C; ν_{max}/cm^{-1} 2985, 1715, 1650, 1449, 1360; δ_H(400 MHz, $CDCl_3$) 2.00 (3H, dt, *J* 2.6, 1.3, 3-CH_3), 2.52-2.71 (2H, m, 5-H_2), 5.43 (1H, dd, *J* 11.9, 4.2, 6-H), 6.68 (1H, ddq, *J* 6.0, 2.8, 1.5, 4-H), 7.33-7.43 (5H, m, ArH); δ_C(100 MHz, $CDCl_3$) 17.1 (3-CH_3), 32.0 (C-5), 79.3 (C-6), 126.0 (Ar), 128.4 (Ar), 128.5 (Ar), 128.8 (C-3), 138.6 (C-4), 138.7 (Ar), 165.7 (C-2); Found (ESI): 211.0729 $[M+Na]^+$, (required $C_{12}H_{12}O_2Na$ 211.0735).

(2*Z*,4*E*)-2-Methyl-5-phenylpenta-2,4-dienoic acid 14

Lactone **13** (0.011 g, 0.056 mmol) was dissolved in anhydrous THF (5 mL) and then TBAF (1 M in THF, 0.28 mL, 0.28 mmol) was added under an atmosphere of nitrogen. The mixture was stirred for 16 h followed by adding water (5 mL). The aqueous layer was extracted with EtOAc (3 × 10 mL). The organic layers were combined, dried over $MgSO_4$, filtered and concentrated *in vacuo*. The crude oil was purified by flash column chromatography (SiO_2, 60% EtOAc in petroleum ether 40-60 °C) giving acid **14** (0.010 g, 91%) as a white solid. m.p. 174-176 °C, δ_H(400 MHz, $(CD_3)_2CO$) 2.02 (3H, s, 2-CH_3), 6.72 (1H, m, 3-H), 6.80 (1H, d, *J* 15.7, 5-H), 7.28 (1H, m, ArH), 7.33-7.39 (2H, m, ArH), 7.48-7.53 (2H, m, ArH), 8.00 (1H, dd, *J* 15.7, 11.2, 4-H); δ_C(100 MHz, $(CD_3)_2CO$) 21.2 (2-CH_3), 127.1 (C-2), 127.6 (C-4), 127.8 (Ar), 129.2 (Ar), 129.6 (Ar), 138.0 (Ar), 138.6 (C-5), 141.1 (C-3), 172.8 (C-1); Found (ESI): 211.0733 $[M+Na]^+$, (required $C_{12}H_{12}O_2Na$ 211.0735).

(2*Z*,4*E*)-2-Methyl-5-phenylpenta-2,4-dien-1-ol 15

Acid **14** (0.053 g, 0.283 mmol) was dissolved in anhydrous THF (1 mL) and was cooled to 0 °C under an atmosphere of nitrogen. Et_3N (0.079 mL, 0.565 mmol) was added dropwise followed by the dropwise addition of ethyl chloroformate (0.035 mL, 0.367 mmol). The solution was stirred at 0 °C for 0.5 h, and then filtered through a plug of Celite. The filtrate was concentrated *in vacuo* and then dissolved in MeOH (2 mL). After cooling the solution to –78 °C, $NaBH_4$ (0.027 g, 0.707 mmol) was added portionwise. The reaction was stirred at –78 °C for 1 h, and then saturated $NH_4Cl_{(aq)}$ (10 mL) was added. The aqueous phase was extracted with EtOAc (3 × 20 mL). The combined organic layers were dried over $MgSO_4$, filtered and concentrated *in vacuo*. The crude product was purified by flash column chromatography (SiO_2, 10% EtOAc in petroleum ether 40-60 °C) giving alcohol **15** (0.036 g, 73%) as a colourless oil.

ν_{max}/cm^{-1} 3300, 3070, 2925, 1655, 1631; δ_H(400 MHz, $CDCl_3$) 1.95 (3H, s, 2-CH_3), 4.36 (2H, s, 1-H_2), 6.12 (1H, d, *J* 11.3, 3-H), 6.50 (1H, d, *J* 15.5, 5-H), 7.06 (1H, dd, *J* 15.5, 11.3, 4-H), 7.22 (1H, m, 9-H), 7.29-7.33 (2H, m, 2 × 8-H), 7.39-7.41 (2H, m, 2 × 7-H); δ_C(100 MHz, $CDCl_3$) 21.9 (2-CH_3), 62.1 (C-1), 124.1 (C-5), 126.4 (C-7), 127.6 (C-8), 128.4 (C-3), 128.7 (C-8), 132.2 (C-5), 137.6 (C-6), 137.8 (C-2); Found (ESI): 197.0945 [M+Na]$^+$, (required $C_{12}H_{14}ONa$ 197.0942).

Methyl (2*E*,4*Z*,6*E*)-4-methyl-7-phenylhepta-2,4,6-trienoate 15a

Alcohol **15** (0.209 g, 1.197 mmol) was dissolved in anhydrous dichloromethane (16.8 mL) at room temperature under an atmosphere of nitrogen. Dess-Martin periodinane (15 wt%, 3.7 mL) added and the mixture stirred for 1.5 h. The reaction was quenched with $NaHCO_{3(aq)}$ (20 mL) and the aqueous phase was extracted with dichloromethane (3 × 40 mL). The organic layers were combined, dried over $MgSO_4$, filtered and concentrated *in vacuo* to give the crude aldehyde as a colourless oil. NaH (60%, 0.067 g, 1.675 mmol) was prewashed by anhydrous hexane (2 × 3 mL) and suspended in anhydrous THF (38.9 mL) under an atmosphere of nitrogen. Trimethyl phosphonoacetate (0.465 mL, 2.872 mmol) was added to the mixture and cooled to 0 °C and stirred for 10 minutes. The crude aldehyde in anhydrous THF (2.9 mL) was added to the reaction dropwise then stirred at room temperature for 16 h. Brine (40 mL) was added and the aqueous layer was extracted into EtOAc (3 × 50 mL). The combined organic layers were dried over $MgSO_4$, filtered and concentrated *in vacuo*. The crude product was purified by flash column chromatography (SiO_2, 10% EtOAc in petroleum ether 40-60 °C) giving ester **15a** (0.165 g, 61% over 2 steps) as a yellow oil. ν_{max}/cm^{-1} 2985, 2933, 1715; δ_H(400 MHz, $CDCl_3$) 1.99 (3H, s, 4-CH_3), 3.80 (3H, s, OCH_3), 5.95 (1H, d, *J* 15.5, 2-H), 6.44 (1H, d, *J* 11.5, 5-H), 6.65 (1H, d, *J* 15.2, 7-H), 7.23-7.29 (2H, m, ArH), 7.31-7.39 (3H, m, ArH and 6-H), 7.47 (1H, m, ArH), 8.03 (1H, d, *J* 15.5, 3-H); δ_C(100 MHz, $CDCl_3$) 20.4 (4-CH_3), 51.8 (OCH_3), 118.1 (C-2), 123.5 (C-6), 127.0 (Ar), 128.3 (Ar), 128.8 (Ar), 132.1 (C-4), 135.6 (C-7), 137.1 (Ar), 137.4 (C-5), 140.6 (C-3), 168.0 (C-1); Found (ESI): 251.1050 [M+Na]$^+$, (required $C_{15}H_{16}O_2Na$ 251.1048).

Prestrobilurin A 11

Ester **15a** (0.032 g, 0.141 mmol) was dissolved in THF (7.3 mL) and $NaOH_{(aq)}$ (1 M, 14.6 mL) added then stirred at room temperature for 16 h. The reaction was cooled to 0 °C and $HCl_{(aq)}$ (6 M) was added to the reaction until pH 2. The aqueous layer was extracted with EtOAc (5 × 50 mL) and the organic layers were combined, dried over $MgSO_4$, filtered and concentrated *in vacuo*. The crude product was purified by flash column chromatography (SiO_2, 60% EtOAc in

petroleum ether 40-60 °C) giving prestrobilurin A **11** (0.028 g, 93%) as a yellow solid. m.p. 166-170 °C; ν_{max}/cm^{-1} 2884, 2566, 1696, 1669, 1617; δ_H(400 MHz, $(CD_3)_2CO$) 2.01 (3H, s, 4-CH_3), 2.90 (1H, br s, OH), 5.96 (1H, d, *J* 15.5, 2-H), 6.55 (1H, d, *J* 11.5, 5-H), 6.77 (1H, d, *J* 15.4, 7-H), 7.27 (1H, m, ArH), 7.32-7.39 (2H, m, ArH), 7.55 (1H, dd, *J* 15.4, 11.5, C-6), 7.59-7.64 (2H, m, ArH), 8.06 (1H, d, *J* 15.5, 3-H); δ_C(100 MHz, $(CD_3)_2CO$) 20.3 (C-4), 119.6 (C-2), 124.3 (C-6), 127.8 (Ar), 129.0 (Ar), 129.6 (Ar), 133.0 (C-4), 136.4 (C-7), 137.9 (C-5), 138.1 (Ar), 141.0 (C-3), 168.1 (C-1); Found (ESI): 213.0915 $[M-H]^-$, (required $C_{14}H_{13}O_2$ 213.0916).

Strobilurin A 1[17,18]

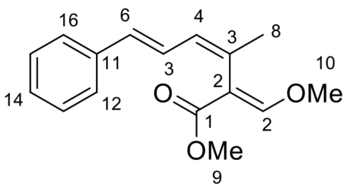

methyl (2*E*,3*Z*,5*E*)-2-(methoxymethylene)-3-methyl-6-phenylhexa-3,5-dienoate
Chemical Formula: $C_{16}H_{18}O_3$
Exact Mass: 258.1256

Isolation of **1** from raw extract (1 litre fermentation) of *A. oryzae* expression strain containing *stPKS, str11, str8, str10, str9* yielded in 2.6 mg of **1.** HRMS: measured 281.1157, calc for $C_{16}H_{18}O_3Na$ 281.1154. **^{1}H NMR** (500 MHz, $CDCl_3$), δ ppm 1.98 (s, 3H, H-8), 3.74 (s, 3H, H-9), 3.85 (s, 3H, H-10), 6.27 (m, 1H, H-4), 6.49 (1H, dd, *J* = 15.8, 0.8, H-6), 6.62 (1H, dd, *J* = 15.6, 10.7, H-5), 7.19 (1H, m, H-14), 7.28 (m, 2H, H-13, 15), 7.34 (2H, m, H-12, 16); **^{13}C NMR**, (101 MHz $CDCl_3$) δ ppm 23.8 (C-8), 51.8 (C-9), 62.1 (C-10), 111.0 (C-2), 126.5 (C-12, 16), 126.7 (C-5), 127.3 (C-14), 128.6 (C-13, 15), 129.9 (C-4), 131.3 (C-6), 131.5 (C-3), 138.0 (C-11), 159.0 (C-7), 168.0 (C-1).

Bolineol 8[19]

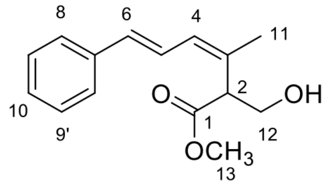

methyl (3*Z*,5*E*)-2-(hydroxymethyl)-3-methyl-6-phenylhexa-3,5-dienoate
Chemical Formula: $C_{15}H_{18}O_3$
Exact Mass: 246.1256
Bolineol

(3*Z*,5*E*)-2-(Hydroxymethyl)-3-methyl-6-phenylhexa-3,5-dienoic acid **22** (0.011 g, 0.048 mmol, 1.0 eq.) was treated with TMS-CHN_2 (0.016 g, 0.14 mmol, 3.0 eq., Sigma) in MeOH at RT

overnight. The solvent was removed *in vacuo* and the product was purified using preparative LCMS. **^{1}H NMR** (400 MHz, $CDCl_3$), δ ppm 1.81 (m, 3H, H-11), 3.74 (3H, s, H-13), 3.68 (1H, dd, *J* = 11.1, 5.5, H-2), 3.99 (1H, dd, *J* = 8.7, 5.5, H-12), 4.10 (1H, dd, *J* = 11.1, 8.7, H-12), 6.22 (1H, dq, *J* = 11.1, 1.3, 4-H), 6.55 (1H, d, *J* = 15.4, H-6), 7.00 (1H, dd, *J* = 15.3, 11.0, H-5), 7.23 (1H, m, H-10), 7.32 (m, 2H, H-8/8'), 7.41 (2H, m, H-9/9').

Prestrobilurin A 11

Pos.	δ_C / ppm 125 MHz acetone-d6	δ_H / ppm 500 MHz acetone-d6	COSY	HMBC	NOESY
1	168.1	-	-	-	-
2	119.6	6 (d, 1H, *J* = 15.5)	3	1, 4	-
3	141	8(d, 1H, *J* = 15.5)	2	1, 2, 4, 5, 14	6
4	132.3	-	-	-	-
5	137.9	6.5 (d, 1H, *J* = 12)	6	3, 7, 14	7, 14
6	124.3	7.5 (dd, 1H, *J* = 11.4, 15.2)	5, 7	4, 5	3
7	136.4	6.8 (d, 1H, *J* = 15.3)	6	5, 8, 13	5
8	138	-	-	-	-
9, 13	127.8	7.6 (d, 1H, *J* = 7.7)	12	7, 11, 12, 13	-
10, 12	129.6	7.4 (t, 1H, *J* = 7.7, 15.3)	11, 13	12	-
11	128.9	7.3 (t, 1H, *J* = 7, 14.3)	10, 12	13	-
14	20.1	2 (s, 3H)	5	3, 4, 5	5

(3*Z*,5*E*)-3-Methyl-6-phenylhexa-3,5-dienoic acid 21[20]

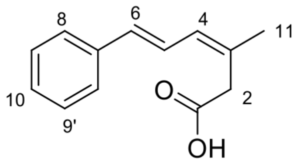

(3*Z*,5*E*)-3-methyl-6-phenylhexa-3,5-dienoic acid
Chemical Formula: $C_{13}H_{14}O_2$
Exact Mass: 202.0994
21

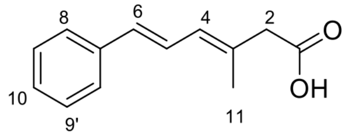

(3*E*,5*E*)-3-methyl-6-phenylhexa-3,5-dienoic acid
Chemical Formula: $C_{13}H_{14}O_2$
Exact Mass: 202.0994
21a

Isolation of **21** from raw extract (1 l fermentation) of *A. oryzae* expression strain containing *stPKS, str11, str8, str10, str9 and stl2* yielded in 90 mg of **21**. Structure elucidation revealed two isomers **21** and **21a** in the ratio 3:1 (determined by NMR). **HRMS** (ESI-) *m/z* calc. for $C_{13}H_{15}O_2$ [M]H$^+$ 203.1072, found 203.1071.

21: ^{1}H NMR (400 MHz, $CDCl_3$), δ ppm 1.96 (s, 3 H, H-11), 3.32, (s, 2 H, H-2), 6.22 (d, *J* = 10.8 Hz, 1 H, H-4), 6.53 (d, *J* = 15.4 Hz, 1 H, H-6), 6.96 (dd, *J* = 10.9 Hz, 15.5 Hz, 1 H, H-5), 7.22 (m, 1H, H-10), 7.31 (m, 2 H, H-8/8'), 7.40 (m, 2H, H-9/9'); **^{13}C NMR**, (101 MHz $CDCl_3$) δ ppm 24.7 (C-11), 38.0 (C-2), 124.4 (C-5), 126.5 (C-9/9'), 127.6 (C-10), 128.7 (C-8/8'), 129.6 (C-4), 130.7 (C-3), 132.5 (C-6), 137.6 (C-7), 177.2 (C-1).

21a: **^{1}H NMR** (400 MHz, $CDCl_3$), δ ppm 1.96 (s, 3 H, H-11), 3.16 (s, 2 H, H-2), 6.16 (d, *J* = 10.8 Hz, 1 H, H-4), 6.53 (d, *J* = 15.4 Hz, 1 H, H-6), 6.96 (dd, *J* = 10.9 Hz, 15.5 Hz, 1 H, H-5), 7.22 (m, 1H, H-10), 7.31 (m, 2 H, H-8/8'), 7.40 (m, 2H, H-9/9'); **^{13}C NMR**, (101 MHz $CDCl_3$) δ ppm 17.3 (C-11), 45.0 (C-2), 124.8 (C-5), 126.5 (C-9/9'), 127.6 (C-10), 128.7 (C-8/8'), 129.8 (C-4), 131.0 (C-3), 132.6 (C-6), 137.7 (C-7), 177.7 (C-1).

(3*Z*,5*E*)-2-(Hydroxymethyl)-3-methyl-6-phenylhexa-3,5-dienoic acid 22

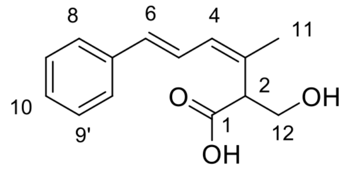

(3Z,5*E*)-2-(hydroxymethyl)-3-methyl-6-phenylhexa-3,5-dienoic acid
Chemical Formula: $C_{14}H_{16}O_3$
Exact Mass: 232.1099

Isolation of **22** from raw extract (1 l fermentation) of *A. oryzae* expression strain containing *stPKS, str11, str8, str10, str9 and stl2* yielded in 30 mg of **22.** HRMS (ESI-) *m/z* calc. for $C_{14}H_{15}O_3$ $[M-H]^-$ 231.1021, found 231.1021. **^{1}H NMR** (400 MHz, $CDCl_3$), δ ppm 1.86 (s, 3H, H-11), 4.08 (1H, m, H-2), 3.71 (1H, dd, H-12), 4.08 (1H, m, H-12), 6.24 (1H, d, *J* = 11.4, 4-H), 6.56 (1H, d, *J* = 15.3, H-6), 7.02 (1H, dd, *J* = 15.3, 11.0, H-5), 7.23 (1H, m, H-10), 7.31 (m, 2H, H-8/8'), 7.41 (2H, m, H-9/9'); **^{13}C NMR**, (101 MHz $CDCl_3$) δ ppm 21.3 (C-11), 49.9 (C-2), 61.9 (C-12), 123.6 (C-5), 126.6 (C-9/9'), 127.9 (C-10), 128.8 (C-8/8'), 131.1 (C-4), 130.9 (C-3), 133.5 (C-6), 137.3 (C-7), 177.9 (C-1).

Strobilurin Resistance Mechanism

The strobilurin fungicides target the mitochondrial cytochrome b gene. They block electron transfer at the site of quinol oxidation in the cytochrome bc1 complex (the Qo site), thus preventing ATP formation. Mutations in the cytochrome b gene have been shown to confer resistance in many important plant pathogens. The strongest and most common mutation encodes a change from glycine to alanine at amino acid position 143 (G143A).[21,22,23,24] A second point mutation which has been shown to confer resistance is a phenylalanine-to-leucine change (F129L).[23,25,26] The cytochrome b protein sequence from a strobilurin producing *S. tenacellus* strain was shown to be lacking both of these key residue changes.[27] The replacement of a small residue in position 254 by glutamine and Asn261 by aspartate were identified as the likely resistance mechanism.[27] Biolinux was used to search both genomes for the cytochrome b gene. Scaffold 91 and scaffold 121 of the *S. lutea* and *S.*

tenacellus genomes respectively were found to contain candidate sequences. These scaffolds were then analysed using the yeast mitochondrial genetic code to identify potential genes. Annotations were carried out manually based on the known *S. tenacellus* cytochrome b sequence. In both species, cytochrome b was found to consist of 5 exons with large introns (1-2 kb). The resulting *S. tenacellus* and *S. lutea* protein sequences have 96.9 % sequence identity with one another and 91% or 90% sequence identity with the publicly available *S. tenacellus* protein sequence respectively. As was the case with the previously investigated *S. tenacellus* strain,[27] the predicted protein sequences lacks either the G143A or F129L mutations, but both have glutamine in position 254 and an N261D substitution (Supplementary Figure 44).

The four large introns are type 1 introns; self-splicing introns that are common to fungal mitochondrial genes.[28,29] Each intron contains a putative Homing Endonuclease Gene (*heg*) (Supplementary Figures 46-48 and Supplementary Table 8). Homing endonucleases are double stranded DNAses which confer mobility to their host sequence. Multiple homing endonucleases, including those within cytochrome b genes, have been shown to also function as maturase proteins which assist in RNA splicing.[30,31,32]

Isolation of substrate 11

The substrate prestrobilurin A **11** was isolated from the expression strain *A. oryzae* NSAR1 *stPKS+str8+str10+str11* by preparative LC-MS as described before to yield 150 mg pure compound from 1 litre of culture.

Recombinant Str9 overproduction and purification

Recombinant N-terminal hexa-histidine tagged Str9 was produced by overexpression in BL21 (DE3) *E. coli* cells using the plasmid pET100-His6-Str9, which contains a codon optimised sequence for Str9. For overproduction single colonies were used to inoculate 2TY medium (16.0 g·L^{-1} tryptone, 10 g·L^{-1} yeast, 5 g·L^{-1} NaCl). Cultures were grown to an OD_{600} of 0.4 to 0.6 at 37 °C, induced with 0.1 mM IPTG and shaken at 16 °C for 20 h. Cells were harvested by centrifugation (4000 g, 4 °C, 15 min). For purification, 2 g cells were resuspended in 20 mL lysing buffer (50 mM Phosphate buffer, pH 8.0) and cell disruption was done by sonification. After centrifugation (20000 g, 4 °C, 40 min) the obtained crude lysate was passed through a gravimetric Ni-NTA column (2 mL bed volume). The column was washed with 5 mL of lysing buffer containing 20 mM imidazole. Elution of the target protein was achieved with 3 mL of lysing buffer containing 250 to 500 mM imidazole. The elution fractions were combined and concentrated (Satorius Vivaspin 20- 10000 MW cut-off) and imidazole was removed from the buffer. The purified enzyme was immediately used for activity assays.

SDS – Polyacrylamide gel electrophoresis (SDS–PAGE)

Separation and analysis of proteins was carried out using 12 % polyacrylamide gels. Protein samples were prepared with SDS loading buffer and boiled at 95 °C for 10 min before 20 µl of the samples and 5 µl of Color Prestained Protein Standard (Broad Range, 11—245 kDa, NEB) were loaded onto the gel (ran for 60 min at 40 mA). Afterwards gels were incubated in 30 mL Coomassie staining solution for 1 h before destaining in Coomassie bleach solution for another hour. Destained gels were scanned using the Molecular Imager Gel doc XR+ (Bio–Rad) system.

In vitro activity assay with purified Str9

Activity assays were carried out in a total volume of 400 µL containing 2 mM of prestrobilurin A **11** in 200 µl ethanol and 5 mg·mL^{-1} of Str9 in 200 µl phosphate buffer (pH 8.0, 50 mM phosphate buffer plus 1.25 mM NADH/NADPH mixture and 0.3 mM FAD, 2.5 mM FMN mixture). Reactions were incubated at 30 °C for 30 min at 400 rpm and then for 60 h at room temperature without shaking. Prior to extraction with 400 µl ethyl acetate, reactions were acidified with 2 M HCl to pH 4. After evaporation of the solvent, the product was redissolved in 150 µl DMSO and analysed by LCMS (Supplementary Figure 53). Reactions without enzyme served as controls and showed no conversion.

Supplementary References

1. Carver, T. J., Rutherford, K. M., Berriman, M., Rajandream, M. A., Barrell, B. G. & Parkhill, J. ACT: the Artemis Comparison Tool. *Bioinformatics* **21**, 3422-3 (2005).
2. Marchler-Bauer, A. *et al.* CDD/SPARCLE: functional classification of proteins via subfamily domain architectures, *Nucleic Acids Res.,* **45,** D200–D203 (2017).
3. Altschul, S. *et al.* Protein database searches using compositionally adjusted substitution matrices. *Febs J* **272,** 5101–5109 (2005).
4. Klodmann, J., Senkler, M., Rode, C. & Braun, H.-P. Defining the protein complex proteome of plant mitochondria. *Plant Physiol.* **157,** 587–598 (2011).
5. S. Sreenivasaprasad, in *The Nucleic Acid Protocols Handbook*, ed. R. Rapley, Humana Press, Totowa, N.J, 2000, pp. 37–45.
6. Garger, S. J., Griffith, O. M. & Grill, L. K. Rapid Purification of Plasmid DNA by a Single Centrifugation in a 2-Step Cesium-Chloride Ethidium-Bromide Gradient. *Biochem. Biophys. Res. Commun.* **117,** 835–842 (1983).
7. Blin, K. *et al.* antiSMASH 4.0—improvements in chemistry prediction and gene cluster boundary identification. *Nucleic Acids Res* 1–6 (2017).
8. Field, D., Tiwari, B., Booth, T., Houten, S., Swan, D., Bertrand, N. and Thurston, M. Open Software for biologists: from famine to feast. *Nature Biotechnol.* **24**, 801–803 (2006).
9. Solovyev, V., Kosarev, P., Seledsov, I. & Vorobyev, D. Automatic annotation of eukaryotic genes, pseudogenes and promoters. *Genome Biol.* **7 Suppl 1,** S10.1–12 (2006).

10. Zerbino, D. R. & Birney, E. Velvet: Algorithms for de novo short read assembly using de Bruijn graphs. *Genome Research* **18,** 821–829 (2008).

11. Rutherford, K. *et al.* Artemis: sequence visualization and annotation. *Bioinformatics* **16,** 944–945 (2000).

12. Carver, T., Harris, S. R., Berriman, M., Parkhill, J. & McQuillan, J. A. Artemis: an integrated platform for visualization and analysis of high-throughput sequence-based experimental data. *Bioinformatics* **28,** 464–469 (2012).

13. Tamura, K., Dudley, J., Nei, M. & Kumar, S. MEGA4: Molecular Evolutionary Genetics Analysis (MEGA) Software Version 4.0. *Mol. Biol. Evol.* **24,** 1596–1599 (2007).

14. Lazarus, C. M., Williams, K. & Bailey, A. M. Reconstructing fungal natural product biosynthetic pathways. *Nat. Prod. Rep.* **31,** 1339-1347 (2014).

15. Still, W., Kahn, M. & Mitra, A. Rapid Chromatographic Technique For Preparative Separations With Moderate Resolution. *J. Org. Chem.* **43,** 2923–2925 (1978).

16. Prantz, K. & Mulzer, J. Decarboxylative Grob-Type Fragmentations in the Synthesis of Trisubstituted Z Olefins: Application to Peloruside A, Discodermolide, and Epothilone D. *Angew. Chem. Int. Edit.* **48,** 5030–5033 (2009).

17. Becker, W., Vonjagow, G., Anke, T. & Steglich, W. Oudemansin, Strobilurin-A, Strobilurin-B and Myxothiazol - New Inhibitors of the Bc1 Segment of the Respiratory-Chain with an *E*-Beta-Methoxyacrylate System as Common Structural Element, *FEBS Lett.* **132,** 329–333 (1981).

18. Nerud, F., Sedmera, P., Zouchová, Z., Musílek, V. & Vondráček, M. Biosynthesis of mucidin, an antifungal antibiotic from Basidiomycete *Oudemansiella mucida* ^{2}H-, ^{13}C- and ^{14}C-labelling study, *Coll. Czech. Chem. Commun.,* **47,** 1020–1025 (1982).

19. Bedford, C. T., Perry, D. & Sharma, R. K. Bolineol, a novel co-metabolite of strobilurins A, F and G from *Bolinea lutea*, *Nat. Prod. Res.* **22,** 1535–1539 (2008).

20. Grigorieva, N. Y., Popovsky, V. A., Stepanov, A. V. & Lubuzh, E. D. Formal synthesis of strobilurins A and X. *Russ. Chem. Bull.,* **59,** 2086–2093 (2010).

21. Ishii, H. *et al.* Occurrence and molecular characterization of strobilurin resistance in cucumber powdery mildew and downy mildew. *Phytopathology* **91,** 1166–1171 (2001).

22. Sierotzki, H., Wullschleger, J. & Gisi, U. Point Mutation in Cytochrome b Gene Conferring Resistance to Strobilurin Fungicides in *Erysiphe graminis f. sp. tritici* Field Isolates. *Pestic Biochem Phys* **68,** 107–112 (2000).

23. Kim, Y. S., Dixon, E. W., Vincelli, P. & Farman, M. L. Field resistance to strobilurin (Q(o)I) fungicides in Pyricularia grisea caused by mutations in the mitochondrial cytochrome b gene. *Phytopathology* **93,** 891–900 (2003).

24. Meamiche Neddaf, H., Aouini, L., Bouznad, Z. & Kema, G. H. J. Equal Distribution of Mating Type Alleles and the Presence of Strobilurin Resistance in Algerian Zymoseptoria triticiField Populations. *Plant Disease* **101,** 544–549 (2017).

25. Sierotzki, H. *et al.* Cytochrome b gene sequence and structure of Pyrenophora teres and P. tritici-repentis and implications for QoI resistance. *Pest. Manag. Sci.* **63,** 225–233 (2007).

26. Pasche, J. S., Piche, L. M. & Gudmestad, N. C. Effect of the F129L Mutation in *Alternaria solanion* Fungicides Affecting Mitochondrial Respiration. *Plant Disease* **89,** 269–278 (2005).

27. Kraiczy, P. *et al.* The molecular basis for the natural resistance of the cytochrome bc(1) complex from strobilurin-producing Basidiomycetes to center Q(P) inhibitos. *Eur J Biochem* **235,** 54–63 (1996).

28. Lang, B. F., Laforest, M.-J. & Burger, G. Mitochondrial introns: a critical view. *Trends Genet.,* **23,** 119–125 (2007).

29. Cech, T. R. Self-Splicing of Group-I Introns. *Ann. Rev. Biochem.,* **59,** 543–568 (1990).

30. Bolduc, J. M. *et al.* Structural and biochemical analyses of DNA and RNA binding by a bifunctional homing endonuclease and group I intron splicing factor. *Genes Dev.* **17,** 2875–2888 (2003).

31. Schafer, B. *et al.* A Mitochondrial Group-I Intron in Fission Yeast Encodes a Maturase and Is Mobile in Crosses. *Curr Genet* **25,** 336–341 (1994).

32. Lazowska, J., Jacq, C. & Slonimski, P. P. Sequence of Introns and Flanking Exons in Wild-Type and Box3 Mutants of Cytochrome-B Reveals an Interlaced Splicing Protein Coded by an Intron. *Cell* **22,** 333–348 (1980).
